# Clustering on hierarchical heterogeneous data with prior pairwise relationships

**DOI:** 10.1186/s12859-024-05652-6

**Published:** 2024-01-23

**Authors:** Wei Han, Sanguo Zhang, Hailong Gao, Deliang Bu

**Affiliations:** 1 School of Mathematical Sciences, University of Chinese Academy of Sciences, Beijing, China; 2 Key Laboratory of Big Data Mining and Knowledge Management, Chinese Academy of Sciences, Beijing, China; 3 School of Mathematics and Statistics, Qingdao University, Qingdao, China; 4 School of Statistics, Capital University of Economics and Business, Beijing, China

**Keywords:** Cancer clustering, Hierarchy, Heterogeneity, Prior pairwise relationships

## Abstract

**Background:**

Clustering is a fundamental problem in statistics and has broad applications in various areas. Traditional clustering methods treat features equally and ignore the potential structure brought by the characteristic difference of features. Especially in cancer diagnosis and treatment, several types of biological features are collected and analyzed together. Treating these features equally fails to identify the heterogeneity of both data structure and cancer itself, which leads to incompleteness and inefficacy of current anti-cancer therapies.

**Objectives:**

In this paper, we propose a clustering framework based on hierarchical heterogeneous data with prior pairwise relationships. The proposed clustering method fully characterizes the difference of features and identifies potential hierarchical structure by rough and refined clusters.

**Results:**

The refined clustering further divides the clusters obtained by the rough clustering into different subtypes. Thus it provides a deeper insight of cancer that can not be detected by existing clustering methods. The proposed method is also flexible with prior information, additional pairwise relationships of samples can be incorporated to help to improve clustering performance. Finally, well-grounded statistical consistency properties of our proposed method are rigorously established, including the accurate estimation of parameters and determination of clustering structures.

**Conclusions:**

Our proposed method achieves better clustering performance than other methods in simulation studies, and the clustering accuracy increases with prior information incorporated. Meaningful biological findings are obtained in the analysis of lung adenocarcinoma with clinical imaging data and omics data, showing that hierarchical structure produced by rough and refined clustering is necessary and reasonable.

**Supplementary information:**

The online version contains supplementary material available at (10.1186/s12859-024-05652-6).

## Introduction

Clustering is a fundamental problem in unsupervised learning, which aims to group objects of similar kind into respective categories. It has broad applications in different areas such as finance [1, 2], machine learning [3, 4], and molecular biology [5]. Classic clustering methods include: *K*-means clustering, hierarchical clustering, DBSCAN, and Gaussian mixture models. See a brief overview of these methods in [6].

Traditional clustering methods treat all features equally and perform algorithm on all dimensions. As the world steadily becomes more connected with an ever-increasing number of electronic devices, the dimension of data grows rapidly. Traditional clustering methods may not be sufficient for the high-dimensional and complex data structure. In the area of supervised learning, lots of methods such as lasso [7] and others [8–10] have been proposed to deal with these situations. Similar to supervised learning methods, [11] has proposed a clustering algorithm to deal with complex data structure in unsupervised learning. They assumed that in high-dimensional scenario, some of features may be non-informative and only part of features should be used in clustering procedure. Thus, they combined convex clustering method [12] with group-lasso penalty [10], and proposed sparse convex clustering to conduct clustering and variable selection at the same time. Different from the sparse assumption, in this paper, we focus on developing clustering algorithm of somehow different data structure which is commonly encountered in the area of complex diseases.

Heterogeneity is one of the most important hallmarks in complex diseases [13], especially cancers [14]. Human cancers exhibit formidable molecular heterogeneity, to a large extent accounting for the incompleteness and transitory efficacy of current anti-cancer therapies [15]. Clustering can help to find subtypes of diseases in the context of precision medicine [16], specific designed treatments based on these subtypes can further improve cancer survival. The idea of obtaining more refined cancer heterogeneity structure with the increased resolution of information/technique is not new. Take breast cancer as an example. In the past, with information/technique limitations, it was considered as a single disease. With the development of high-throughput profiling and information contained in gene expressions, it was separated into five subtypes: Luminal A, Luminal B, HER2-enriched, Triple-negative, and Claudin-low [17]. Further advancements in sequencing have suggested that these subtypes may contain finer structures. For example, a recent study [18] suggests that the Triple-negative subtype can be further separated into three sub-subtypes (Lipogenic, Glycolytic, and Mixed). Besides breast cancer, lung cancer also has heterogeneity and is very challenging for diagnosis [19] and drug development [20].

Not only the diseases, the data used in disease analysis also have heterogeneous structure. That is, different types of features may represent different aspect of data and can be used for different goals. For example, clinical imaging data including magnetic resonance images (MRI), computed tomography (CT) scans, positron emission tomography (PET), and mammographic images [21], are routinely ordered for cancer and suspicious patients for their quicker screening and less expense [22]. One important advantage of clinical imaging data is that imaging provides a global, unbiased view of the entire tumor as well as its surrounding tissue [23]. On the other hand, omics data, including genomics, transcriptomics, and methylomics data, have also been broadly used in discovering modules of co-regulated genes and finding subtypes of diseases in the context of precision medicine [16]. The availability of large-scale omics datasets has spurred a significant interest in linking tumor phenotypes at molecular level, leading to an improved understanding of the molecular mechanisms behind imaging datasets [23]. To summarize, clinical imaging data provide a global view with rough information while omics data provide a more detailed and refined structure of the disease. Combining analysis of these two kinds of data may further improve the understanding of cancer and other complex diseases. In this paper, we assume that the clustering problem has a hierarchical structure, that is, the rough information divides samples into different types while the refined information works beneath the rough information and further divides particular type defined by the rough information into numbers of subtypes while preserving the original rough structure.

Another aspect of our proposed method is motivated by real data. Recent advances of data sharing make it increasingly available to gain additional information for data analysis. In the context of genome wide association analysis (GWAS), summary statistics generated from external datasets with large sample size can be used to aid the analysis of internal data [24]. Another example is that, in the area of machine learning, semi-supervised learning methods combine a small amount of human-labeled data (exclusively used in more expensive and time-consuming supervised learning paradigms), followed by a large amount of unlabeled data. This paradigm of using external data has been proved to increase the accuracy of prediction and clustering [25]. Here in our real data analysis, a small amount of the data are collected with additional clinical biological variables. Thus, to fully use the external information and make our method more flexible, we first extract prior information, and then incorporate it into our clustering problem.

In this paper, we conduct hierarchical heterogeneity analysis of clinical imaging data and omics data with prior information incorporated. Different from existing methods treating all features equally, we define hierarchical structure based on the difference of features. The first type of clinical imaging data define a rough clustering structure and the second type of omics data define a refined clustering structure. This study contributes beyond the existing literature in following ways. First, an innovative clustering framework of joint analysis of hierarchical heterogeneous data is developed as well as an efficient ADMM algorithm. Second, prior knowledge extracted from additional variables can be flexibly used and help to improve clustering performance to a great extent. Third, the much-desired and well-grounded statistical consistency properties of our method are rigorously established, including the accurate estimation of parameters and determination of clustering structures. Last but not the least, the application of our method on lung adenocarcinoma potentially provides a more effective way for exploring valuable insights on precision medicine and disease diagnosis from multi-type biological datasets.

## Methodology

### Access to prior information

Before we introduce our clustering algorithm, we first give the definition of prior information. In reality, although we may not have a clear picture of the overall clustering structure of all subjects, local structure may be obtained with a small number of samples, which is based on manual labeling or pre-training of some existing methods on additional beneficial variables contained in part of samples. Based on such additional information, we can accurately extract the pairwise relationships between corresponding samples, specifically, whether a certain two subjects are in the same cluster. Consider *n* subjects whose indexes are $\left\{ 1,\ldots ,n\right\}$, we denote $\mathcal {A}=\left\{ \left( j,m\right) : 1\leqslant j <m\leqslant n\right\}$ as the set of all pairwise relationships. It is straight forward to see that whole pairwise relationships contain $\left| \mathcal {A}\right| =\frac{1}{2}n(n-1)$ elements, where $\left| \cdot \right|$ is the cardinality of the set. The extracted prior information $\mathcal {A}^{\textrm{p}}$ is a subset of $\mathcal {A}$, which indicates a set of some pairwise subject indexes satisfying the following two conditions.

1. If $\left( j,m\right) \in \mathcal {A}^{\textrm{p}}$, then the *j*-th and *m*-th subjects are in the same cluster, and $j<m$.
2. If $\left( j_{1},j_{2}\right) \in \mathcal {A}^{\textrm{p}}$, $\left( j_{2},j_{3}\right) \in \mathcal {A}^{\textrm{p}}$, then $\left( j_{1},j_{3}\right) \in \mathcal {A}^{\textrm{p}}$. If $\left( j_{1},m\right) \in \mathcal {A}^{\textrm{p}}$, $\left( j_{2},m\right) \in \mathcal {A}^{\textrm{p}}$, and $j_{1}<j_{2}$, then $\left( j_{1},j_{2}\right) \in \mathcal {A}^{\textrm{p}}$. If $\left( j,m_{1}\right) \in \mathcal {A}^{\textrm{p}}$, $\left( j,m_{2}\right) \in \mathcal {A}^{\textrm{p}}$, and $m_{1}<m_{2}$, then $\left( m_{1},m_{2}\right) \in \mathcal {A}^{\textrm{p}}$.

The first condition implies that the element in $\mathcal {A}^{\textrm{p}}$ indicates an prior belief on their belonging to the same cluster, and they form pairwise relationship according to natural order. The second condition implies that $\mathcal {A}^{\textrm{p}}$ holds transitivity and ensures that prior pairwise relationships do not contradict themselves. It should be noted that only small proportion of samples contain such prior information. For example, in our real data analysis, prior information is available for 51 of the 355 patients. We can further transform these pairwise prior information $\mathcal {A}^{\textrm{p}}$ contained in these samples to clustering structure, denoted by $\left\{ \mathcal {F}_{1},\ldots ,\mathcal {F}_{K}\right\}$, as a assistance for our clustering algorithm. When the prior information is not available, $\mathcal {A}^{\textrm{p}}=\emptyset$, then the clustering structure defined by $\mathcal {A}^{\textrm{p}}$ is $\left\{ \left\{ 1\right\} ,\left\{ 2\right\} ,\ldots ,\left\{ n\right\} \right\}$ and $K=n$. Thus, the lack of prior assistance is also included in our consideration as a special case.

**A small example**. Let $n=8$ and $\mathcal {A}^{\textrm{p}}=\left\{ (1,2),(1,4),(2,4),(5,6)\right\}$, then the prior clustering structure defined by $\mathcal {A}^{\textrm{p}}$ is $\left\{ \left\{ 1,2,4\right\} ,\left\{ 3\right\} ,\left\{ 5,6\right\} ,\left\{ 7\right\} ,\left\{ 8\right\} \right\}$ and $K=5$.

### Hierarchical penalties with prior information incorporated

Consider *n* independent subjects $\left\{ \varvec{X}_{i},\varvec{Z}_{i}\right\} _{i=1}^{n}$, where $\varvec{X}_{i}=\left( X_{i1},\ldots ,X_{iq}\right) ^{\textrm{T}}$ are *q*-dimensional features and $\varvec{Z}_{i}=\left( Z_{i1},\ldots ,Z_{ip}\right) ^{\textrm{T}}$ are *p*-dimensional features. In the context of cancer clustering, the first type of features $\varvec{X}$ are clinical imaging data, while the second type of features $\varvec{Z}$ are omics data and have relatively high dimension. Cancer clustering aims to divide the *n* subjects into several clusters, thus obtains potential patterns of cancers to further develop specific treatment of different types of cancers. In our medical research, the first type of features have intuitively biological meanings at clinical level, while the second type of features at molecular level are determined to be more informative. Hence they are validated to be hierarchical [26], where a rough clustering structure can be identified by $\varvec{X}$, and a refined clustering structure can be identified by $\varvec{Z}$. For the *i*-th subject, $\varvec{\beta }_{i}=\left( \beta _{i1},\ldots ,\beta _{iq}\right) ^{\textrm{T}}$ and $\varvec{\gamma }_{i}=\left( \gamma _{i1},\ldots ,\gamma _{ip}\right) ^{\textrm{T}}$ are denoted as the clustering centers (parameters) of the rough and refined clusters which the *i*-th subject belongs to, respectively. Denote $\varvec{\beta }=\left( \varvec{\beta }_{1},\ldots ,\varvec{\beta }_{n}\right) \in \mathbb {R}^{q}\times \mathbb {R}^{n}$ and $\varvec{\gamma }=\left( \varvec{\gamma }_{1},\ldots ,\varvec{\gamma }_{n}\right) \in \mathbb {R}^{p}\times \mathbb {R}^{n}$ are matrices of clustering parameters. Note that each subject is flexibly modeled to have its own clustering center, and two subjects belong to the same rough/refined cluster if and only if they have the same rough/refined clustering center. By prior information suggested in section “Access to prior information”, define the following two constraint parameter sets,

$$\begin{aligned}&\mathcal {M}_{1}=\left\{ \varvec{\beta }\in \mathbb {R}^{q}\times \mathbb {R}^{n}:\varvec{\beta }_{j}=\varvec{\beta }_{m}, \left( j,m\right) \in \mathcal {A}^{\textrm{p}}\right\} ,\\&\mathcal {M}_{2}=\left\{ \varvec{\gamma }\in \mathbb {R}^{p}\times \mathbb {R}^{n}:\varvec{\gamma }_{j}=\varvec{\gamma }_{m}, \left( j,m\right) \in \mathcal {A}^{\textrm{p}}\right\} . \end{aligned}$$

We propose a prior-incorporated clustering model with hierarchical penalties (PCH) by minimizing the following objective function,

2.1 $$\begin{aligned} Q\left( \varvec{\beta },\varvec{\gamma }\right) =&\frac{1}{2}\sum _{i=1}^{n}\left( \left\| \varvec{X}_{i}-\varvec{\beta }_{i}\right\| _{2}^{2}+\left\| \varvec{Z}_{i}-\varvec{\gamma }_{i}\right\| _{2}^{2}\right) \\&+\sum _{\left( j,m\right) \in \mathcal {A}\setminus \mathcal {A}^{\textrm{p}}}p\left( \left( \left\| \varvec{\beta }_{j}-\varvec{\beta }_{m}\right\| _{2}^{2}+\left\| \varvec{\gamma }_{j}-\varvec{\gamma }_{m}\right\| _{2}^{2}\right) ^{\frac{1}{2}};\lambda _{1}\right) \\&+\sum _{\left( j,m\right) \in \mathcal {A}\setminus \mathcal {A}^{\textrm{p}}}p\left( \left\| \varvec{\beta }_{j}-\varvec{\beta }_{m}\right\| _{2};\lambda _{2}\right) , \end{aligned}$$

subject to $\varvec{\beta }_{j}-\varvec{\beta }_{m}=0$ and $\varvec{\gamma }_{j}-\varvec{\gamma }_{m}=0$ when $\left( j,m\right) \in \mathcal {A}^{\textrm{p}}$, where $p\left( \cdot ;\lambda \right)$ is the concave penalty with tuning parameter $\lambda$. Note that the first term in (2.1) is similar to traditional convex clustering methods [12, 27–29] while the second and the third term are the penalties that guarantee hierarchical structure [30]. In our implementation, we adopt the minimax concave penalty (MCP; [9]). It is noted that the smoothly clipped absolute deviation penalty (SCAD; [8]) and some alternatives are equally applicable.

We obtain $\left( \widehat{\varvec{\beta }},\widehat{\varvec{\gamma }}\right)$ by minimizing (2.1) subject to $\varvec{\beta }\in \mathcal {M}_{1}$ and $\varvec{\gamma }\in \mathcal {M}_{2}$. Denote $\widehat{\varvec{\xi }}=\left( \widehat{\varvec{\xi }}_{1},\ldots ,\widehat{\varvec{\xi }}_{\widehat{K}_{1}}\right)$ and $\widehat{\varvec{\alpha }}=\left( \widehat{\varvec{\alpha }}_{1},\ldots ,\widehat{\varvec{\alpha }}_{\widehat{K}_{2}}\right)$ as the distinct values of $\widehat{\varvec{\beta }}$ and $\widehat{\varvec{\gamma }}$, respectively. Then the number of rough clusters $\widehat{K}_{1}$ and the rough clustering structure $\left\{ \widehat{\mathcal {G}}_{1},\ldots ,\widehat{\mathcal {G}}_{\widehat{K}_{1}}\right\}$ are determined by checking the distinct values of $\widehat{\varvec{\beta }}$, where $\left\{ \widehat{\mathcal {G}}_{1},\ldots ,\widehat{\mathcal {G}}_{\widehat{K}_{1}}\right\}$ constitutes mutually exclusive partitions of $\left\{ 1,\ldots ,n\right\}$ with $\widehat{\mathcal {G}}_{k_{1}}=\left\{ i:\widehat{\varvec{\beta }}_{i}=\widehat{\varvec{\xi }}_{k_{1}},i=1,\ldots ,n\right\}$ for $k_{1}=1,\ldots ,\widehat{K}_{1}$. Accordingly, the number of refined clusters $\widehat{K}_{2}$ and the refined clustering structure $\left\{ \mathcal {\widehat{T}}_{1},\ldots ,\mathcal {\widehat{T}}_{\widehat{K}_{2}}\right\}$ are determined by checking the distinct values of $\widehat{\varvec{\gamma }}$, where $\left\{ \mathcal {\widehat{T}}_{1},\ldots ,\mathcal {\widehat{T}}_{\widehat{K}_{2}}\right\}$ constitutes mutually exclusive partitions of $\left\{ 1,\ldots ,n\right\}$ with $\mathcal {\widehat{T}}_{k_{2}}=\left\{ i:\widehat{\varvec{\gamma }}_{i}=\widehat{\varvec{\alpha }}_{k_{2}},i=1,\ldots ,n\right\}$ for $k_{2}=1,\ldots ,\widehat{K}_{2}$. Moreover, $\widehat{\varvec{\xi }}_{k_{1}}$ is the estimated clustering center of the $k_{1}$-th cluster of the rough clustering structure, and $\widehat{\varvec{\alpha }}_{k_{2}}$ is the estimated clustering center of the $k_{2}$-th cluster of the refined clustering structure.

It is noted that $\lambda _{1}$ and $\lambda _{2}$ control the number of estimated clusters. When $\lambda _{1}$ and $\lambda _{2}$ are large enough, all clustering parameters tends to be equal, leading to all subjects belong to one cluster. When $\lambda _{1}$ and $\lambda _{2}$ are close to 0, the hierarchical penalties may slightly influence on $Q\left( \varvec{\beta },\varvec{\gamma }\right)$, then all subjects tend to be in separate clusters. To gain more insight into such characteristics, $\widehat{K}_{1}\left( \lambda _{1},\lambda _{2}\right)$ and $\widehat{K}_{2}\left( \lambda _{1},\lambda _{2}\right)$ can be seen as functions of $\lambda _{1}$ and $\lambda _{2}$, respectively. For one simulated data in section “Simulation studies”, as shown in Additional file 1: Figure S7, we observe how tuning parameters affect the number of estimated hierarchical clusters. The tuning procedure is well-behaved and recovers the true numbers of clusters $\left( \widehat{K}_{1},\widehat{K}_{2}\right) =(3,6)$ with optimized $\left( \lambda _{1},\lambda _{2}\right)$.

It should be especially noted that hierarchy is guaranteed indeed by the hierarchical penalties. For the *j*-th and *m*-th subjects, the term related to $\varvec{\gamma }_{j}-\varvec{\gamma }_{m}$ only appears in the first group penalty. Due to “all in or all out” property of the group penalty, the case with $\widehat{\varvec{\beta }}_{j}\ne \widehat{\varvec{\beta }}_{m}$ and $\widehat{\varvec{\gamma }}_{j}= \widehat{\varvec{\gamma }}_{m}$ cannot happen. Thus, the case with $\widehat{\varvec{\beta }}_{j}= \widehat{\varvec{\beta }}_{m}$ and $\widehat{\varvec{\gamma }}_{j}= \widehat{\varvec{\gamma }}_{m}$ leads them to be assigned to the same rough cluster and the same refined cluster, the case with $\widehat{\varvec{\beta }}_{j}= \widehat{\varvec{\beta }}_{m}$ and $\widehat{\varvec{\gamma }}_{j}\ne \widehat{\varvec{\gamma }}_{m}$ leads them to be assigned to the same rough cluster and different refined clusters, and the case with $\widehat{\varvec{\beta }}_{j}\ne \widehat{\varvec{\beta }}_{m}$ and $\widehat{\varvec{\gamma }}_{j}\ne \widehat{\varvec{\gamma }}_{m}$ leads them to be assigned to different rough clusters and different refined clusters. In conclusion, if two subjects are assigned to the same refined cluster, they must be assigned to the same rough cluster, which implies that the estimated refined clustering structure is exactly nested in the rough clustering structure. It should be noted that although no clustering methods produce hierarchical structure to the best of our knowledge, this hierarchy can be done by performing traditional convex clustering method twice, named as two-step clustering. To be more specific, two-step clustering first performs clustering based on $\varvec{X}$ with methods like convex clustering. Then second step clustering can be done based on $\varvec{Z}$ within each identified cluster of the first step. Compared to the aforementioned method, using hierarchical penalties is more informative since it only needs one step clustering and combines the information of $\varvec{X}$ and $\varvec{Z}$ while two-step clustering only uses the information within $\varvec{X}$ and $\varvec{Z}$. We will also demonstrate the supreme of clustering with hierarchical penalties over two-step clustering in our simulation studies.

## Statistical properties

Denote $\left\{ \mathcal {G}_{1}^{*},\ldots ,\mathcal {G}_{K_{1}}^{*}\right\}$ and $\left\{ \mathcal {T}_{1}^{*},\ldots ,\mathcal {T}_{K_{2}}^{*}\right\}$ as the true rough and refined clustering structure of the independent *n* subjects, respectively. Denote $\varvec{\xi }_{k_{1}}^{*}$ as the center of the $k_{1}$-th rough cluster for $k_{1}=1,\ldots ,K_{1}$. Denote $\varvec{\alpha }_{k_{2}}^{*}$ as the center of the $k_{2}$-th refined cluster for $k_{2}=1,\ldots ,K_{2}$. For the *i*-th subject, define $\varvec{\beta }_{i}^{*}=\varvec{\xi }_{k_{1}}^{*}$ and $\varvec{\gamma }_{i}^{*}=\varvec{\alpha }_{k_{2}}^{*}$ if *i* belongs to $\mathcal {G}_{k_{1}}^{*}$ and $\mathcal {T}_{k_{2}}^{*}$. We assume that $\varvec{X}_{i}=\varvec{\beta }_{i}^{*}+\varvec{\epsilon }_{1i}$ and $\varvec{Z}_{i}=\varvec{\gamma }_{i}^{*}+\varvec{\epsilon }_{2i}$, where $\varvec{\epsilon }_{i}=\left( \varvec{\epsilon }_{1i}^{\textrm{T}},\varvec{\epsilon }_{2i}^{\textrm{T}}\right) ^{\textrm{T}}$ is a random error vector with $\textrm{E}\left( \varvec{\epsilon _{i}}\right) =\varvec{0}$ and $\textrm{Var}\left( \varvec{\epsilon _{i}}\right) =\varvec{\Sigma }$. By the hierarchical structure, there exists a partition of $\left\{ 1,\ldots ,K_{2}\right\}$ denoted by $\left\{ \mathcal {H}_{1}^{*},\ldots ,\mathcal {H}_{K_{1}}^{*}\right\}$ satisfying $\mathcal {G}_{k_{1}}^{*}=\cup _{k_{2}\in \mathcal {H}_{k_{1}}^{*}}\mathcal {T}_{k_{2}}^{*},k_{1}=1,\ldots ,K_{1}$. We define the minimal differences of the centers between two rough and refined clusters as

$$\begin{aligned} b_{n}= & {} \textrm{min}_{j\in \mathcal {G}_{k_{1}}^{*},m\in \mathcal {G}_{k_{1}^{\prime }}^{*},1\leqslant k_{1}\ne k_{1}^{\prime }\leqslant K_{1}}\left\| \varvec{\beta }_{j}^{*}-\varvec{\beta }_{m}^{*}\right\| _{2}=\textrm{min}_{1\leqslant k_{1}\ne k_{1}^{\prime }\leqslant K_{1}}\left\| \varvec{\xi }_{k_{1}}^{*}-\varvec{\xi }_{k_{1}^{\prime }}^{*}\right\| _{2}, \\ d_{n}= & {} \textrm{min}_{j\in \mathcal {T}_{k_{2}}^{*},m\in \mathcal {T}_{k_{2}^{\prime }}^{*},1\leqslant k_{2}\ne k_{2}^{\prime }\leqslant K_{2}}\left\| \varvec{\gamma }_{j}^{*}-\varvec{\gamma }_{m}^{*}\right\| _{2}=\textrm{min}_{1\leqslant k_{2}\ne k_{2}^{\prime }\leqslant K_{2}}\left\| \varvec{\alpha }_{k_{2}}^{*}-\varvec{\alpha }_{k_{2}^{\prime }}^{*}\right\| _{2}. \end{aligned}$$

Moreover, we define the minimum of subject numbers of rough and refined clusters as

$$\begin{aligned} G_{\textrm{min}}=\textrm{min}_{1\leqslant k_{1}\leqslant K_{1}}\left| \mathcal {G}_{k_{1}}^{*}\right| ,\quad T_{\textrm{min}}=\textrm{min}_{1\leqslant k_{2}\leqslant K_{2}}\left| \mathcal {T}_{k_{2}}^{*}\right| . \end{aligned}$$

In our theoretical properties establishment, we assume some mild conditions.

### Condition 1

The random error vectors $\left\{ \varvec{\epsilon }_{i}\right\} _{i=1}^{n}=\left\{ \left( \varvec{\epsilon }_{1i}^{\textrm{T}},\varvec{\epsilon }_{2i}^{\textrm{T}}\right) ^{\textrm{T}}\right\} _{i=1}^{n}$ independently follow sub-Gaussian distribution with variance proxy $\sigma _{0}^{2}$, where $\sigma _{0}$ is a finite positive constant.

### Condition 2

The penalty $p\left( t;\lambda \right)$ is non-decreasing and concave on $[0,\infty )$. There exists a constant $a>0$ such that $p\left( t;\lambda \right)$ is a constant for all $t\geqslant a\lambda$, and $p\left( 0;\lambda \right) =0$. The derivative $p^{\prime }\left( t;\lambda \right)$ is continuous, bounded by $\lambda$ and satisfies $\lim _{t\rightarrow 0^{+}}p^{\prime }\left( t;\lambda \right) =\lambda$.

### Theorem 1

Suppose that $T_{\textrm{min}}\gg (q+p)\log n$ and Conditions 1-2 hold. If $\lambda _{1}$ and $\lambda _{2}$ are chosen satisfying that

$$\begin{aligned} \lambda _{1}<\left( a+\kappa \right) ^{-1}d_{n},\quad \lambda _{2}<\left( a+\kappa \right) ^{-1}b_{n}, \quad \min \left\{ \lambda _{1},\lambda _{2}\right\} \gg \phi _{n}, \end{aligned}$$

where $\phi _{n}=(q+p)^{\frac{1}{2}}T_{\textrm{min}}^{-\frac{1}{2}}\left( \log n\right) ^{\frac{1}{2}}$ and $\kappa$ is an arbitrary positive constant. As $n\rightarrow \infty$, there exists a local minimizer $\left( \widehat{\varvec{\beta }},\widehat{\varvec{\gamma }}\right)$ of $Q\left( \varvec{\beta },\varvec{\gamma }\right)$ subject to $\varvec{\beta }\in \mathcal {M}_{1}$ and $\varvec{\gamma }\in \mathcal {M}_{2}$ such that

(1) (Parameters estimation consistency)

$$\begin{aligned} \sup _{1\leqslant k_{1}\leqslant K_{1}}\sup _{i\in \mathcal {G}_{k_{1}}^{*}}\left\| \widehat{\varvec{\beta }}_{i}-\varvec{\xi }_{k_{1}}^{*}\right\| _{2}+\sup _{1\leqslant k_{2}\leqslant K_{2}}\sup _{i\in \mathcal {T}_{k_{2}}^{*}}\left\| \widehat{\varvec{\gamma }}_{i}-\varvec{\alpha }_{k_{2}}^{*}\right\| _{2}=O_{p}\left( \phi _{n}\right) . \end{aligned}$$

(2) (Clustering structures consistency)

$$\begin{aligned}&\textrm{Pr}\left( \widehat{K}_{1}=K_{1}\right) \rightarrow 1,\quad \textrm{Pr}\left( \widehat{\mathcal {G}}_{k_{1}}=\mathcal {G}_{k_{1}}^{*},\ k_{1}=1,\ldots ,K_{1}\right) \rightarrow 1,\\&\textrm{Pr}\left( \widehat{K}_{2}=K_{2}\right) \rightarrow 1,\quad \textrm{Pr}\left( \widehat{\mathcal {T}}_{k_{2}}=\mathcal {T}_{k_{2}}^{*},\ k_{2}=1,\ldots ,K_{2}\right) \rightarrow 1. \end{aligned}$$

Theorem 1 has demonstrated the much-desired consistency properties of our proposed method. Condition 1 assumes clustering noises to follow sub-Gaussian distribution, which is widely seen in high-dimensional statistical analysis and fusion-based clustering analysis [11, 28, 31–34]. Condition 2 is a common assumption in penalization-based methods [8–10], and our adopted MCP is applicable. With sample sizes nearly balanced among refined clusters and far greater than the dimension of features, still allowing $q+p$ to tend to infinity, the convergence rate $\phi _{n}\rightarrow 0$. As a result, with proper parameters, the model can accurately determine the number of rough and refined clusters, and reliably reconstruct their corresponding structures with high probability. In addition, the rough and refined center estimation consistency is well-established. Different from the existing convex clustering framework which adopts a single-level penalty, the theoretical development presents significant complexity and challenges. The proof is available in Additional file 1.

## Computational algorithm

We derive an ADMM algorithm for optimizing the objective function. By introducing two new sets of parameters $\varvec{\omega }=\left\{ \varvec{\omega }_{jm}, \left( j,m\right) \in \mathcal {A}\right\}$ and $\varvec{\eta }=\left\{ \varvec{\eta }_{jm},\left( j,m\right) \in \mathcal {A}\right\}$, minimization of objective function is equivalent to the following constrained minimization problem,

$$\begin{aligned} \mathcal {L}_{0}\left( \varvec{\beta },\varvec{\gamma },\varvec{\omega },\varvec{\eta }\right)&=\frac{1}{2}\sum _{i=1}^{n}\left( \left\| \varvec{X}_{i}-\varvec{\beta }_{i}\right\| _{2}^{2}+\left\| \varvec{Z}_{i}-\varvec{\gamma }_{i}\right\| _{2}^{2}\right) \\&+\sum _{\left( j,m\right) \in \mathcal {A}}p\left( \left( \left\| \varvec{\omega }_{jm}\right\| _{2}^{2}+\left\| \varvec{\eta }_{jm}\right\| _{2}^{2}\right) ^{\frac{1}{2}};\lambda _{1}\right) +\sum _{\left( j,m\right) \in \mathcal {A}}p\left( \left\| \varvec{\omega }_{jm}\right\| _{2};\lambda _{2}\right) ,\\ \mathrm {s.t.}\quad&\varvec{\beta }_{j}-\varvec{\beta }_{m}-\varvec{\omega }_{jm}=0,\ \varvec{\gamma }_{j}-\varvec{\gamma }_{m}-\varvec{\eta }_{jm}=0,\ \left( j,m\right) \in \mathcal {A},\\&\varvec{\beta }_{j}-\varvec{\beta }_{m}=0,\ \varvec{\gamma }_{j}-\varvec{\gamma }_{m}=0,\ \left( j,m\right) \in \mathcal {A}^{\textrm{p}}. \end{aligned}$$

Then the augmented Lagrangian function is

$$\begin{aligned} \mathcal {L}\left( \varvec{\beta },\varvec{\gamma },\varvec{\omega },\varvec{\eta },\varvec{v},\varvec{u}\right) =&\mathcal {L}_{0}\left( \varvec{\beta },\varvec{\gamma },\varvec{\omega },\varvec{\eta }\right) \\&+\sum _{\left( j,m\right) \in \mathcal {A}}\varvec{v}_{jm}^{\textrm{T}}\left( \varvec{\beta }_{j}-\varvec{\beta }_{m}-\varvec{\omega }_{jm}\right) +\frac{\vartheta }{2}\sum _{\left( j,m\right) \in \mathcal {A}}\left\| \varvec{\beta }_{j}-\varvec{\beta }_{m}-\varvec{\omega }_{jm}\right\| _{2}^{2}\\&+\sum _{\left( j,m\right) \in \mathcal {A}}\varvec{u}_{jm}^{\textrm{T}}\left( \varvec{\gamma }_{j}-\varvec{\gamma }_{m}-\varvec{\eta }_{jm}\right) +\frac{\vartheta }{2}\sum _{\left( j,m\right) \in \mathcal {A}}\left\| \varvec{\gamma }_{j}-\varvec{\gamma }_{m}-\varvec{\eta }_{jm}\right\| _{2}^{2}, \end{aligned}$$

subject to $\varvec{\beta }_{j}-\varvec{\beta }_{m}=0$, $\varvec{\gamma }_{j}-\varvec{\gamma }_{m}=0$, $\varvec{\omega }_{jm}=0$, and $\varvec{\eta }_{jm}=0$, when $\left( j,m\right) \in \mathcal {A}^{\textrm{p}}$. The dual variables $\varvec{v}=\left\{ \varvec{v}_{jm}, \left( j,m\right) \in \mathcal {A}\right\}$ and $\varvec{u}=\left\{ \varvec{u}_{jm}, \left( j,m\right) \in \mathcal {A}\right\}$ are the Lagrange multipliers, $\varvec{v}_{jm}$ and $\varvec{u}_{jm}$ are *q*- and *p*-dimensional vectors, and $\vartheta$ is a fixed ADMM algorithm penalty parameter. Then the standard ADMM optimization procedures [35] can be applied to find the local minimizer of $\mathcal {L}\left( \varvec{\beta },\varvec{\gamma },\varvec{\omega },\varvec{\eta },\varvec{v},\varvec{u}\right)$. For initial values, we adopt a two-step *K*-means method and incorporate prior information. We first capture a rough clustering structure by *K*-means method, and then generate refined estimation within above rough initial clusters. In both steps, the numbers of clusters are selected by Calinski-Harabasz index using R package *NbClust* [36, 37], which is widely used for determining the number of components in various clustering methods. An adjustment is made based on prior information, and the initial values are denoted by $\left( \varvec{\beta }^{(0)},\varvec{\gamma }^{(0)}\right)$. Moreover, the other initial parameters are set as $\left( \varvec{\omega }_{jm}^{(0)},\varvec{\eta }_{jm}^{(0)}\right) =\left( \varvec{\beta }_{j}^{(0)}-\varvec{\beta }_{m}^{(0)},\varvec{\gamma }_{j}^{(0)}-\varvec{\gamma }_{m}^{(0)}\right)$, and $\left( \varvec{v}^{(0)},\varvec{u}^{(0)}\right) =\left( \varvec{0},\varvec{0}\right)$. Given $\left( \varvec{\beta }^{(t)},\varvec{\gamma }^{(t)},\varvec{\omega }^{(t)},\varvec{\eta }^{(t)},\varvec{v}^{(t)},\varvec{u}^{(t)}\right)$ at the begin of $(t+1)$-th iteration, the $(t+1)$-th iteration goes as follows,

4.1 $$\begin{aligned}{} & {} \left( \varvec{\beta }^{(t+1)},\varvec{\gamma }^{(t+1)}\right) =\arg \min _{\varvec{\beta }\in \mathcal {M}_{1},\varvec{\gamma }\in \mathcal {M}_{2}} \mathcal {L}\left( \varvec{\beta },\varvec{\gamma },\varvec{\omega }^{(t)},\varvec{\eta }^{(t)},\varvec{v}^{(t)},\varvec{u}^{(t)}\right) , \end{aligned}$$

4.2 $$\begin{aligned}{} & {} \quad \left( \varvec{\omega }^{(t+1)},\varvec{\eta }^{(t+1)}\right) =\arg \min _{\varvec{\omega },\varvec{\eta }} \mathcal {L}\left( \varvec{\beta }^{(t+1)},\varvec{\gamma }^{(t+1)},\varvec{\omega },\varvec{\eta },\varvec{v}^{(t)},\varvec{u}^{(t)}\right) , \end{aligned}$$

4.3 $$\begin{aligned}{} & {} \quad \varvec{v}_{jm}^{(t+1)}=\varvec{v}_{jm}^{(t)}+\vartheta \left( \varvec{\beta }_{j}^{(t+1)}-\varvec{\beta }_{m}^{(t+1)}-\varvec{\omega }_{jm}^{(t+1)}\right) ,\nonumber \\{} & {} \quad \varvec{u}_{jm}^{(t+1)}=\varvec{u}_{jm}^{(t)}+\vartheta \left( \varvec{\gamma }_{j}^{(t+1)}-\varvec{\gamma }_{m}^{(t+1)}-\varvec{\eta }_{jm}^{(t+1)}\right) . \end{aligned}$$

To obtain the solutions of the above optimization problems, we introduce some new notations. Recall the clustering structure $\left\{ \mathcal {F}_{1},\ldots ,\mathcal {F}_{K}\right\}$ transformed by prior information in section “Access to prior information”, we define a $n\times K$ matrix $\varvec{L}$ with $l_{ik}=1$ for $i\in \mathcal {F}_{k}$ and $l_{ik}=0$. Define $\varvec{J}\triangleq \varvec{L}\otimes \varvec{I}_{q+p}$, where $\otimes$ is Kronecker product and $\varvec{I}_{q+p}$ is $(q+p)\times (q+p)$ identity matrix. Here, $\varvec{J}$ is a $n(q+p)\times K(q+p)$ matrix. Define matrix $\varvec{D}=\left\{ \varvec{e}_{j}-\varvec{e}_{m}, \left( j,m\right) \in \mathcal {A}\right\} ^{\textrm{T}}$ with $\varvec{e}_{i}$ being a $n\times 1$ vector whose *i*-th element is 1 and the remaining ones are 0, and $\varvec{H}\triangleq \varvec{D}\otimes \varvec{I}_{q+p}$. Here, $\varvec{D}$ is a $\frac{1}{2}n(n-1)\times n$ matrix and $\varvec{H}$ is a $\frac{1}{2}n(n-1)(q+p)\times n(q+p)$ matrix.

Given $\left( \varvec{\omega }^{(t)},\varvec{\eta }^{(t)},\varvec{v}^{(t)},\varvec{u}^{(t)}\right)$, to obtain the solution of (4.1), denote $\varvec{X}=\left( \varvec{X}_{1},\ldots ,\varvec{X}_{n}\right)$ and $\varvec{Z}=\left( \varvec{Z}_{1},\ldots ,\varvec{Z}_{n}\right)$ as the $q\times n$ and $p\times n$ matrices, respectively. Define $\textrm{vec}\left( \cdot \right)$ as the vectorization of matrices. Then, the updates for $\left( \varvec{\beta }^{(t+1)},\varvec{\gamma }^{(t+1)}\right)$ are

4.4 $$\begin{aligned}&\textrm{vec}\left( \left( \varvec{\beta }^{(t+1)\textrm{T}},\varvec{\gamma }^{(t+1)\textrm{T}}\right) ^{\textrm{T}}\right) \\ =&\varvec{J}\textrm{vec}\left( \left\{ \left( \varvec{X}^{\textrm{T}},\varvec{Z}^{\textrm{T}}\right) ^{\textrm{T}}+\left( \vartheta \varvec{\omega }^{(t)\textrm{T}}-\varvec{v}^{(t)\textrm{T}},\vartheta \varvec{\eta }^{(t)\textrm{T}}-\varvec{u}^{(t)\textrm{T}}\right) ^{\textrm{T}}\varvec{D}\right\} \right. \\&\left. \varvec{L}\left( \varvec{L}^{\textrm{T}}\varvec{L}+\vartheta \varvec{L}^{\textrm{T}}\varvec{D}^{\textrm{T}}\varvec{D}\varvec{L}\right) ^{-1}\right) \end{aligned}$$

In particular, by some linear algebra techniques, we avoid calculating the inverse of a $K(q+p)\times K(q+p)$ matrix in the iterations, namely, $\varvec{J}^{\textrm{T}}\varvec{J}+\vartheta \varvec{J}^{\textrm{T}}\varvec{H}^{\textrm{T}}\varvec{H}\varvec{J}$. Instead, we calculate the inverse of a $K\times K$ matrix $\varvec{L}^{\textrm{T}}\varvec{L}+\vartheta \varvec{L}^{\textrm{T}}\varvec{D}^{\textrm{T}}\varvec{D}\varvec{L}$, which significantly reduces computation time. It is also noted that *K* is smaller than sample size *n* to some extent depending on the prior information. Especially, $K=n$ in the case of no prior information is also included in above formula, leading to a analytical inverse of a $n\times n$ matrix $\varvec{I}_{n}+\vartheta \varvec{D}^{\textrm{T}}\varvec{D}$. The detailed deviation of (4.4) is available in Additional file 1.

Given $\left( \varvec{\beta }^{(t+1)},\varvec{\gamma }^{(t+1)},\varvec{v}^{(t)},\varvec{u}^{(t)}\right)$, to obtain the solution of (4.2), let $\varvec{\omega }_{jm}^{*(t)}=\varvec{\beta }_{j}^{(t+1)}-\varvec{\beta }_{m}^{(t+1)}+\vartheta ^{-1}\varvec{v}_{jm}^{(t)}$ and $\varvec{\eta }_{jm}^{*(t)}=\varvec{\gamma }_{j}^{(t+1)}-\varvec{\gamma }_{m}^{(t+1)}+\vartheta ^{-1}\varvec{u}_{jm}^{(t)}$. Denote $\varvec{\omega }^{*(t)}=\left\{ \varvec{\omega }_{jm}^{*(t)}, \left( j,m\right) \in \mathcal {A}\right\}$ and $\varvec{\eta }^{*(t)}=\left\{ \varvec{\eta }_{jm}^{*(t)}, \left( j,m\right) \in \mathcal {A}\right\}$. Then, the updates for $\left( \varvec{\omega }_{jm}^{(t+1)},\varvec{\eta }_{jm}^{(t+1)}\right)$ are

4.5 $$\begin{aligned} \left( \varvec{\omega }_{jm}^{(t+1)},\varvec{\eta }_{jm}^{(t+1)}\right) =S\left( \varvec{\omega }_{jm}^{*(t)},\varvec{\eta }_{jm}^{*(t)}\right) , \end{aligned}$$

where $S\left( \varvec{\omega }_{jm}^{*(t)},\varvec{\eta }_{jm}^{*(t)}\right)$ is hierarchical groupwise thresholding operator provided in Additional file 1. Given $\left( \varvec{\beta }^{(t+1)},\varvec{\gamma }^{(t+1)},\varvec{\omega }^{(t+1)},\varvec{\eta }^{(t+1)},\varvec{v}^{(t)},\varvec{u}^{(t)}\right)$, the updates $\left( \varvec{v}^{(t+1)},\varvec{u}^{(t+1)}\right)$ follow (4.3). These updates are repeated until convergence.

We select the tuning parameters by minimizing the modified Bayesian information criterion (BIC). In this paper, similar that of [31], but in order to focus on the results of clustering, $\left( \lambda _{1},\lambda _{2}\right)$ are chosen by minimizing the following modified BIC-type criterion via a grid search,

$$\begin{aligned} \textrm{BIC}\left( \lambda _{1},\lambda _{2}\right) =&\log \left\{ \frac{1}{n}\sum _{i=1}^{n}\left( \left\| \varvec{X}_{i}-\widehat{\varvec{\beta }}_{i}\left( \lambda _{1},\lambda _{2}\right) \right\| _{2}^{2}+\left\| \varvec{Z}_{i}-\widehat{\varvec{\gamma }}_{i}\left( \lambda _{1},\lambda _{2}\right) \right\| _{2}^{2}\right) \right\} \\&+C_{n}\frac{\log (n)}{n}\left( \widehat{K}_{1}\left( \lambda _{1},\lambda _{2}\right) +\widehat{K}_{2}\left( \lambda _{1},\lambda _{2}\right) \right) , \end{aligned}$$

where $C_{n}$ is a positive value depending on *n*. In our implementation, we choose $C_{n}=\log \left( \log (n)\right)$, and note that $C_{n}=1$ (original BIC) and $C_{n}=\log (n)$ are also applicable.

## Simulation studies

In this section, we mainly consider two different scenarios in our simulation studies, Gaussian clusters and half-moon clusters, both of which demonstrate the superior performance of our proposed method to alternatives. Consider *n* independent data observations with $(q+p)$-dimensional features, which belong to $K_{1}$ rough clusters and $K_{2}$ refined clusters. The refined clusters label $Y_{i}$ of the *i*-th subject is uniformly sampled from $\{1,\ldots ,K_{2}\}$, and the $K_{2}$ refined clusters are nested in $K_{1}$ rough clusters as the discussion above. Under the refined clustering structure $\left\{ \mathcal {T}_{1}^{*},\ldots ,\mathcal {T}_{K_{2}}^{*}\right\}$, there are $N_{0}=\frac{1}{2}\sum _{k_{2}=1}^{K_{2}}\left| \mathcal {T}_{k_{2}}^{*}\right| \left( \left| \mathcal {T}_{k_{2}}^{*}\right| -1\right)$ true pairwise subject indexes which indicate all pairwise subjects belonging in corresponding same clusters. We randomly select $\left[ \tau N_{0} \right]$ pairwise indexes of true pairwise subject indexes to generate prior information $\mathcal {A}^{\textrm{p}}$, where $\tau$ controls how many pairwise relationships we select as known prior information for analysis, and $\left[ \tau N_{0} \right]$ is the greatest integer that is less than or equal to $\tau N_{0}$. In our simulation, we set $n=120$, $q=6$, $p=30$. To gain a clear sight on how prior improves clustering on hierarchical heterogeneous data, we consider two levels of prior information with $\tau _{1}=4\%$ (Prior1) and $\tau _{2}=8\%$ (Prior2), and also adopt “no prior” case as a baseline.

To demonstrate the competitive performance of our proposed methods (denoted by PCH-NoPrior, PCH-Prior1, and PCH-Prior2), we consider some alternatives for comparison. To the best of our knowledge, there are no existing clustering methods producing a hierarchical structure in one step estimation. As mentioned before, we employ a two-step estimation method combing with convex clustering methods. In brief, we conduct a first convex clustering on all subjects with the first type of features and obtain the estimated rough clustering structure, and then apply the second convex clustering on subjects with the second type of features belonging to each rough cluster respectively. The convex clustering procedures of two steps can be directly implemented using R package *cvxclustr*, and two alternatives are (a) CvxClu-$L_{1}$, which is the above two-step convex clustering method with $L_{1}$-penalty, and (b) CvxClu-$L_{2}$, which is the above two-step convex clustering method with $L_{2}$-penalty. The identification of estimated rough and refined clustering structure by our proposed methods is described in section “Hierarchical penalties with prior information incorporated”, and the clustering results of alternatives are outputs by the two-step convex clustering procedure. With the above estimation, we adopt the following measures to assess performance. (1) Adjusted Rand Index (ARI) [38, 39], which is an indicator to compare the estimated rough and refined clustering structure with true situation. Denote TP/FP as the times of decision assigning two subjects from same/different ground truth cluster to same estimated cluster, and TN/FN as the times of decision assigning two subjects from different/same ground truth clusters to different estimated clusters, then ARI is defined by

$$\begin{aligned} \frac{2(\textrm{TP}\times \textrm{TN}-\textrm{FP}\times \textrm{FN})}{(\textrm{TP}+\textrm{FP})(\textrm{FP}+\textrm{TN})+(\textrm{TP}+\textrm{FN})(\textrm{FN}+\textrm{TN})}. \end{aligned}$$

Note that $\textrm{ARI}\in [-1,1]$. A higher value indicates better clustering performance, and a random clustering structure takes ARI close to 0. (2) Mean squared errors (MSEs) of $\widehat{\varvec{\beta }}$ and $\widehat{\varvec{\gamma }}$, defined by $\left( \frac{1}{nq}\sum _{i=1}^{n}\left\| \widehat{\varvec{\beta }}_{i}-\varvec{\beta }_{i}^{*}\right\| _{2}^{2}\right) ^{\frac{1}{2}}$ and $\left( \frac{1}{np}\sum _{i=1}^{n}\left\| \widehat{\varvec{\gamma }}_{i}-\varvec{\gamma }_{i}^{*}\right\| _{2}^{2}\right) ^{\frac{1}{2}}$, respectively. In the rest of this section, we illustrate the details of simulation settings under different scenarios, and generate 100 replicates for each setting.

### Gaussian clusters

Denote $\varvec{1}_{p}$ as the *p*-dimensional vector with all elements being 1. Denote $\textrm{MVN}_{p}$ as the *p*-dimensional multivariate normal distribution. For the *i*-th subject, two types of features are generated as follows, features $\varvec{X}_{i}\sim \textrm{MVN}_{q}\left( \varvec{\mu }_{\varvec{X}}\left( Y_{i}\right) ,\varvec{\Sigma }_{\varvec{X}}\right)$ and $\varvec{Z}_{i}\sim \textrm{MVN}_{p}\left( \varvec{\mu }_{\varvec{Z}}\left( Y_{i}\right) ,\varvec{\Sigma }_{\varvec{Z}}\right)$, where the mean $\varvec{\mu }_{\varvec{X}}\left( Y_{i}\right)$ and $\varvec{\mu }_{\varvec{Z}}\left( Y_{i}\right)$ are generated in Table 1. We consider $\mu _{1}=1.2$ and $\mu _{2}=1.6$, which control the distance between cluster centers and bring different levels of difficulty to clustering on hierarchical data. In each simulation, the covariance matrices $\varvec{\Sigma }_{\varvec{X}}=\sigma ^{2}\varvec{I}_{q}$ and $\varvec{\Sigma }_{\varvec{Z}}=\left( \sigma _{\varvec{Z}jm}\right) _{1\leqslant j,m\leqslant p}$ is generated under three cases. Specifically, the diagonal case with $\sigma _{\varvec{Z}jm}=\sigma ^{2}\mathbb {I}_{\left\{ j=m\right\} }$, the auto-regressive (AR) case with $\sigma _{\varvec{Z}jm}=\sigma ^{2}\mathbb {I}_{\left\{ j=m\right\} }+\sigma ^{2}\rho ^{\left| j-m\right| }\mathbb {I}_{\left\{ j\ne m\right\} }$, and the banded case with $\sigma _{\varvec{Z}jm}=\sigma ^{2}\mathbb {I}_{\left\{ j=m\right\} }+\sigma ^{2}\rho \mathbb {I}_{\left\{ \left| j-m\right| =1\right\} }$, where we fix $\sigma ^{2}=1$ and $\rho =0.3$.

**Table 1:** Three simulation settings under Gaussian clusters cases

	$\left( K_{1},K_{2}\right)$	Hierarchical Structure	$\varvec{\mu }_{\varvec{X}}\left( Y_{i}\right)$	$\varvec{\mu }_{\varvec{Z}}\left( Y_{i}\right)$
Simulation 1	(2, 4)	$\mathcal {G}_{1}^{*}=\left\{ \mathcal {T}_{1}^{*},\mathcal {T}_{2}^{*}\right\}$	$\frac{4}{5}\mu \left( -\varvec{1}_{\frac{q}{3}}^{\textrm{T}},\varvec{1}_{\frac{q}{3}}^{\textrm{T}},\varvec{1}_{\frac{q}{3}}^{\textrm{T}}\right) ^{\textrm{T}}, Y_{i}\in \{1,2\}$	$\mu \left( \varvec{1}_{\frac{p}{2}}^{\textrm{T}},\varvec{1}_{\frac{p}{2}}^{\textrm{T}}\right) ^{\textrm{T}}, Y_{i}=1$
				$$\mu \left( -\varvec{1}_{\frac{p}{2}}^{\textrm{T}},\varvec{1}_{\frac{p}{2}}^{\textrm{T}}\right) ^{\textrm{T}}, Y_{i}=2$$
		$\mathcal {G}_{2}^{*}=\left\{ \mathcal {T}_{3}^{*},\mathcal {T}_{4}^{*}\right\}$	$\frac{4}{5}\mu \left( \varvec{1}_{\frac{q}{3}}^{\textrm{T}},\varvec{1}_{\frac{q}{3}}^{\textrm{T}},-\varvec{1}_{\frac{q}{3}}^{\textrm{T}}\right) ^{\textrm{T}}, Y_{i}\in \{3,4\}$	$\mu \left( \varvec{1}_{\frac{p}{2}}^{\textrm{T}},-\varvec{1}_{\frac{p}{2}}^{\textrm{T}}\right) ^{\textrm{T}}, Y_{i}=3$
				$$\mu \left( -\varvec{1}_{\frac{p}{2}}^{\textrm{T}},-\varvec{1}_{\frac{p}{2}}^{\textrm{T}}\right) ^{\textrm{T}}, Y_{i}=4$$
Simulation 2	(2, 6)	$\mathcal {G}_{1}^{*}=\left\{ \mathcal {T}_{1}^{*},\mathcal {T}_{2}^{*},\mathcal {T}_{3}^{*}\right\}$	$\frac{4}{5}\mu \left( -\varvec{1}_{\frac{q}{3}}^{\textrm{T}},\varvec{1}_{\frac{q}{3}}^{\textrm{T}},\varvec{1}_{\frac{q}{3}}^{\textrm{T}}\right) ^{\textrm{T}}, Y_{i}\in \{1,2,3\}$	$\mu \left( -\varvec{1}_{\frac{p}{3}}^{\textrm{T}},\varvec{1}_{\frac{p}{3}}^{\textrm{T}},\varvec{1}_{\frac{p}{3}}^{\textrm{T}}\right) ^{\textrm{T}}, Y_{i}=1$
				$$\mu \left( \varvec{1}_{\frac{p}{3}}^{\textrm{T}},-\varvec{1}_{\frac{p}{3}}^{\textrm{T}},\varvec{1}_{\frac{p}{3}}^{\textrm{T}}\right) ^{\textrm{T}}, Y_{i}=2$$
				$$\mu \left( \varvec{1}_{\frac{p}{3}}^{\textrm{T}},\varvec{1}_{\frac{p}{3}}^{\textrm{T}},-\varvec{1}_{\frac{p}{3}}^{\textrm{T}}\right) ^{\textrm{T}}, Y_{i}=3$$
		$\mathcal {G}_{2}^{*}=\left\{ \mathcal {T}_{4}^{*},\mathcal {T}_{5}^{*},\mathcal {T}_{6}^{*}\right\}$	$\frac{4}{5}\mu \left( \varvec{1}_{\frac{q}{3}}^{\textrm{T}},\varvec{1}_{\frac{q}{3}}^{\textrm{T}},-\varvec{1}_{\frac{q}{3}}^{\textrm{T}}\right) ^{\textrm{T}}, Y_{i}\in \{4,5,6\}$	$\mu \left( \varvec{1}_{\frac{p}{3}}^{\textrm{T}},-\varvec{1}_{\frac{p}{3}}^{\textrm{T}},-\varvec{1}_{\frac{p}{3}}^{\textrm{T}}\right) ^{\textrm{T}}, Y_{i}=4$
				$$\mu \left( -\varvec{1}_{\frac{p}{3}}^{\textrm{T}},\varvec{1}_{\frac{p}{3}}^{\textrm{T}},-\varvec{1}_{\frac{p}{3}}^{\textrm{T}}\right) ^{\textrm{T}}, Y_{i}=5$$
				$$\mu \left( -\varvec{1}_{\frac{p}{3}}^{\textrm{T}},-\varvec{1}_{\frac{p}{3}}^{\textrm{T}},\varvec{1}_{\frac{p}{3}}^{\textrm{T}}\right) ^{\textrm{T}}, Y_{i}=6$$
Simulation 3	(3, 6)	$\mathcal {G}_{1}^{*}=\left\{ \mathcal {T}_{1}^{*},\mathcal {T}_{2}^{*}\right\}$	$\frac{4}{5}\mu \left( -\varvec{1}_{\frac{q}{3}}^{\textrm{T}},\varvec{1}_{\frac{q}{3}}^{\textrm{T}},\varvec{1}_{\frac{q}{3}}^{\textrm{T}}\right) ^{\textrm{T}}, Y_{i}\in \{1,2\}$	$\mu \left( -\varvec{1}_{\frac{p}{3}}^{\textrm{T}},\varvec{1}_{\frac{p}{3}}^{\textrm{T}},\varvec{1}_{\frac{p}{3}}^{\textrm{T}}\right) ^{\textrm{T}}, Y_{i}=1$
				$$\mu \left( \varvec{1}_{\frac{p}{3}}^{\textrm{T}},-\varvec{1}_{\frac{p}{3}}^{\textrm{T}},-\varvec{1}_{\frac{p}{3}}^{\textrm{T}}\right) ^{\textrm{T}}, Y_{i}=2$$
		$\mathcal {G}_{2}^{*}=\left\{ \mathcal {T}_{3}^{*},\mathcal {T}_{4}^{*}\right\}$	$\frac{4}{5}\mu \left( \varvec{1}_{\frac{q}{3}}^{\textrm{T}},-\varvec{1}_{\frac{q}{3}}^{\textrm{T}},\varvec{1}_{\frac{q}{3}}^{\textrm{T}}\right) ^{\textrm{T}},Y_{i}\in \{3,4\}$	$\mu \left( \varvec{1}_{\frac{p}{3}}^{\textrm{T}},-\varvec{1}_{\frac{p}{3}}^{\textrm{T}},\varvec{1}_{\frac{p}{3}}^{\textrm{T}}\right) ^{\textrm{T}}, Y_{i}=3$
				$$\mu \left( -\varvec{1}_{\frac{p}{3}}^{\textrm{T}},\varvec{1}_{\frac{p}{3}}^{\textrm{T}},-\varvec{1}_{\frac{p}{3}}^{\textrm{T}}\right) ^{\textrm{T}}, Y_{i}=4$$
		$\mathcal {G}_{3}^{*}=\left\{ \mathcal {T}_{5}^{*},\mathcal {T}_{6}^{*}\right\}$	$\frac{4}{5}\mu \left( \varvec{1}_{\frac{q}{3}}^{\textrm{T}},\varvec{1}_{\frac{q}{3}}^{\textrm{T}},-\varvec{1}_{\frac{q}{3}}^{\textrm{T}}\right) ^{\textrm{T}},Y_{i}\in \{5,6\}$	$\mu \left( \varvec{1}_{\frac{p}{3}}^{\textrm{T}},\varvec{1}_{\frac{p}{3}}^{\textrm{T}},-\varvec{1}_{\frac{p}{3}}^{\textrm{T}}\right) ^{\textrm{T}}, Y_{i}=5$
				$$\mu \left( -\varvec{1}_{\frac{p}{3}}^{\textrm{T}},-\varvec{1}_{\frac{p}{3}}^{\textrm{T}},\varvec{1}_{\frac{p}{3}}^{\textrm{T}}\right) ^{\textrm{T}}, Y_{i}=6$$

**Fig. 1:**
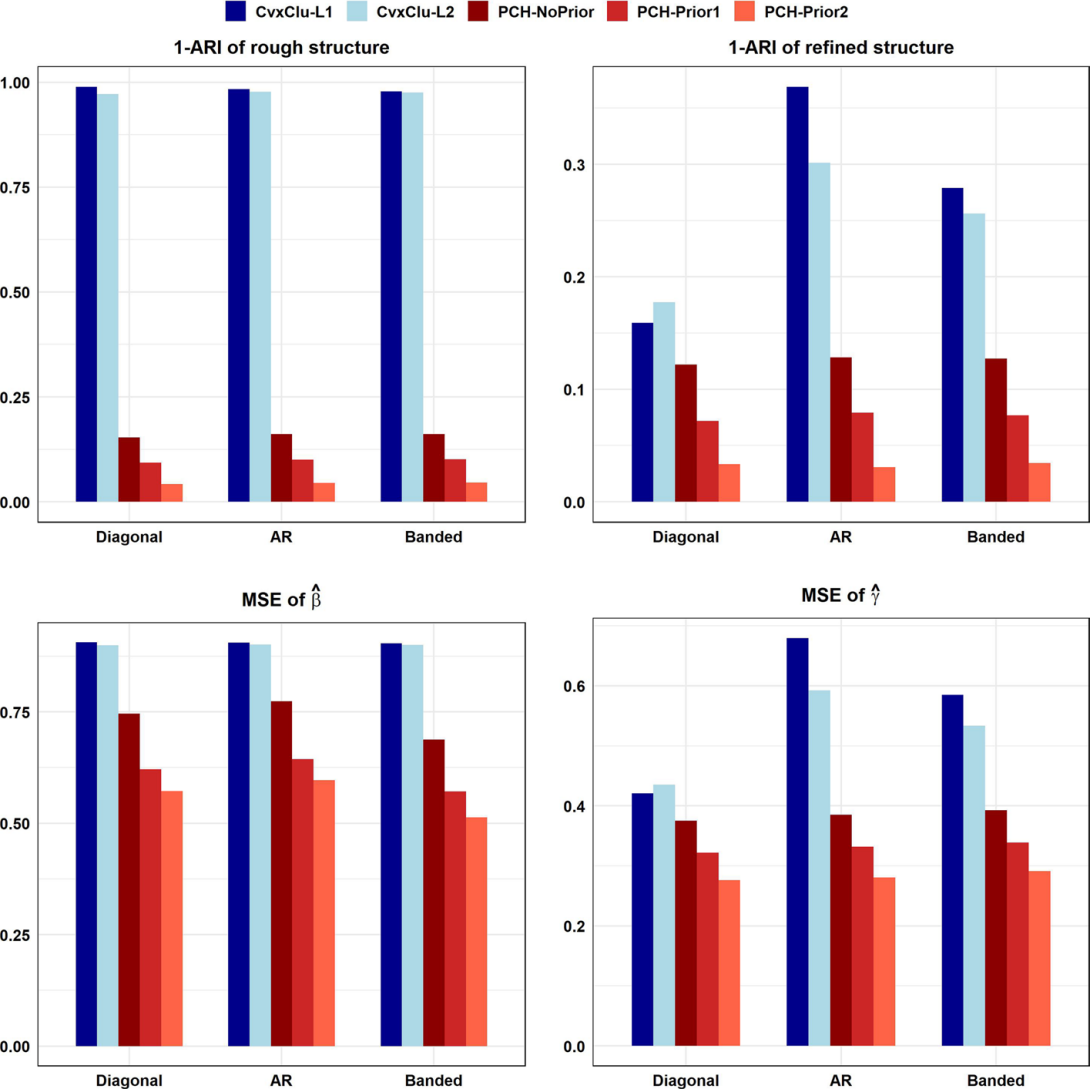
Simulation results with Simulation 3 and $\mu _{1}=1.2$. In each subfigure, horizontal axis displays our proposed methods and alternatives with three different covariance matrices, and longitudinal axis displays the mean of corresponding measurement values under 100 simulated replicates. The top-left subfigure displays $1-\textrm{ARI}$ of rough clustering structure, the top-right subfigure displays $1-\textrm{ARI}$ of refined clustering structure, the bottom-left subfigure displays MSE of $\hat{\varvec{\beta }}$, and the bottom-right subfigure displays MSE of $\hat{\varvec{\gamma }}$

Our simulation results are summarized in Table 2 and Additional file 1: Tables S1–S3 and S5, and also visualized in Fig. 1 and Additional file 1: Figures S1–S5. Throughout the whole simulations, our proposed method shows highly competitive performance. Additional file 1: Table S5 shows the mean of number of estimated clusters under all Gaussian clusters cases. It is observed that our proposed methods maintain a mean value close to the true value, validating high recovery of true number of clusters. Then take Simulation 3 as an example. Table 2 displays the results for different levels of distance between cluster centers with Simulation 3 and AR covariance structure. Compare to the alternatives, our proposed method without any prior has higher ARIs of both rough and refined clustering structure, and lower MSEs, demonstrating our superior accuracy on clustering and parameter estimation despite the absence of prior information. In addition, with the assistance of valuable prior information, our proposed method achieves further significant improvement, and is progressively strengthened with more prior information incorporation. Although all methods seems to perform well when the distance between cluster centers $\mu _{2}=1.6$, the differences in various methods become apparent when the smaller distance between cluster centers makes clustering more difficult. Specifically, as shown in Table 2, in the level of $\mu _{1}=1.2$, the mean of ARIs of the estimated refined clustering structure are 0.6312 (CvxClu-$L_{1}$), 0.6986 (CvxClu-$L_{2}$), 0.8716 (PCH-NoPrior), 0.9209 (PCH-Prior1), and 0.9691 (PCH-Prior2), and even the alternatives fail in determining rough clustering structure. Fig. 1 further displays the results for different settings of covariance structures with Simulation 3 and $\mu _{1}=1.2$. The results on diagonal and banded structure are observed to follow a similar trend as those on AR structure. In a whole, under the Gaussian clusters case, our proposed method has shown remarkable performance on recovery of hierarchical clustering structure and estimation of cluster centers, particularly when combining with the limited but beneficial prior information.

**Table 2:** Simulation results with $\left( K_{1},K_{2}\right) =(3,6)$ and AR covariance structure in Gaussian clusters case. Simulation results include the mean and standard deviation (SD) of RI of rough and refined clustering structure, MSE of $\hat{\varvec{\beta }}$, and MSE of $\hat{\varvec{\gamma }}$ under 100 simulated replicates with $\mu _{1}=1.2$ and $\mu _{2}=1.6$

		Rough structure				Refined structure			
		ARI		MSE of $\hat{\varvec{\beta }}$		ARI		MSE of $\hat{\varvec{\gamma }}$	
	Methods	Mean	SD	Mean	SD	Mean	SD	Mean	SD
$\mu _{1}=1.2$	CvxClu-$L_{1}$	0.0167	0.0945	0.9052	0.0147	0.6312	0.1845	0.6794	0.1828
	CvxClu-$L_{2}$	0.0223	0.1089	0.9014	0.0337	0.6986	0.1455	0.5923	0.1350
	PCH-NoPrior	0.8390	0.0650	0.7736	0.5258	0.8716	0.0505	0.3854	0.0547
	PCH-Prior1	0.8998	0.0587	0.6436	0.4541	0.9209	0.0439	0.3321	0.0518
	PCH-Prior2	0.9549	0.0697	0.5963	0.5105	0.9691	0.0358	0.2803	0.0517
$\mu _{2}=1.6$	CvxClu-$L_{1}$	0.7697	0.2923	0.7092	0.3111	0.9032	0.0360	0.3416	0.0446
	CvxClu-$L_{2}$	0.8952	0.1742	0.5116	0.2348	0.8632	0.0455	0.3929	0.0498
	PCH-NoPrior	0.9660	0.0357	0.8460	0.8235	0.9728	0.0292	0.2833	0.0534
	PCH-Prior1	0.9760	0.0437	0.8073	0.8379	0.9835	0.0198	0.2562	0.0413
	PCH-Prior2	0.9915	0.0144	0.7837	0.8417	0.9930	0.0114	0.2386	0.0289

### Half-moon clusters

We consider a non-spherical case with two half-moon clusters. For each subject in the first rough cluster, the first two dimension features of $\varvec{X}$ are generated by a half-moon cluster with radius 4 and centers $\left( -\nu ,0.2\right)$, and the last $q-2$ dimension features of $\varvec{X}$ are generated by $\textrm{MVN}_{q-2}\left( \frac{4}{5}\mu \left( -1,-1,1, 1\right) ^{\textrm{T}},\varvec{I}_{q-2}\right)$. For each subject in the second rough cluster, the first two dimension features of $\varvec{X}$ are generated by a half-moon cluster with radius 4 and centers $\left( \nu ,-0.2\right)$, and the last $q-2$ dimension features of $\varvec{X}$ are generated by $\textrm{MVN}_{q-2}\left( \frac{4}{5}\mu \left( 1,1,-1,-1\right) ^{\textrm{T}},\varvec{I}_{q-2}\right)$. We add Gaussian random noise with mean zero and standard deviation 0.1 to each subject for the first two dimension feature of $\varvec{X}$. For the *i*-th subject, features $\varvec{Z}_{i}\sim \textrm{MVN}_{p}\left( \varvec{\mu }_{\varvec{Z}}\left( Y_{i}\right) ,\varvec{\Sigma }_{\varvec{Z}}\right)$. We consider another two simulation settings under the case with two half-moon clusters. Simulations 4 and 5 consider that all settings are the same as Simulations 1 and 2 in Table 1 except feature $\varvec{X}$, respectively. Let $\varvec{\Sigma }_{\varvec{Z}}=\varvec{I}_{p}$ as the diagonal covariance structure and $\mu =1$. In each simulation, we consider the near and far centers of two half-moon clusters with $\nu _{1}=2$ and $\nu _{2}=3$.

**Fig. 2:**
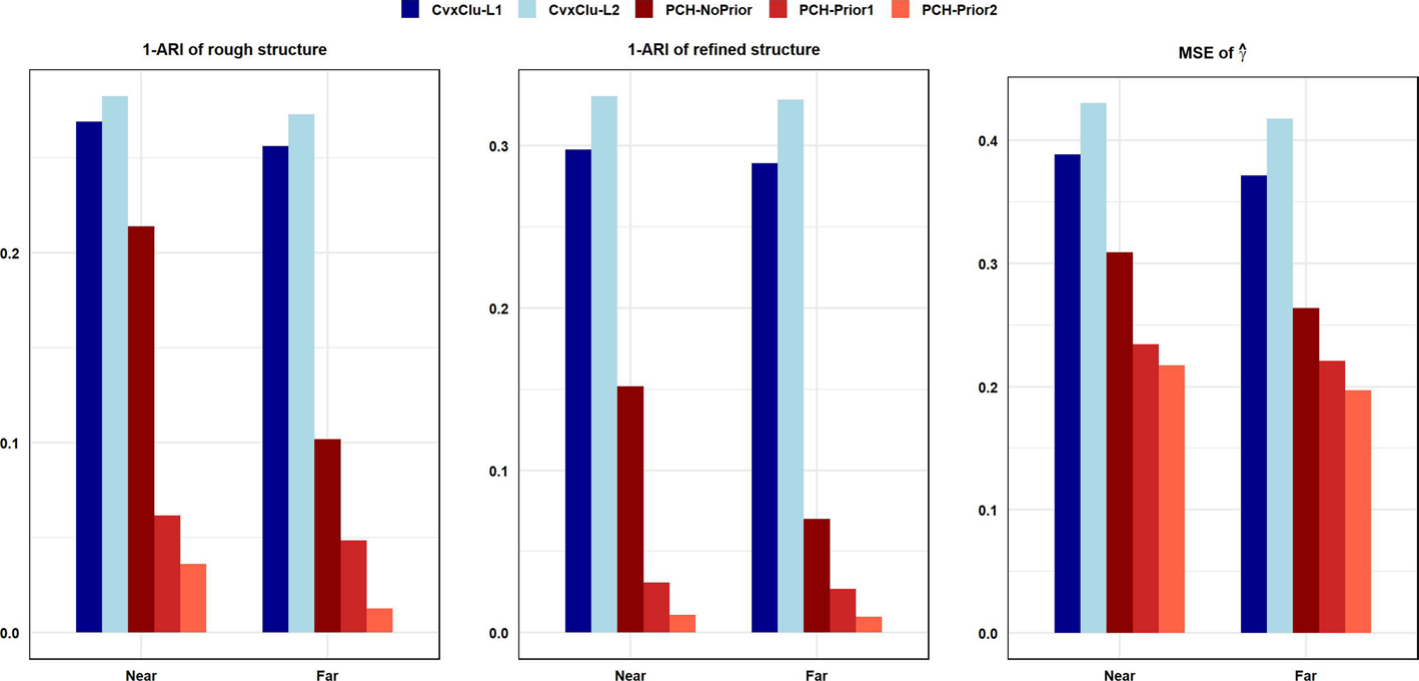
Simulation results with Simulation 4 in two half-moon clusters case. In each subfigure, horizontal axis displays our proposed methods and alternatives with near and far centers, and longitudinal axis displays the mean of corresponding measurement values under 100 simulated replicates. The left subfigure displays $1-\textrm{ARI}$ of rough clustering structure, the middle subfigure displays $1-\textrm{ARI}$ of refined clustering structure, and the right subfigure displays MSE of $\hat{\varvec{\gamma }}$

Our simulation results are summarized in Table 3 and Additional file 1: Tables S4–S5, and also visualized in Fig. 2 and Additional file 1: Figures S6. Here, we omit the MSE of $\widehat{\varvec{\beta }}$ since the first two dimension features of $\varvec{X}$ do not satisfy spherical structure. From Additional file 1: Table S5, it is observed that our proposed methods still maintain high recovery of true number of clusters. Table 3 and Fig. 2 demonstrate the results for different half-moon cluster centers with Simulation 4. It is evident that whether the half-moon centers are near or far, there is no essential difference in outcomes. Under such non-spherical case, our proposed method still holds advantages over the alternatives and achieves outstandingly precise estimation, which are further enhanced with additional prior information. This can be observed by the mean of ARIs of the estimated rough clustering structure with near centers in Table 3, which are 0.7309 (CvxClu-$L_{1}$), 0.7176 (CvxClu-$L_{2}$), 0.7862 (PCH-NoPrior), 0.9383 (PCH-Prior1), and 0.9637 (PCH-Prior2). This validates that our proposed method can perform effectively under diverse data distribution patterns.

**Table 3:** Simulation results with $\left( K_{1},K_{2}\right) =(2,4)$ in two half-moon clusters case. Simulation results include the mean and standard deviation (SD) of RI of rough and refined clustering structure, and MSE of $\hat{\varvec{\gamma }}$ under 100 simulated replicates with near and far centers

		Rough structure				Refined structure			
		ARI				ARI		MSE of $\hat{\varvec{\gamma }}$	
	Methods	Mean			SD	Mean	SD	Mean	SD
Near centers	CvxClu-$L_{1}$	0.7309			0.1689	0.7024	0.1596	0.3886	0.0907
	CvxClu-$L_{2}$	0.7176			0.1755	0.6696	0.1662	0.4303	0.0838
	PCH-NoPrior	0.7862			0.1623	0.8483	0.1299	0.3092	0.0949
	PCH-Prior1	0.9383			0.1412	0.9691	0.0336	0.2343	0.0787
	PCH-Prior2	0.9637			0.1431	0.9890	0.0195	0.2174	0.0774
Far centers	CvxClu-$L_{1}$	0.7438			0.1709	0.7108	0.1621	0.3714	0.0856
	CvxClu-$L_{2}$	0.7272			0.1694	0.6717	0.1653	0.4176	0.0827
	PCH-NoPrior	0.8981			0.1053	0.9300	0.0813	0.2639	0.0539
	PCH-Prior1	0.9514			0.1043	0.9730	0.0295	0.2208	0.0409
	PCH-Prior2	0.9872			0.0397	0.9903	0.0167	0.1969	0.0240

### Additional exploration

As described in section “Introduction”, combining analysis of hierarchical data may further improve the understanding of cancer and other complex diseases. Although hierarchy driven by data is biologically sensible and methodologically feasible, it is still interesting and insightful to explore how well the proposed methods and the alternatives perform under a scenario violating hierarchy. As the Additional file 1: Figure S8 suggests, we combine previous 3-rd and 4-th refined clusters to one refined cluster while retaining same rough clusters, thus generate 2 rough clusters and 5 refined clusters which violates hierarchy. The rough cluster centers are $\frac{4}{5}\mu \left( -\varvec{1}_{\frac{q}{3}}^{\textrm{T}},\varvec{1}_{\frac{q}{3}}^{\textrm{T}},\varvec{1}_{\frac{q}{3}}^{\textrm{T}}\right) ^{\textrm{T}}$ and $\frac{4}{5}\mu \left( \varvec{1}_{\frac{q}{3}}^{\textrm{T}},\varvec{1}_{\frac{q}{3}}^{\textrm{T}},-\varvec{1}_{\frac{q}{3}}^{\textrm{T}}\right) ^{\textrm{T}}$, while the refined cluster centers are $\mu \left( \varvec{1}_{\frac{p}{2}}^{\textrm{T}},\varvec{1}_{\frac{p}{2}}^{\textrm{T}}\right) ^{\textrm{T}}$, $\mu \left( -\varvec{1}_{\frac{p}{2}}^{\textrm{T}},\varvec{1}_{\frac{p}{2}}^{\textrm{T}}\right) ^{\textrm{T}}$,$\varvec{0}_{p}$, $\mu \left( \varvec{1}_{\frac{p}{2}}^{\textrm{T}},-\varvec{1}_{\frac{p}{2}}^{\textrm{T}}\right) ^{\textrm{T}}$, and $\mu \left( -\varvec{1}_{\frac{p}{2}}^{\textrm{T}},-\varvec{1}_{\frac{p}{2}}^{\textrm{T}}\right) ^{\textrm{T}}$. Other settings are the same as those described above. The results are summarized in Additional file 1: Table S6. As expected, throughout all simulation settings, the clustering performance is still acceptable. Under banded covariance structure and $\mu _{2}=1.6$ which makes clustering easier, the mean of ARIs of estimated rough/refined clusters are 0.8151/0.7337 (CvxClu-$L_{1}$), 0.9648/0.6938 (CvxClu-$L_{2}$), 0.9804/0.7397 (PCH-NoPrior), 0.9856/0.7407 (PCH-Prior1), and 0.9930/0.7445 (PCH-Prior2), showing that our proposed methods perform well and clustering performance is superior to alternatives. Under banded covariance structure and $\mu _{1}=1.2$ which makes clustering difficult, our proposed methods are still acceptable while alternatives all fail in ARI.

We also note that prior information is not fully corrected all the times, and the influences on clustering results with partly wrong prior pairwise relationships deserve exploration. Therefore, we conduct additional simulations to find out how sensitive mis-specified prior on the final clustering results. Recall Simulation 3 in Table 1, in each simulated data, we add mis-specified information into the previous generated prior, denoted by PCH-misPrior1 and PCH-misPrior2. Specifically, with prior clustering structure $\left\{ \mathcal {F}_{1},\ldots ,\mathcal {F}_{K}\right\}$ transformed by previous prior pairwise relationships $\mathcal {A}^{\textrm{p}}$, we combine the first ten prior clusters with the largest sample size and ten prior clusters with the smallest sample size, respectively, which accounts for lots of wrong pairwise relationships. The results are summarized in Additional file 1: Table S7. Take banded covariance structure and $\mu _{1}=1.2$ as an example, the mean of ARIs of estimated rough/refined clustering structure are 0.0217/0.7209 (CvxClu-L1), 0.0243/0.7439 (CvxClu-L2), 0.8388/0.8726 (PCH-NoPrior), 0.8984/0.9230 (PCH-Prior1), 0.9538/0.9657 (PCH-Prior2), 0.7079/0.7669 (PCH-misPrior1), and 0.7889/0.8227 (PCH-misPrior2). Compared to fully correct information, mis-specified information does have impact on the clustering performance to some extent, but the ARIs (all larger than 0.7) are still acceptable. Our proposed methods are not too sensitive with incorrect prior and still superior to alternatives with higher ARIs.

Our proposed methods can be also directly applied to high dimensional scenarios. We adjust parameters as $p=120$, where *p* is equal to *n*, and other settings are the same as those described above. All results with $\mu _{1}=1.2$ are summarized in Additional file 1: Table S8. It can be clearly seen that our proposed methods still have competitive estimation performance, even if alternatives may identify a random rough clustering structure. But in high dimensional settings, the computational expenses will increase significantly, presented by Additional file 1: Table S9. Roughly speaking, for analyzing one replicate by proposed framework, it takes about 5 min on a laptop with regular configurations. But in high dimensional setting with $p=120$, the computational time of our proposed framework costs double as those with $p=30$, while the computational time of alternatives costs about ten times as those with $p=30$. In addition, we should particularly point out that sparsity often coincides with high dimensional scenarios, hence the feature selection is needed when *p* is large. We recognize that our framework is not currently applicable to this scenario with sparsity, since we focus on hierarchical heterogeneous structure and prior pairwise relationships. Inspired by sparse clustering framework [11, 34], our proposed methods can be modified with an additional spare group penalty to adapt high dimensional scenarios, and we will conduct further research in this area.

## Real data analysis

The Cancer Genome Atlas (TCGA), organized by the National Cancer Institute (NCI) and the National Human Genome Research Institute (NHGRI), is a comprehensive resource that provides a wealth of genomic and clinical data on various cancer types. Researchers use TCGA data due to its exceptional quality, user-friendly accessibility, and profound scientific influence. One of the extensively studied cancer types within TCGA is lung adenocarcinoma (LUAD), which is a heterogeneous subtype of non-small cell lung cancer and accounts for a significant portion of lung cancer cases. In this section, we analyze the clinical imaging data and the omics data on LUAD, and all analyzed data are publicly available at the TCGA data portal (https://portal.gdc.cancer.gov/projects/TCGA-LUAD). Note that recent studies have thoroughly conduct lung cancer heterogeneity using imaging data, yielding novel insights into disease biology and prognosis [40]. Similarly, heterogeneity analyses on omics data have also led to impactful biomedical discoveries, furthermore, it is evident that omics data-based analyses often complement rather than replace clinical imaging and other data [26].

In this study, the pipeline for extracting imaging features has been implemented in recent studies and briefly summarized in Additional file 1: Figure S9. One can refer to [41] and [42] for more detailed information on each step of process and quality control. For omics data, we focus our attention on mRNA gene expressions (over 20,000 gene features). Considering the limitation of sample size and burden of estimation efficiency, we adopt some dimension reduction techniques to enhance estimation reliability although the method is applicable to high-dimensional data. Specifically, we firstly use prescreening technique to remove meaningless genes, then concentrate on genes within the non-small cell lung cancer pathway (entry *hsa05223* in KEGG). Then we use principal component analysis (PCA) to extract principal components, the first 20 components contributing majority of the variance are included for further analysis. We obtain clinical imaging features and gene expression features measured for 355 patients, and only a small amount of them have additional helpful biomarkers sourced from the extensive and powerful TCGA project. The four collected biological indicators (FEV1 pre bronchodilator, FEV1 post bronchodilator, FEV1/FVC pre bronchodilator, and FEV1/FVC post bronchodilator) are crucial pulmonary function measurements in the clinical management of LUAD, important for assessing lung function, and vital tools in both diagnosis and treatment evaluation [43]. Since these measurements are shown to be helpful for clinically staging, we use traditional convex clustering method to extract useful prior clustering structure based on only a small amount of patients who have records on such four biomarkers. Then 123 prior pairwise relationships of 51 patients are transformed by the above prior clustering structure. Overall, the final analyzed data contains 6 extracted clinical imaging features and 20 principal component features based on omics data measured for 355 patients, and 51 patients among them have 123 pairwise subject indexes as prior information.

We use the proposed method to conduct LUAD data analysis. The initial values generation and tuning parameters criterion are consistent with those in section “Computational algorithm”. In our analysis, two rough clusters are identified, with sizes 203 and 152, respectively. Moreover, four distinct refined clusters are identified, with sizes 158, 45, 118, and 34, respectively, indicating that the two rough clusters are both split into two refined clusters. Detailed clustering information and the cluster centers estimation are available in Additional file 1: Table S10, suggesting that our proposed method is well-applicable and the clustering analysis is rational. It is observed that the four clusters have significantly different centers, which also validates the necessity to perform refined hierarchical analysis for subjects. We also apply the convex clustering methods to analyze LUAD data, which group lots of subjects into individual clusters respectively.

To further explore the clustering results and the biological significance of their hierarchical structure, we compare two extra clinical characteristics of the patients across the different clusters. We adopt disease free months (DFM) since initial treatment and overall survival in months (OSM) since initial diagnosis, sourced from TCGA project. These two metrics offer insights into treatment effectiveness and potential recurrence, and provide a comprehensive assessment of patients’ prognoses and the impact of treatments on their survival. ANOVA is employed to assess variations in the two metrics across estimated patient clusters. For DFM, the suggested *P*-values are 0.0558 across rough clusters and 0.0077 across refined clusters, respectively. For OSM, the suggested *P*-values are 0.0169 across rough clusters and 0.0044 across refined clusters, respectively. All *P*-values suggest significant difference among the estimated clustering structure. Notably, *P*-values ($<0.01$) across refined clusters are remarkably smaller than those across rough clusters, validating that refined clustering produces a more precise outcome with enhanced difference among clusters. It is especially noted that DFM and OSM are not included in the aforementioned heterogeneity analyses, as a result, there is no concern about over-fitting. In brief, this analysis contributes to the validity of the estimated hierarchical heterogeneous structure.

**Table 4:** Analysis of LUAD data. The results of mean and standard deviation (SD) of rough and refined clustering similarities by different methods under 100 replicates with $m=5,10,15$ subjects removed

		Rough Structure Similarity		Refined Structure Similarity	
	Methods	Mean	SD	Mean	SD
5 subjects removed	CvxClu-$L_{1}$	0.7322	0.0332	0.6427	0.0288
	CvxClu-$L_{2}$	0.6223	0.0136	0.6631	0.0075
	PCH	0.9159	0.0748	0.8005	0.0641
10 subjects removed	CvxClu-$L_{1}$	0.7159	0.0562	0.6335	0.0621
	CvxClu-$L_{2}$	0.6262	0.0214	0.6628	0.0099
	PCH	0.8878	0.0788	0.7776	0.0714
15 subjects removed	CvxClu-$L_{1}$	0.7115	0.0553	0.6320	0.0592
	CvxClu-$L_{2}$	0.6376	0.0295	0.6647	0.0137
	PCH	0.9382	0.0457	0.8025	0.0398

**Fig. 3:**
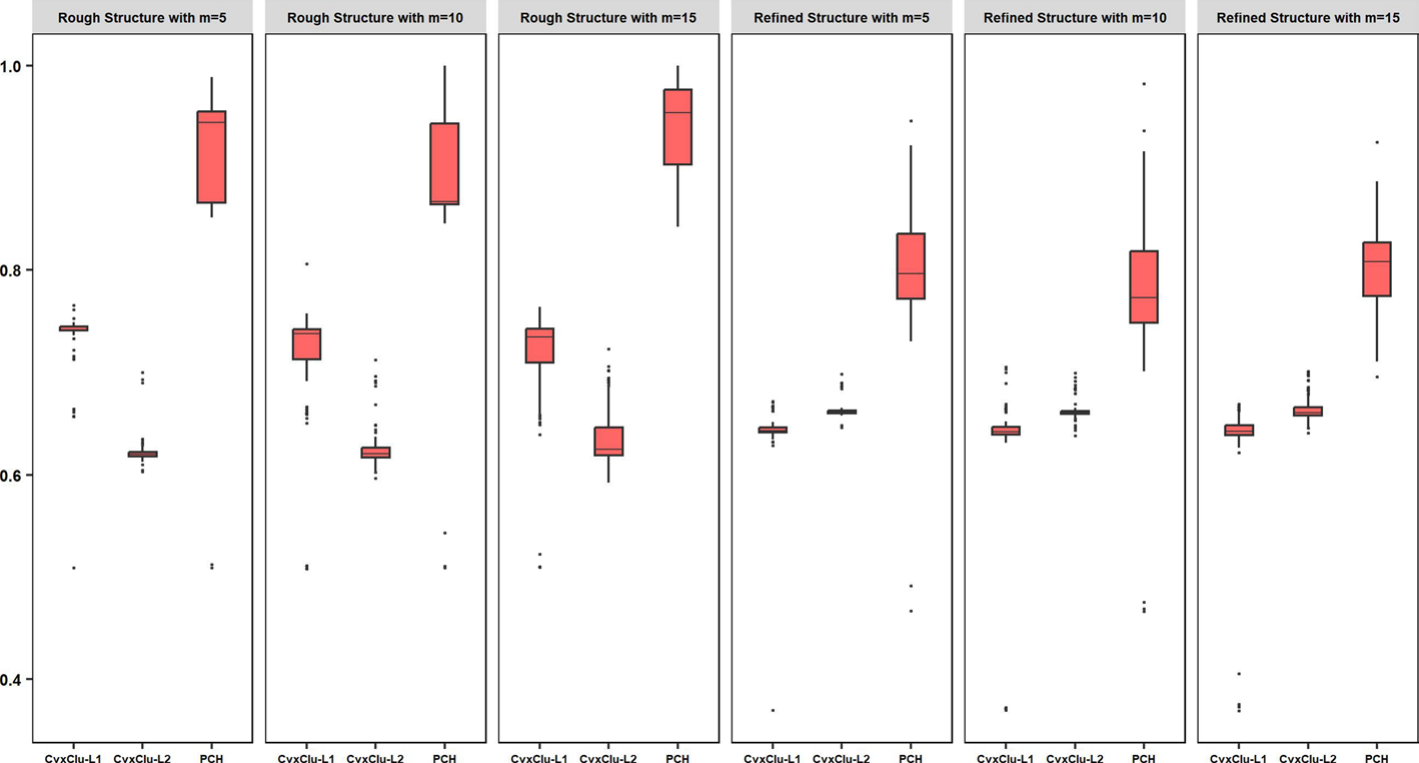
Analysis of LUAD data. In each subfigure, horizontal axis displays alternatives and our proposed method, and boxplot displays clustering similarity values of 100 replicates. From the left to the right, the case with $m=5,10,15$ for rough clustering structure, $m=5,10,15$ for refined clustering structure are orderly displayed

Since unsupervised learning lacks real response variables and sample labels, we cannot have a uniform criterion to measure the estimated clustering results against the real clustering structure. To further make comparison across different methods, we introduce some new definitions. For a clustering structure containing *n* subjects, we define a function $\Phi$ which maps each subject to its corresponding clustering label, and then define the similarity measure between two clustering mapping $\Phi _{1}$ and $\Phi _{2}$ as

$$\begin{aligned} {\textrm{SI}}\left( \Phi _{1},\Phi _{2}\right) =\frac{2}{n(n-1)}\sum _{1\leqslant j<m\leqslant n}\mathbb {I}\left\{ \mathbb {I}\left( \Phi _{1}\left( j\right) =\Phi _{1}\left( m\right) \right) =\mathbb {I}\left( \Phi _{2}\left( j\right) =\Phi _{2}\left( m\right) \right) \right\} . \end{aligned}$$

For an indirect evaluation, we examine the clustering stability, which shares the similar spirit of the stability selection in [44]. The concept underlying clustering stability is that an good clustering method should generate clustering structure that remains stable when subjected to slight perturbations across the whole samples. Specifically, we randomly remove *m* subjects of the whole 304 subjects with no respect to any prior information, and then analyzing the remaining $355-m$ subjects using our proposed method and alternatives. We set $m=5,10,15$, and repeat this procedure $R=100$ times. For the *r*-th replicate, denote the $\Phi _{1r}$ and $\Phi _{2r}$ as the new rough and refined clustering mappings, respectively, and denote $\Phi _{1r}^{*}$ and $\Phi _{2r}^{*}$ as the original analyzed rough and refined clustering mapping on remaining subjects, respectively. We calculate $\textrm{SI}\left( \Phi _{1r},\Phi _{1r}^{*}\right)$ and $\textrm{SI}\left( \Phi _{2r},\Phi _{2r}^{*}\right)$ for $r=1,\ldots ,100$, the means of which demonstrate clustering stability of each method. Table 4 and Fig. 3 show the comparison between our proposed method and alternatives. Our proposed method exhibits significantly higher stability compared to the alternatives regardless of the number of randomly removed subjects. This suggests that our method has a great advantage on resisting sampling perturbations in data distribution and yields a more stable clustering structure. Take the case with $m=5$ subjects removed as an example. As shown in Table 4, the reported mean similarity measure values for rough clustering are 0.7322 (CvxClu-$L_{1}$), 0.6223 (CvxClu-$L_{2}$), and 0.9159 (PCH), the reported mean similarity measure values for refined clustering are 0.6427 (CvxClu-$L_{1}$), 0.6631 (CvxClu-$L_{2}$), and 0.8005 (PCH). In addition, we also notice that the stability of the rough clustering surpasses that of the refined clustering, which is reasonable since the number of rough clusters is smaller. Nonetheless, the refined clustering structure maintains excellent stability with a mean similarity value exceeding 0.75 across all instances. Overall, these high stability values indirectly provide the validity of our proposed method.

## Discussion

Clustering is often the first step done for data analysis of cancer and other complex diseases. The identified subtypes can be used as an evidence for further therapies and other following analysis. Thus, it is important to develop efficient clustering method for complex data structure of cancer. In this paper, a prior-incorporated clustering framework with hierarchical penalties is proposed to integrate two types of features and produce biologically meaningful hierarchical structure. Theoretically, we establish statistical consistency properties on identification of clusters and estimation of center parameters, providing a solid ground for our method. Since we model hierarchical penalties, our theoretical contributions differ from the existing literature, and present significant complexity and challenge. A new efficient algorithm based on ADMM is developed for implementing our method. Simulation studies have shown highly competitive performance, exactly achieving progressive improvements with the assistance of prior information. Additionally, simulation results also indicate that our method is better suited for non-Gaussian clusters data, achieving higher clustering accuracy in various scenarios compared to alternatives. The analysis of LUAD data, combing with clinical imaging data and omics data, demonstrates the practical value in cancer biology. Specifically, we have indeed achieved hierarchical structure, and there are significant differences on clinical measurements among rough and refined clusters. Observed by *P*-values, the refined clustering structure provides a more significant difference among clusters, which implies that considering multi-level layers is necessary for a deeper exploration of the clustering nature behind biological data. Moreover, our method has maintained a remarkable stability compared to alternatives.

Despite the great achievements of our proposed method, there are still some potential problems. In our theoretical analysis, Theorem 1 is established under $n\gg q+p$, the ultra high-dimensional setting is intractable but worth studying. Moreover, due to dimensional limitation, we use PCA for pre-processing in our real data analysis, but confirmed with drawbacks in some studies [45, 46]. Hence, to handle high-dimensional problems in future research, we aim to extend our framework to feature selection task with additional sparse group penalty. In our computational implementation, we adopt BIC-type criterion, which is a common tuning technique in heterogeneity analysis [30, 31]. Some other tuning procedure is also suggested, such as cross validation and bootstrapping on clustering stability [44, 47], which may lead to different analysis results. Difference between clustering results leading by tuning also deserves further study. Note that the ADMM used in our computational algorithm can be also replaced by the alternating minimization algorithm (AMA; [48]). The efficiency and computational expenses of these algorithms deserve further investigation.

Furthermore, this work can serve as an inspiration for future research. The novel hierarchical penalties are not only applicable to imaging data and omics data, but also directly applied to any types of data with hierarchy, such as clinical and SNP data. The concept of clustering on hierarchical heterogeneous data can be also adopted in other clustering framework. Motivated by the innovative framework for fully utilizing prior pairwise relationships, one can extract and incorporate different kinds of prior information in multiple ways, such as integrating information between different datasets studying the same cancer, sharing the similar information patterns between different clustering methods. Additionally, the prior information can be extended to various types, such as certain samples not being expected to be assigned into the same cluster. Last but not the least, by minor modification on hierarchical penalties, our proposed method can be also extended to more intricate hierarchies with multiple layers.

## Supporting Information

The Supporting Information document contains the rigorous proof of theoretical results (referenced in section “Statistical properties”), the details of the computational algorithm (referenced in section “Computational algorithm”), and additional numerical results (referenced in sections “Simulation studies” and “Real data analysis”). R programs implementing the proposed method are available at GitHub (https://github.com/Hanw25).

### Supplementary information

**Additional file 1**. This file contains the proofs of theorems, details of the computational algorithm, and additional numerical results.

## Data Availability

The data that support the findings in this paper are openly available in TCGA (The Cancer Genome Atlas) at https://portal.gdc.cancer.gov/projects/TCGA-LUAD.
