# Supplementary material for: Clustering on hierarchical heterogeneous data with prior pairwise relationships
Source: BMC Bioinformatics. 2024 Jan 23;25:40. doi: 10.1186/s12859-024-05652-6 (PMC10807103; doi:10.1186/s12859-024-05652-6)
Supplement: Supplementary file 1 — Additional file 1. This file contains the proofs of theorems, details of the computational algorithm, and additional numerical results. [file 12859_2024_5652_MOESM1_ESM.pdf]

Supporting Information

of "Clustering on hierarchical heterogeneous data with prior pairwise relationships" by Wei Han, Sanguo Zhang, Hailong Gao, and Deliang Bu

A Proof of Theorem 1

A.1 Lemmas

Lemma 1. *If* $\boldsymbol{U} \in \mathbb{R}^d$ *follows sub-Gaussian distribution with variance proxy* σ_0^2 *and* $\mathrm{E}(\boldsymbol{U}) = \mathbf{0}$*, then with probability at least* $1-\delta$ *for* $\delta \in (0,1)$*,* $\|\boldsymbol{U}\|_2 \leqslant 4\sigma_0 d^{\frac{1}{2}} + 2\sigma_0 \left(2\log\left(\delta^{-1}\right)\right)^{\frac{1}{2}}$.

We define the oracle estimators as follows (that is, the minimizer under the true hierarchical clustering structure):

$$\left(\widehat{\boldsymbol{\xi}}^0, \widehat{\boldsymbol{\alpha}}^0\right) = \arg\min_{\boldsymbol{\xi},\boldsymbol{\alpha}} \left\{ \frac{1}{2}\sum_{k_1=1}^{K_1}\sum_{i\in\mathcal{G}_{k_1}^*} \left\|\boldsymbol{\xi}_{k_1} - \boldsymbol{X}_i\right\|_2^2 + \frac{1}{2}\sum_{k_2=1}^{K_2}\sum_{i\in\mathcal{T}_{k_2}^*} \left\|\boldsymbol{\alpha}_{k_2} - \boldsymbol{Z}_i\right\|_2^2 \right\},$$

where $\widehat{\boldsymbol{\xi}}^0 = \left(\widehat{\boldsymbol{\xi}}_1^0, \cdots, \widehat{\boldsymbol{\xi}}_{K_1}^0\right) \in \mathbb{R}^q \times \mathbb{R}^{K_1}$ and $\widehat{\boldsymbol{\alpha}}^0 = \left(\widehat{\boldsymbol{\alpha}}_1^0, \cdots, \widehat{\boldsymbol{\alpha}}_{K_2}^0\right) \in \mathbb{R}^p \times \mathbb{R}^{K_2}$. Let $\widehat{\boldsymbol{\beta}}_i^0 = \widehat{\boldsymbol{\xi}}_{k_1}^0$ and $\widehat{\boldsymbol{\gamma}}_i^0 = \widehat{\boldsymbol{\alpha}}_{k_2}^0$ if i belongs to $\mathcal{G}_{k_1}^*$ and $\mathcal{T}_{k_2}^*$. Here, we introduce the following Lemmas.

Lemma 2. *Define* $\phi_n = (q+p)^{\frac{1}{2}} T_{\min}^{-\frac{1}{2}} (\log n)^{\frac{1}{2}}$. *Under Condition 1, there exists a constant* C*, such that with probability at least* $1 - 2n^{-1}$*, the oracle estimators* $\left(\widehat{\boldsymbol{\xi}}^0, \widehat{\boldsymbol{\alpha}}^0\right)$ *satisfy that*

$$\sup_{1\leqslant k_1\leqslant K_1} \left\|\widehat{\boldsymbol{\xi}}_{k_1}^0 - \boldsymbol{\xi}_{k_1}^*\right\|_2 \leqslant C\phi_n, \quad \sup_{1\leqslant k_2\leqslant K_2} \left\|\widehat{\boldsymbol{\alpha}}_{k_2}^0 - \boldsymbol{\alpha}_{k_2}^*\right\|_2 \leqslant C\phi_n,$$

Lemma 3. *Suppose that the conditions in Theorem 1 hold. There exists a local minimizer* $\left(\widehat{\boldsymbol{\beta}}, \widehat{\boldsymbol{\gamma}}\right)$ *of* $Q(\boldsymbol{\beta}, \boldsymbol{\gamma})$ *subject to* $\boldsymbol{\beta} \in \mathcal{M}_1$ *and* $\boldsymbol{\gamma} \in \mathcal{M}_2$ *satisfying that as* $n \to \infty$,

$$\Pr\left(\left(\widehat{\boldsymbol{\beta}}, \widehat{\boldsymbol{\gamma}}\right) = \left(\widehat{\boldsymbol{\beta}}^0, \widehat{\boldsymbol{\gamma}}^0\right)\right) \to 1.$$

Here, we proof Theorem 1 by Lemmas 2 and 3. For any $\delta \in (0,1)$, there exists sufficiently large n, such that there exists a local minimizer $\left(\widehat{\boldsymbol{\beta}}, \widehat{\boldsymbol{\gamma}}\right)$ of $Q(\boldsymbol{\beta}, \boldsymbol{\gamma})$, with probability at least $1-\frac{\delta}{2}$, $\left(\widehat{\boldsymbol{\beta}}, \widehat{\boldsymbol{\gamma}}\right) = \left(\widehat{\boldsymbol{\beta}}^0, \widehat{\boldsymbol{\gamma}}^0\right)$ holds, and with probability at least $1-2n^{-1} > 1-\frac{\delta}{2}$,

$$\sup_{1 \leqslant k_1 \leqslant K_1} \left\| \widehat{\boldsymbol{\xi}}_{k_1}^0 - \boldsymbol{\xi}_{k_1}^* \right\|_2 \leqslant C\phi_n, \quad \sup_{1 \leqslant k_2 \leqslant K_2} \left\| \widehat{\boldsymbol{\alpha}}_{k_2}^0 - \boldsymbol{\alpha}_{k_2}^* \right\|_2 \leqslant C\phi_n.$$

Hence, with probability at least $1-\delta$, the above hold simultaneously. Observe that oracle estimators can recover true clustering structure, hence $\left(\widehat{\boldsymbol{\beta}}, \widehat{\boldsymbol{\gamma}}\right) = \left(\widehat{\boldsymbol{\beta}}^0, \widehat{\boldsymbol{\gamma}}^0\right)$ implies that $\widehat{K}_1 = K_1$, $\widehat{K}_2 = K_2$, $\widehat{\mathcal{G}}_{k_1} = \mathcal{G}_{k_1}^*$, and $\widehat{\mathcal{T}}_{k_2} = \mathcal{T}_{k_2}^*$. Therefore, Theorem 1 is proved.

A.2 Proofs of Lemmas

Lemma 1 can be referred to Theorem 1.19 in [1] (https://math.mit.edu/~rigollet/PDFs/RigNotes17.pdf), which is a classical textbook in high dimensional statistics. We firstly give a rigorous proof of Lemma 2. For $k_1 = 1, \cdots, K_1$ and $k_2 = 1, \cdots, K_2$, the oracle estimators have analytic solutions,

$$\widehat{\boldsymbol{\xi}}_{k_1}^0 = \left|\mathcal{G}_{k_1}^*\right|^{-1} \sum_{i \in \mathcal{G}_{k_1}^*} \boldsymbol{X}_i, \quad \widehat{\boldsymbol{\alpha}}_{k_2}^0 = \left|\mathcal{T}_{k_2}^*\right|^{-1} \sum_{i \in \mathcal{T}_{k_2}^*} \boldsymbol{Z}_i.$$

Directly, for $k_1 = 1, \cdots, K_1$, we have that

$$\widehat{\boldsymbol{\xi}}_{k_1}^0 - \boldsymbol{\xi}_{k_1}^* = \left|\mathcal{G}_{k_1}^*\right|^{-1} \sum_{i \in \mathcal{G}_{k_1}^*} \boldsymbol{X}_i - \boldsymbol{\xi}_{k_1}^* = \left|\mathcal{G}_{k_1}^*\right|^{-1} \sum_{i \in \mathcal{G}_{k_1}^*} \left(\boldsymbol{\xi}_{k_1}^* + \boldsymbol{\epsilon}_{1i}\right) - \boldsymbol{\xi}_{k_1}^* = \left|\mathcal{G}_{k_1}^*\right|^{-1} \sum_{i \in \mathcal{G}_{k_1}^*} \boldsymbol{\epsilon}_{1i}.$$

Note that $\left|\mathcal{G}_{k_1}^*\right|^{-1} \sum_{i \in \mathcal{G}_{k_1}^*} \boldsymbol{\epsilon}_{1i}$ has mean $\mathbf{0}$ and follows sub-Gaussian distribution with variance proxy $\left|\mathcal{G}_{k_1}^*\right|^{-1} \sigma_0^2$. By Lemma 1, for fixed k_1, with probability at least $1 - n^{-2}$,

$$\left\| \widehat{\boldsymbol{\xi}}_{k_1}^0 - \boldsymbol{\xi}_{k_1}^* \right\|_2 \leqslant 4 \left|\mathcal{G}_{k_1}^*\right|^{-\frac{1}{2}} \sigma_0 q^{\frac{1}{2}} + 2 \left|\mathcal{G}_{k_1}^*\right|^{-\frac{1}{2}} \sigma_0 (4 \log n)^{\frac{1}{2}} \leqslant 8 \sigma_0 q^{\frac{1}{2}} T_{\min}^{-\frac{1}{2}} (\log n)^{\frac{1}{2}}.$$

Similarly, for fixed k_2, with probability at least $1-n^{-2}$,

$$\left\|\widehat{\boldsymbol{\alpha}}_{k_2}^0-\boldsymbol{\alpha}_{k_2}^*\right\|_2 \leqslant 4\left|\mathcal{T}_{k_2}^*\right|^{-\frac{1}{2}} \sigma_0 p^{\frac{1}{2}}+2\left|\mathcal{T}_{k_2}^*\right|^{-\frac{1}{2}} \sigma_0(4 \log n)^{\frac{1}{2}} \leqslant 8 \sigma_0 p^{\frac{1}{2}} T_{\min }^{-\frac{1}{2}}(\log n)^{\frac{1}{2}} .$$

Note that $\phi_n=(q+p)^{\frac{1}{2}} T_{\min }^{-\frac{1}{2}}(\log n)^{\frac{1}{2}}$. There exists a constant C, such that with probability at least $1-\left(K_1+K_2\right) n^{-2}>1-2 n^{-1}$, it holds that

$$\sup _{1 \leqslant k_1 \leqslant K_1}\left\|\widehat{\boldsymbol{\xi}}_{k_1}^0-\boldsymbol{\xi}_{k_1}^*\right\|_2 \leqslant C \phi_n, \quad \sup _{1 \leqslant k_2 \leqslant K_2}\left\|\widehat{\boldsymbol{\alpha}}_{k_2}^0-\boldsymbol{\alpha}_{k_2}^*\right\|_2 \leqslant C \phi_n . \tag{*}$$

Hence, Lemma 2 is proved. Then we give a rigorous proof of Lemma 3. Define the following two sets,

$$\begin{aligned}
& \mathcal{M}_{\mathcal{G}}=\left\{\boldsymbol{\beta} \in \mathbb{R}^q \times \mathbb{R}^n: \boldsymbol{\beta}_j=\boldsymbol{\beta}_m, j, m \in \mathcal{G}_{k_1}^*, k_1=1, \cdots, K_1\right\}, \\
& \mathcal{M}_{\mathcal{T}}=\left\{\boldsymbol{\gamma} \in \mathbb{R}^p \times \mathbb{R}^n: \boldsymbol{\gamma}_j=\boldsymbol{\gamma}_m, j, m \in \mathcal{T}_{k_2}^*, k_2=1, \cdots, K_2\right\} .
\end{aligned}$$

Recall that

$$\begin{aligned}
& \mathcal{M}_1=\left\{\boldsymbol{\beta} \in \mathbb{R}^q \times \mathbb{R}^n: \boldsymbol{\beta}_j=\boldsymbol{\beta}_m,(j, m) \in \mathcal{A}^{\mathrm{p}}\right\}, \\
& \mathcal{M}_2=\left\{\boldsymbol{\gamma} \in \mathbb{R}^p \times \mathbb{R}^n: \boldsymbol{\gamma}_j=\boldsymbol{\gamma}_m,(j, m) \in \mathcal{A}^{\mathrm{p}}\right\} .
\end{aligned}$$

Since the prior information does not violate the true heterogeneous structure, it is obvious that $\mathcal{M}_{\mathcal{G}} \subset \mathcal{M}_1$ and $\mathcal{M}_{\mathcal{T}} \subset \mathcal{M}_2$.

Let $\Omega_1: \mathcal{M}_{\mathcal{G}} \rightarrow \mathbb{R}^q \times \mathbb{R}^{K_1}$ be the one-to-one mapping that $\Omega_1(\boldsymbol{\beta})$ is the $q \times K_1$ matrix consisting of K_1 column vectors with dimension q and its k_1-th column equals to the common value of $\boldsymbol{\beta}_i$ for $i \in \mathcal{G}_{k_1}^*$. Let $\breve{\Omega}_1: \mathcal{M}_1 \rightarrow \mathbb{R}^q \times \mathbb{R}^{K_1}$ be the mapping that

$$\breve{\Omega}_1(\boldsymbol{\beta})=\left\{\left|\mathcal{G}_{k_1}^*\right|^{-1} \sum_{i \in \mathcal{G}_{k_1}^*} \boldsymbol{\beta}_i, k_1=1, \cdots, K_1\right\} .$$

Let $\Omega_2: \mathcal{M}_{\mathcal{T}} \rightarrow \mathbb{R}^p \times \mathbb{R}^{K_2}$ be the one-to-one mapping that $\Omega_2(\boldsymbol{\gamma})$ is the $p \times K_2$ matrix consisting of K_2 column vectors with dimension p and its k_2-th column equals to the common

value of $\boldsymbol{\gamma}_i$ for $i \in \mathcal{T}_{k_2}^*$. Let $\breve{\Omega}_2 : \mathcal{M}_2 \to \mathbb{R}^p \times \mathbb{R}^{K_2}$ be the mapping that

$$\breve{\Omega}_2(\boldsymbol{\gamma}) = \left\{ \left|\mathcal{T}_{k_2}^*\right|^{-1} \sum_{i \in \mathcal{T}_{k_2}^*} \boldsymbol{\gamma}_i, k_2 = 1, \cdots, K_2 \right\}.$$

Clearly, when $\boldsymbol{\beta} \in \mathcal{M}_{\mathcal{G}}$ and $\boldsymbol{\gamma} \in \mathcal{M}_{\mathcal{T}}$, $\Omega_1(\boldsymbol{\beta}) = \breve{\Omega}_1(\boldsymbol{\beta})$ and $\Omega_2(\boldsymbol{\gamma}) = \breve{\Omega}_2(\boldsymbol{\gamma})$. For any $\boldsymbol{\beta} \in \mathcal{M}_1$ and $\boldsymbol{\gamma} \in \mathcal{M}_2$, define $\breve{\boldsymbol{\beta}} = \Omega_1^{-1}\left(\breve{\Omega}_1(\boldsymbol{\beta})\right)$ and $\breve{\boldsymbol{\gamma}} = \Omega_2^{-1}\left(\breve{\Omega}_2(\boldsymbol{\gamma})\right)$. Then, we consider the neighborhoods of $(\boldsymbol{\beta}^*, \boldsymbol{\gamma}^*)$ and $\left(\widehat{\boldsymbol{\beta}}^0, \widehat{\boldsymbol{\gamma}}^0\right)$,

$$\Theta = \left\{(\boldsymbol{\beta}, \boldsymbol{\gamma}) : \boldsymbol{\beta} \in \mathcal{M}_1, \boldsymbol{\gamma} \in \mathcal{M}_2, \|\boldsymbol{\beta}_i - \boldsymbol{\beta}_i^*\|_2 \leqslant C\phi_n, \|\boldsymbol{\gamma}_i - \boldsymbol{\gamma}_i^*\|_2 \leqslant C\phi_n\right\},$$

$$\Theta_n = \left\{(\boldsymbol{\beta}, \boldsymbol{\gamma}) : \boldsymbol{\beta} \in \mathcal{M}_1, \boldsymbol{\gamma} \in \mathcal{M}_2, \left\|\boldsymbol{\beta}_i - \widehat{\boldsymbol{\beta}}_i^0\right\|_2 \leqslant \psi_n, \|\boldsymbol{\gamma}_i - \widehat{\boldsymbol{\gamma}}_i^0\|_2 \leqslant \psi_n\right\},$$

where sequence $\{\psi_n\}$ satisfies $\psi_n \to 0$. By Lemma 2, with probability at least $1 - 2n^{-1}$, (*) holds. The proof of Lemma 3 contains two steps, (1) When (*) holds, for any $(\boldsymbol{\beta}, \boldsymbol{\gamma}) \in \Theta$, we have $\left(\breve{\boldsymbol{\beta}}, \breve{\boldsymbol{\gamma}}\right) \in \Theta$ and $Q\left(\breve{\boldsymbol{\beta}}, \breve{\boldsymbol{\gamma}}\right) \geqslant Q\left(\widehat{\boldsymbol{\beta}}^0, \widehat{\boldsymbol{\gamma}}^0\right)$; (2) With probability at least $1-2n^{-1}$, for any $(\boldsymbol{\beta}, \boldsymbol{\gamma}) \in \Theta \cap \Theta_n$, we have that $Q(\boldsymbol{\beta}, \boldsymbol{\gamma}) \geqslant Q\left(\breve{\boldsymbol{\beta}}, \breve{\boldsymbol{\gamma}}\right)$.

Now we give the proofs of (1) and (2). With respect to $\boldsymbol{\beta} \in \mathcal{M}_1$ and $\boldsymbol{\gamma} \in \mathcal{M}_2$, we decompose $Q(\boldsymbol{\beta}, \boldsymbol{\gamma})$ into two parts $L(\boldsymbol{\beta}, \boldsymbol{\gamma})$ and $P(\boldsymbol{\beta}, \boldsymbol{\gamma})$, where the loss is

$$L(\boldsymbol{\beta}, \boldsymbol{\gamma}) = \frac{1}{2} \sum_{i=1}^n \left(\|\boldsymbol{X}_i - \boldsymbol{\beta}_i\|_2^2 + \|\boldsymbol{Z}_i - \boldsymbol{\gamma}_i\|_2^2\right),$$

and the penalties are

$$\begin{aligned} P(\boldsymbol{\beta}, \boldsymbol{\gamma}) &= \sum_{(j,m)\in\mathcal{A}} p\left(\left(\left\|\boldsymbol{\beta}_j - \boldsymbol{\beta}_m\right\|_2^2 + \left\|\boldsymbol{\gamma}_j - \boldsymbol{\gamma}_m\right\|_2^2\right)^{\frac{1}{2}}; \lambda_1\right) + \sum_{(j,m)\in\mathcal{A}} p\left(\left\|\boldsymbol{\beta}_j - \boldsymbol{\beta}_m\right\|_2; \lambda_2\right) \\ &= \sum_{(j,m)\in\mathcal{A}\backslash\mathcal{A}^{\mathrm{p}}} p\left(\left(\left\|\boldsymbol{\beta}_j - \boldsymbol{\beta}_m\right\|_2^2 + \left\|\boldsymbol{\gamma}_j - \boldsymbol{\gamma}_m\right\|_2^2\right)^{\frac{1}{2}}; \lambda_1\right) + \sum_{(j,m)\in\mathcal{A}\backslash\mathcal{A}^{\mathrm{p}}} p\left(\left\|\boldsymbol{\beta}_j - \boldsymbol{\beta}_m\right\|_2; \lambda_2\right). \end{aligned}$$

For any $(\boldsymbol{\beta}, \boldsymbol{\gamma}) \in \Theta$, we have $\|\boldsymbol{\beta}_i - \boldsymbol{\beta}_i^*\|_2 \leqslant C\phi_n$ and $\|\boldsymbol{\gamma}_i - \boldsymbol{\gamma}_i^*\|_2 \leqslant C\phi_n$, then

$$\|\breve{\boldsymbol{\gamma}}_i - \boldsymbol{\gamma}_i^*\|_2 = \left\| \left|\mathcal{T}_{k_2}^*\right|^{-1} \sum_{i \in \mathcal{T}_{k_2}^*} (\boldsymbol{\gamma}_i - \boldsymbol{\gamma}_i^*) \right\|_2 \leqslant \left|\mathcal{T}_{k_2}^*\right|^{-1} \sum_{i \in \mathcal{T}_{k_2}^*} \|\boldsymbol{\gamma}_i - \boldsymbol{\gamma}_i^*\|_2 \leqslant C\phi_n.$$

Similarly, $\left\|\breve{\boldsymbol{\beta}}_i - \boldsymbol{\beta}_i^*\right\|_2 \leqslant C\phi_n$. Hence, $\left(\breve{\boldsymbol{\beta}}, \breve{\boldsymbol{\gamma}}\right) \in \Theta$. Let $\Omega_1(\boldsymbol{\beta}) = \boldsymbol{\xi} = \left(\boldsymbol{\xi}_1, \cdots, \boldsymbol{\xi}_{K_1}\right)$, by definition, $\boldsymbol{\xi}_{k_1} = \left|\mathcal{G}_{k_1}^*\right|^{-1} \sum_{i \in \mathcal{G}_{k_1}^*} \boldsymbol{\beta}_i$ for $k_1 = 1, \cdots, K_1$ and $\breve{\boldsymbol{\beta}} = \Omega_1^{-1}\left(\breve{\Omega}_1(\boldsymbol{\beta})\right)$ whose i-th column vector is $\boldsymbol{\xi}_{k_1}$ for $i \in \mathcal{G}_{k_1}^*$. Since $\left(\breve{\boldsymbol{\beta}}, \breve{\boldsymbol{\gamma}}\right) \in \Theta$, we have that $\left\|\boldsymbol{\xi}_{k_1} - \boldsymbol{\xi}_{k_1}^*\right\|_2 \leqslant C\phi_n$ for $k_1 = 1, \cdots, K_1$. Hence, for any $1 \leqslant k_1 \neq k_1' \leqslant K_1$,

$$\begin{aligned}
\left\|\boldsymbol{\xi}_{k_1} - \boldsymbol{\xi}_{k_1'}\right\|_2 &\geqslant \left\|\boldsymbol{\xi}_{k_1}^* - \boldsymbol{\xi}_{k_1'}^*\right\|_2 - \left\|\boldsymbol{\xi}_{k_1}^* - \boldsymbol{\xi}_{k_1}\right\|_2 - \left\|\boldsymbol{\xi}_{k_1'} - \boldsymbol{\xi}_{k_1'}^*\right\|_2 \\
&\geqslant b_n - 2C\phi_n > (a + \kappa)\lambda_2 - 2C\phi_n > a\lambda_2.
\end{aligned}$$

Therefore, $p\left(\left\|\boldsymbol{\xi}_{k_1} - \boldsymbol{\xi}_{k_1'}\right\|_2; \lambda_2\right)$ is a constant. When (*) holds, in the same discussion, $p\left(\left\|\widehat{\boldsymbol{\xi}}_{k_1}^0 - \widehat{\boldsymbol{\xi}}_{k_1'}^0\right\|_2; \lambda_2\right)$ is the same constant. Hence,

$$\sum_{(j,m) \in \mathcal{A}} p\left(\left\|\breve{\boldsymbol{\beta}}_j - \breve{\boldsymbol{\beta}}_m\right\|_2; \lambda_2\right) = \sum_{(j,m) \in \mathcal{A}} p\left(\left\|\widehat{\boldsymbol{\beta}}_j^0 - \widehat{\boldsymbol{\beta}}_m^0\right\|_2; \lambda_2\right).$$

In a similar way, $\left\|\boldsymbol{\alpha}_{k_2} - \boldsymbol{\alpha}_{k_2'}\right\|_2 \geqslant d_n - 2C\phi_n > a\lambda_1$. Therefore, for any $1 \leqslant k_2 \neq k_2' \leqslant K_2$,

$$p\left(\left(\left\|\boldsymbol{\xi}_{h(k_2)} - \boldsymbol{\xi}_{h(k_2\prime)}\right\|_2^2 + \left\|\boldsymbol{\alpha}_{k_2} - \boldsymbol{\alpha}_{k_2'}\right\|_2^2\right)^{\frac{1}{2}}; \lambda_1\right)$$

is a constant, where $h(k_2) = \sum_{k_1=1}^{K_1} k_1 \mathbb{I}\left(k_2 \in \mathcal{H}_{k_1}^*\right)$. Hence,

$$\begin{aligned}
&\sum_{(j,m) \in \mathcal{A}} p\left(\left(\left\|\breve{\boldsymbol{\beta}}_j - \breve{\boldsymbol{\beta}}_m\right\|_2^2 + \left\|\breve{\boldsymbol{\gamma}}_j - \breve{\boldsymbol{\gamma}}_m\right\|_2^2\right)^{\frac{1}{2}}; \lambda_1\right) \\
&= \sum_{(j,m) \in \mathcal{A}} p\left(\left(\left\|\widehat{\boldsymbol{\beta}}_j^0 - \widehat{\boldsymbol{\beta}}_m^0\right\|_2^2 + \left\|\widehat{\boldsymbol{\gamma}}_j^0 - \widehat{\boldsymbol{\gamma}}_m^0\right\|_2^2\right)^{\frac{1}{2}}; \lambda_1\right).
\end{aligned}$$

Hence, $P\left(\breve{\boldsymbol{\beta}},\breve{\boldsymbol{\gamma}}\right)=P\left(\widehat{\boldsymbol{\beta}}^{0},\widehat{\boldsymbol{\gamma}}^{0}\right)$. Since $\left(\widehat{\boldsymbol{\beta}}^{0},\widehat{\boldsymbol{\gamma}}^{0}\right)$ is the global minimizer of $L\left(\boldsymbol{\beta},\boldsymbol{\gamma}\right)$ subject to $\boldsymbol{\beta}\in\mathcal{M}_{\mathcal{G}}$ and $\boldsymbol{\gamma}\in\mathcal{M}_{\mathcal{T}}$, we have that $Q\left(\breve{\boldsymbol{\beta}},\breve{\boldsymbol{\gamma}}\right)\geqslant Q\left(\widehat{\boldsymbol{\beta}}^{0},\widehat{\boldsymbol{\gamma}}^{0}\right)$. Hence, (1) is proved.

For any $(\boldsymbol{\beta},\boldsymbol{\gamma})\in\Theta\cap\Theta_n$, we have that $\left(\breve{\boldsymbol{\beta}},\breve{\boldsymbol{\gamma}}\right)\in\Theta\cap\Theta_n$. By Taylor's expansion, let $\bar{\boldsymbol{\beta}}=\chi\boldsymbol{\beta}+(1-\chi)\breve{\boldsymbol{\beta}}$ and $\bar{\boldsymbol{\gamma}}=\chi\boldsymbol{\gamma}+(1-\chi)\breve{\boldsymbol{\gamma}}$ where $\chi\in[0,1]$, we have that $Q\left(\boldsymbol{\beta},\boldsymbol{\gamma}\right)-Q\left(\breve{\boldsymbol{\beta}},\breve{\boldsymbol{\gamma}}\right)=\Gamma_1+\Gamma_2+\Gamma_3$, where

$$\Gamma_1=-\sum_{i=1}^{n}\left(\boldsymbol{X}_i-\bar{\boldsymbol{\beta}}_i\right)^{\mathrm{T}}\left(\boldsymbol{\beta}_i-\breve{\boldsymbol{\beta}}_i\right)-\sum_{i=1}^{n}\left(\boldsymbol{Z}_i-\bar{\boldsymbol{\gamma}}_i\right)^{\mathrm{T}}\left(\boldsymbol{\gamma}_i-\breve{\boldsymbol{\gamma}}_i\right)\triangleq\Gamma_{11}+\Gamma_{12},$$

$$\Gamma_2=\sum_{(j,m)\in\mathcal{A}\backslash\mathcal{A}^{\mathrm{p}},(m,j)\in\mathcal{A}\backslash\mathcal{A}^{\mathrm{p}}}p'\left(\left\|\bar{\boldsymbol{\theta}}_j-\bar{\boldsymbol{\theta}}_m\right\|_2;\lambda_1\right)\left\|\bar{\boldsymbol{\theta}}_j-\bar{\boldsymbol{\theta}}_m\right\|_2^{-1}\left(\bar{\boldsymbol{\theta}}_j-\bar{\boldsymbol{\theta}}_m\right)^{\mathrm{T}}\left(\boldsymbol{\theta}_j-\breve{\boldsymbol{\theta}}_j\right),$$

$$\Gamma_3=\sum_{(j,m)\in\mathcal{A}\backslash\mathcal{A}^{\mathrm{p}},(m,j)\in\mathcal{A}\backslash\mathcal{A}^{\mathrm{p}}}p'\left(\left\|\bar{\boldsymbol{\beta}}_j-\bar{\boldsymbol{\beta}}_m\right\|_2;\lambda_2\right)\left\|\bar{\boldsymbol{\beta}}_j-\bar{\boldsymbol{\beta}}_m\right\|_2^{-1}\left(\bar{\boldsymbol{\beta}}_j-\bar{\boldsymbol{\beta}}_m\right)^{\mathrm{T}}\left(\boldsymbol{\beta}_j-\breve{\boldsymbol{\beta}}_j\right),$$

where $\boldsymbol{\theta}_i=\left(\boldsymbol{\beta}_i^{\mathrm{T}},\boldsymbol{\gamma}_i^{\mathrm{T}}\right)^{\mathrm{T}}$. For Γ_{11}, let $\boldsymbol{B}_{1j}=\boldsymbol{X}_j-\bar{\boldsymbol{\beta}}_j$, we have that

$$\begin{aligned}\Gamma_{11}&=-\sum_{j=1}^{n}\boldsymbol{B}_{1j}^{\mathrm{T}}\left(\boldsymbol{\beta}_j-\breve{\boldsymbol{\beta}}_j\right)=-\sum_{k_1=1}^{K_1}\sum_{j\in\mathcal{G}_{k_1}^*}\sum_{m\in\mathcal{G}_{k_1}^*}\left|\mathcal{G}_{k_1}^*\right|^{-1}\boldsymbol{B}_{1j}^{\mathrm{T}}\left(\boldsymbol{\beta}_j-\boldsymbol{\beta}_m+\boldsymbol{\beta}_m-\breve{\boldsymbol{\beta}}_j\right)\\&=-\sum_{k_1=1}^{K_1}\sum_{j,m\in\mathcal{G}_{k_1}^*}\left|\mathcal{G}_{k_1}^*\right|^{-1}\boldsymbol{B}_{1j}^{\mathrm{T}}\left(\boldsymbol{\beta}_j-\boldsymbol{\beta}_m\right)-\sum_{k_1=1}^{K_1}\sum_{j\in\mathcal{G}_{k_1}^*}\boldsymbol{B}_{1j}^{\mathrm{T}}\left(\sum_{m\in\mathcal{G}_{k_1}^*}\left|\mathcal{G}_{k_1}^*\right|^{-1}\boldsymbol{\beta}_m-\breve{\boldsymbol{\beta}}_j\right).\end{aligned}$$

Note that for $j,m\in\mathcal{G}_{k_1}^*$, $\breve{\boldsymbol{\beta}}_j=\breve{\boldsymbol{\beta}}_m=\sum_{m\in\mathcal{G}_{k_1}^*}\left|\mathcal{G}_{k_1}^*\right|^{-1}\boldsymbol{\beta}_m$, then

$$\begin{aligned}\Gamma_{11}&=-\sum_{k_1=1}^{K_1}\sum_{j,m\in\mathcal{G}_{k_1}^*}\frac{1}{2}\left|\mathcal{G}_{k_1}^*\right|^{-1}\left[\boldsymbol{B}_{1j}^{\mathrm{T}}\left(\boldsymbol{\beta}_j-\boldsymbol{\beta}_m\right)+\boldsymbol{B}_{1m}^{\mathrm{T}}\left(\boldsymbol{\beta}_m-\boldsymbol{\beta}_j\right)\right]\\&=-\sum_{k_1=1}^{K_1}\sum_{j,m\in\mathcal{G}_{k_1}^*,(j,m)\in\mathcal{A}\backslash\mathcal{A}^{\mathrm{p}}}\left|\mathcal{G}_{k_1}^*\right|^{-1}\left(\boldsymbol{B}_{1j}-\boldsymbol{B}_{1m}\right)^{\mathrm{T}}\left(\boldsymbol{\beta}_j-\boldsymbol{\beta}_m\right)\\&\geqslant-2\sup_{1\leqslant i\leqslant n}\left\|\boldsymbol{B}_{1i}\right\|_2\sum_{k_1=1}^{K_1}\sum_{j,m\in\mathcal{G}_{k_1}^*,(j,m)\in\mathcal{A}\backslash\mathcal{A}^{\mathrm{p}}}G_{\min}^{-1}\left\|\boldsymbol{\beta}_j-\boldsymbol{\beta}_m\right\|_2.\end{aligned}$$

Note that $\boldsymbol{X}_i = \boldsymbol{\beta}_i^* + \boldsymbol{\epsilon}_{1i}$, then we have $\boldsymbol{B}_{1i} = \boldsymbol{\epsilon}_{1i} + \chi\left(\boldsymbol{\beta}_i^* - \boldsymbol{\beta}_i\right) + (1-\chi)\left(\boldsymbol{\beta}_i^* - \breve{\boldsymbol{\beta}}_i\right)$. Hence,

$$\left\|\boldsymbol{B}_{1i}\right\|_2 \leqslant \left\|\boldsymbol{\epsilon}_{1i}\right\|_2 + \chi\left\|\boldsymbol{\beta}_i^* - \boldsymbol{\beta}_i\right\|_2 + (1-\chi)\left\|\boldsymbol{\beta}_i^* - \breve{\boldsymbol{\beta}}_i\right\|_2 \leqslant \left\|\boldsymbol{\epsilon}_{1i}\right\|_2 + C\phi_n.$$

Note that $\boldsymbol{\epsilon}_{1i}$ follows sub-Gaussian distribution with variance proxy σ_0^2 and $\mathrm{E}\left(\boldsymbol{\epsilon}_{1i}\right) = \mathbf{0}$. By Lemma 1, for fixed i, with probability at least $1 - n^{-2}$, $\left\|\boldsymbol{\epsilon}_{1i}\right\|_2 \leqslant 4\sigma_0 q^{\frac{1}{2}} + 2\sigma_0\left(4\log n\right)^{\frac{1}{2}}$. Hence, with probability at least $1 - n^{-1}$,

$$\sup_{1\leqslant i\leqslant n}\left\|\boldsymbol{B}_{1i}\right\|_2 \leqslant C_1\left(q^{\frac{1}{2}} + (\log n)^{\frac{1}{2}}\right),$$

where C_1 is a positive constant. Then,

$$\Gamma_{11} \geqslant -\sum_{k_1=1}^{K_1}\sum_{j,m\in\mathcal{G}_{k_1}^*,(j,m)\in\mathcal{A}\setminus\mathcal{A}^{\mathrm{p}}} 2C_1 G_{\min}^{-1}\left(q^{\frac{1}{2}} + (\log n)^{\frac{1}{2}}\right)\left\|\boldsymbol{\beta}_j - \boldsymbol{\beta}_m\right\|_2.$$

Similarly, there exists a positive constant C_2, with probability at least $1 - n^{-1}$,

$$\begin{aligned}\Gamma_{12} &\geqslant -\sum_{k_2=1}^{K_2}\sum_{j,m\in\mathcal{T}_{k_2}^*,(j,m)\in\mathcal{A}\setminus\mathcal{A}^{\mathrm{p}}} 2C_2 T_{\min}^{-1}\left(p^{\frac{1}{2}} + (\log n)^{\frac{1}{2}}\right)\left\|\boldsymbol{\gamma}_j - \boldsymbol{\gamma}_m\right\|_2\\ &\geqslant -\sum_{k_2=1}^{K_2}\sum_{j,m\in\mathcal{T}_{k_2}^*,(j,m)\in\mathcal{A}\setminus\mathcal{A}^{\mathrm{p}}} 2C_2 T_{\min}^{-1}\left(p^{\frac{1}{2}} + (\log n)^{\frac{1}{2}}\right)\left\|\boldsymbol{\theta}_j - \boldsymbol{\theta}_m\right\|_2.\end{aligned}$$

Hence, with probability at least $1 - 2n^{-1}$, the following holds,

$$\begin{aligned}\Gamma_1 \geqslant &-\sum_{k_1=1}^{K_1}\sum_{j,m\in\mathcal{G}_{k_1}^*,(j,m)\in\mathcal{A}\setminus\mathcal{A}^{\mathrm{p}}} 2C_1 G_{\min}^{-1}\left(q^{\frac{1}{2}} + (\log n)^{\frac{1}{2}}\right)\left\|\boldsymbol{\beta}_j - \boldsymbol{\beta}_m\right\|_2\\ &-\sum_{k_2=1}^{K_2}\sum_{j,m\in\mathcal{T}_{k_2}^*,(j,m)\in\mathcal{A}\setminus\mathcal{A}^{\mathrm{p}}} 2C_2 T_{\min}^{-1}\left(p^{\frac{1}{2}} + (\log n)^{\frac{1}{2}}\right)\left\|\boldsymbol{\theta}_j - \boldsymbol{\theta}_m\right\|_2.\end{aligned}$$

For Γ_2, we have that

$$\begin{aligned}\Gamma_2 = & \sum_{(j,m)\in\mathcal{A}\setminus\mathcal{A}^{\mathrm{p}}} p'\left(\left\|\bar{\boldsymbol{\theta}}_j - \bar{\boldsymbol{\theta}}_m\right\|_2; \lambda_1\right) \left\|\bar{\boldsymbol{\theta}}_j - \bar{\boldsymbol{\theta}}_m\right\|_2^{-1} \left(\bar{\boldsymbol{\theta}}_j - \bar{\boldsymbol{\theta}}_m\right)^{\mathrm{T}} \left(\boldsymbol{\theta}_j - \breve{\boldsymbol{\theta}}_j\right) \\ & + \sum_{(m,j)\in\mathcal{A}\setminus\mathcal{A}^{\mathrm{p}}} p'\left(\left\|\bar{\boldsymbol{\theta}}_j - \bar{\boldsymbol{\theta}}_m\right\|_2; \lambda_1\right) \left\|\bar{\boldsymbol{\theta}}_j - \bar{\boldsymbol{\theta}}_m\right\|_2^{-1} \left(\bar{\boldsymbol{\theta}}_j - \bar{\boldsymbol{\theta}}_m\right)^{\mathrm{T}} \left(\boldsymbol{\theta}_j - \breve{\boldsymbol{\theta}}_j\right) \\ = & \sum_{(j,m)\in\mathcal{A}\setminus\mathcal{A}^{\mathrm{p}}} p'\left(\left\|\bar{\boldsymbol{\theta}}_j - \bar{\boldsymbol{\theta}}_m\right\|_2; \lambda_1\right) \left\|\bar{\boldsymbol{\theta}}_j - \bar{\boldsymbol{\theta}}_m\right\|_2^{-1} \left(\bar{\boldsymbol{\theta}}_j - \bar{\boldsymbol{\theta}}_m\right)^{\mathrm{T}} \left(\left(\boldsymbol{\theta}_j - \breve{\boldsymbol{\theta}}_j\right) - \left(\boldsymbol{\theta}_m - \breve{\boldsymbol{\theta}}_m\right)\right).\end{aligned}$$

Note that for $j, m \in \mathcal{T}_{k_2}^*$, we have that $\breve{\boldsymbol{\theta}}_j = \breve{\boldsymbol{\theta}}_m$, and

$$\left\|\bar{\boldsymbol{\theta}}_j - \bar{\boldsymbol{\theta}}_m\right\|_2^{-1} \left(\bar{\boldsymbol{\theta}}_j - \bar{\boldsymbol{\theta}}_m\right)^{\mathrm{T}} \left(\boldsymbol{\theta}_j - \boldsymbol{\theta}_m\right) = \left\|\boldsymbol{\theta}_j - \boldsymbol{\theta}_m\right\|_2.$$

Moreover,

$$\begin{aligned}\left\|\bar{\boldsymbol{\theta}}_j - \bar{\boldsymbol{\theta}}_m\right\|_2 & \leqslant \left\|\bar{\boldsymbol{\theta}}_j - \breve{\boldsymbol{\theta}}_j\right\|_2 + \left\|\breve{\boldsymbol{\theta}}_m - \bar{\boldsymbol{\theta}}_m\right\|_2 \leqslant \left\|\boldsymbol{\theta}_j - \breve{\boldsymbol{\theta}}_j\right\|_2 + \left\|\boldsymbol{\theta}_m - \breve{\boldsymbol{\theta}}_m\right\|_2 \\ & \leqslant \left\|\boldsymbol{\theta}_j - \widehat{\boldsymbol{\theta}}_j^0\right\|_2 + \left\|\widehat{\boldsymbol{\theta}}_j^0 - \breve{\boldsymbol{\theta}}_j\right\|_2 + \left\|\boldsymbol{\theta}_m - \widehat{\boldsymbol{\theta}}_m^0\right\|_2 + \left\|\widehat{\boldsymbol{\theta}}_m^0 - \breve{\boldsymbol{\theta}}_m\right\|_2 \leqslant 8\psi_n.\end{aligned}$$

Hence, for $j, m \in \mathcal{T}_{k_2}^*$,

$$p'\left(\left\|\bar{\boldsymbol{\theta}}_j - \bar{\boldsymbol{\theta}}_m\right\|_2; \lambda_1\right) \left\|\bar{\boldsymbol{\theta}}_j - \bar{\boldsymbol{\theta}}_m\right\|_2^{-1} \left(\bar{\boldsymbol{\theta}}_j - \bar{\boldsymbol{\theta}}_m\right)^{\mathrm{T}} \left(\boldsymbol{\theta}_j - \boldsymbol{\theta}_m\right) \geqslant p'\left(8\psi_n; \lambda_1\right) \left\|\boldsymbol{\theta}_j - \boldsymbol{\theta}_m\right\|_2.$$

Note that for $j \in \mathcal{T}_{k_2}^*$ and $m \in \mathcal{T}_{k_2'}^*$, we have that

$$\left\|\bar{\boldsymbol{\theta}}_j - \bar{\boldsymbol{\theta}}_m\right\|_2 \geqslant \left\|\boldsymbol{\theta}_j^* - \boldsymbol{\theta}_m^*\right\|_2 - \left\|\boldsymbol{\theta}_j^* - \bar{\boldsymbol{\theta}}_j\right\|_2 - \left\|\bar{\boldsymbol{\theta}}_m - \boldsymbol{\theta}_m^*\right\|_2 \geqslant d_n - 2C\phi_n > a\lambda_1,$$

hence, $p'\left(\left\|\bar{\boldsymbol{\theta}}_j - \bar{\boldsymbol{\theta}}_m\right\|_2; \lambda_1\right) = 0$. Then we have that

$$\Gamma_2 \geqslant \sum_{k_2=1}^{K_2} \sum_{j,m\in\mathcal{T}_{k_2}^*, (j,m)\in\mathcal{A}\setminus\mathcal{A}^{\mathrm{p}}} p'\left(8\psi_n; \lambda_1\right) \left\|\boldsymbol{\theta}_j - \boldsymbol{\theta}_m\right\|_2.$$

In a similar way, we have that

$$\Gamma_3 \geqslant \sum_{k_1=1}^{K_1} \sum_{j,m \in \mathcal{G}_{k_1}^*, (j,m) \in \mathcal{A} \setminus \mathcal{A}^{\mathrm{p}}} p'\left(4\psi_n; \lambda_2\right) \left\|\boldsymbol{\beta}_j - \boldsymbol{\beta}_m\right\|_2 .$$

In conclusion,

$$\begin{aligned} & Q\left(\boldsymbol{\beta}, \boldsymbol{\gamma}\right) - Q\left(\breve{\boldsymbol{\beta}}, \breve{\boldsymbol{\gamma}}\right) \\ \geqslant & \sum_{k_1=1}^{K_1} \sum_{j,m \in \mathcal{G}_{k_1}^*, (j,m) \in \mathcal{A} \setminus \mathcal{A}^{\mathrm{p}}} \left\{p'\left(4\psi_n; \lambda_2\right) - 2C_1 G_{\min}^{-1}\left(q^{\frac{1}{2}} + (\log n)^{\frac{1}{2}}\right)\right\} \left\|\boldsymbol{\beta}_j - \boldsymbol{\beta}_m\right\|_2 \\ & + \sum_{k_2=1}^{K_2} \sum_{j,m \in \mathcal{T}_{k_2}^*, (j,m) \in \mathcal{A} \setminus \mathcal{A}^{\mathrm{p}}} \left\{p'\left(8\psi_n; \lambda_1\right) - 2C_2 T_{\min}^{-1}\left(p^{\frac{1}{2}} + (\log n)^{\frac{1}{2}}\right)\right\} \left\|\boldsymbol{\theta}_j - \boldsymbol{\theta}_m\right\|_2 . \end{aligned}$$

Note that $\psi_n \to 0$, then we have $p'\left(4\psi_n; \lambda_2\right) \to \lambda_2$ and $p'\left(8\psi_n; \lambda_1\right) \to \lambda_1$. Moreover, since $\min\left\{\lambda_1, \lambda_2\right\} \gg \phi_n = (q+p)^{\frac{1}{2}} T_{\min}^{-\frac{1}{2}} (\log n)^{\frac{1}{2}} \gg (q+p)^{\frac{1}{2}} T_{\min}^{-1} (\log n)^{\frac{1}{2}} > (q+p)^{\frac{1}{2}} G_{\min}^{-1} (\log n)^{\frac{1}{2}}$. Hence, with probability at least $1 - 2n^{-1}$, for sufficiently large n, $Q\left(\boldsymbol{\beta}, \boldsymbol{\gamma}\right) \geqslant Q\left(\breve{\boldsymbol{\beta}}, \breve{\boldsymbol{\gamma}}\right)$ and (2) is proved. By (1) and (2), we have that with probability at least $1 - 4n^{-1}$, (*) holds and for any $\left(\boldsymbol{\beta}, \boldsymbol{\gamma}\right) \in \Theta \cap \Theta_n$, we have that $Q\left(\boldsymbol{\beta}, \boldsymbol{\gamma}\right) \geqslant Q\left(\breve{\boldsymbol{\beta}}, \breve{\boldsymbol{\gamma}}\right) \geqslant Q\left(\widehat{\boldsymbol{\beta}}^0, \widehat{\boldsymbol{\gamma}}^0\right)$, resulting in $\left(\widehat{\boldsymbol{\beta}}^0, \widehat{\boldsymbol{\gamma}}^0\right)$ as a local minimizer of $Q\left(\boldsymbol{\beta}, \boldsymbol{\gamma}\right)$ subject to $\boldsymbol{\beta} \in \mathcal{M}_1$ and $\boldsymbol{\gamma} \in \mathcal{M}_2$. Hence, Lemma 3 is proved.

References

[1] Phillippe Rigollet and Jan-Christian Hütter. High dimensional statistics. *Lecture notes for course 18S997*, 813(814):46, 2015.

B Details of the computational algorithm

Recall $\mathcal{L}(\boldsymbol{\beta}, \boldsymbol{\gamma}, \boldsymbol{\omega}, \boldsymbol{\eta}, \boldsymbol{v}, \boldsymbol{u})$ and (4.1)-(4.3) in the main text. In this section, we give the details of the deveriation of (4.4) and (4.5).

Define $\mathrm{vec}(\cdot)$ as the vectorization of matrices, specifically, for a $r_1 \times r_2$ matrix $\boldsymbol{A} = (\boldsymbol{A}_1, \cdots, \boldsymbol{A}_{r_2})$ of each column being a r_1-dimensional vector, the vectorization of $\boldsymbol{A}$ is $\mathrm{vec}(\boldsymbol{A}) = \left(\boldsymbol{A}_1^{\mathrm{T}}, \cdots, \boldsymbol{A}_{r_2}^{\mathrm{T}}\right)^{\mathrm{T}}$ which is a $r_1 r_2$-dimensional vector. For sample data $\boldsymbol{X} = (\boldsymbol{X}_1, \cdots, \boldsymbol{X}_n)$ and $\boldsymbol{Z} = (\boldsymbol{Z}_1, \cdots, \boldsymbol{Z}_n)$, the vectorization $\mathrm{vec}\left(\left(\boldsymbol{X}^{\mathrm{T}}, \boldsymbol{Z}^{\mathrm{T}}\right)^{\mathrm{T}}\right)$ is a $n(q+p)$-dimensional vector. Similarly, the parameters matrix and Lagrange multipliers can be also expressed as the form of vectorization. By discarding terms independent of $(\boldsymbol{\beta}, \boldsymbol{\gamma}, \boldsymbol{\omega}, \boldsymbol{\eta})$, the augmented Lagrangian function $\mathcal{L}(\boldsymbol{\beta}, \boldsymbol{\gamma}, \boldsymbol{\omega}, \boldsymbol{\eta}, \boldsymbol{v}, \boldsymbol{u})$ can be reformulated as

$$
\begin{aligned}
\widetilde{\mathcal{L}} =& \frac{1}{2} \sum_{i=1}^{n} \left(\left\| \left(\boldsymbol{X}_i^{\mathrm{T}}, \boldsymbol{Z}_i^{\mathrm{T}}\right)^{\mathrm{T}} - \left(\boldsymbol{\beta}_i^{\mathrm{T}}, \boldsymbol{\gamma}_i^{\mathrm{T}}\right)^{\mathrm{T}} \right\|_2^2 \right) \\
&+ \sum_{(j,m)\in\mathcal{A}} p\left(\left(\|\boldsymbol{\omega}_{jm}\|_2^2 + \|\boldsymbol{\eta}_{jm}\|_2^2 \right)^{\frac{1}{2}}; \lambda_1 \right) + \sum_{(j,m)\in\mathcal{A}} p\left(\|\boldsymbol{\omega}_{jm}\|_2; \lambda_2 \right) \\
&+ \frac{\vartheta}{2} \sum_{(j,m)\in\mathcal{A}} \left\| \left(\boldsymbol{\beta}_j^{\mathrm{T}} - \boldsymbol{\beta}_m^{\mathrm{T}}, \boldsymbol{\gamma}_j^{\mathrm{T}} - \boldsymbol{\gamma}_m^{\mathrm{T}}\right)^{\mathrm{T}} - \left(\boldsymbol{\omega}_{jm}^{\mathrm{T}} - \vartheta^{-1}\boldsymbol{v}_{jm}^{\mathrm{T}}, \boldsymbol{\eta}_{jm}^{\mathrm{T}} - \vartheta^{-1}\boldsymbol{u}_{jm}^{\mathrm{T}}\right)^{\mathrm{T}} \right\|_2^2 .
\end{aligned}
$$

Note that $\boldsymbol{\beta} \in \mathcal{M}_1$ and $\boldsymbol{\gamma} \in \mathcal{M}_2$. There exists unique matrices $\widetilde{\boldsymbol{\beta}} \in \mathbb{R}^q \times \mathbb{R}^K$ and $\widetilde{\boldsymbol{\gamma}} \in \mathbb{R}^p \times \mathbb{R}^K$ such that $\mathrm{vec}\left(\left(\boldsymbol{\beta}^{\mathrm{T}}, \boldsymbol{\gamma}^{\mathrm{T}}\right)^{\mathrm{T}}\right) = \boldsymbol{J}\mathrm{vec}\left(\left(\widetilde{\boldsymbol{\beta}}^{\mathrm{T}}, \widetilde{\boldsymbol{\gamma}}^{\mathrm{T}}\right)^{\mathrm{T}}\right)$. By discarding terms independent of $(\boldsymbol{\beta}, \boldsymbol{\gamma})$, the optimization problem (4.1) is equivalent to minimizing that

$$
\begin{aligned}
f\left(\widetilde{\boldsymbol{\beta}}, \widetilde{\boldsymbol{\gamma}}\right) =& \frac{1}{2} \left\| \mathrm{vec}\left(\left(\boldsymbol{X}^{\mathrm{T}}, \boldsymbol{Z}^{\mathrm{T}}\right)^{\mathrm{T}}\right) - \boldsymbol{J}\mathrm{vec}\left(\left(\widetilde{\boldsymbol{\beta}}^{\mathrm{T}}, \widetilde{\boldsymbol{\gamma}}^{\mathrm{T}}\right)^{\mathrm{T}}\right) \right\|_2^2 \\
&+ \frac{\vartheta}{2} \left\| \boldsymbol{H}\boldsymbol{J}\mathrm{vec}\left(\left(\widetilde{\boldsymbol{\beta}}^{\mathrm{T}}, \widetilde{\boldsymbol{\gamma}}^{\mathrm{T}}\right)^{\mathrm{T}}\right) - \mathrm{vec}\left(\left(\boldsymbol{\omega}^{(t)\mathrm{T}} - \vartheta^{-1}\boldsymbol{v}^{(t)\mathrm{T}}, \boldsymbol{\eta}^{(t)\mathrm{T}} - \vartheta^{-1}\boldsymbol{u}^{(t)\mathrm{T}}\right)^{\mathrm{T}}\right) \right\|_2^2 .
\end{aligned}
$$

Minimizing $f\left(\widetilde{\boldsymbol{\beta}}, \widetilde{\boldsymbol{\gamma}}\right)$ is a standard quadratic convex optimization problem and the global

solution is unique. Hence, the updates for $\left(\widetilde{\boldsymbol{\beta}}^{(t+1)}, \widetilde{\boldsymbol{\gamma}}^{(t+1)}\right)$ satisfy

$$\begin{aligned}
&\left(\boldsymbol{J}^{\mathrm{T}}\boldsymbol{J} + \vartheta \boldsymbol{J}^{\mathrm{T}}\boldsymbol{H}^{\mathrm{T}}\boldsymbol{H}\boldsymbol{J}\right) \mathrm{vec}\left(\left(\widetilde{\boldsymbol{\beta}}^{(t+1)\mathrm{T}}, \widetilde{\boldsymbol{\gamma}}^{(t+1)\mathrm{T}}\right)^{\mathrm{T}}\right) \\
=&\boldsymbol{J}^{\mathrm{T}}\mathrm{vec}\left(\left(\boldsymbol{X}^{\mathrm{T}}, \boldsymbol{Z}^{\mathrm{T}}\right)^{\mathrm{T}}\right) + \boldsymbol{J}^{\mathrm{T}}\boldsymbol{H}^{\mathrm{T}}\mathrm{vec}\left(\left(\vartheta\boldsymbol{\omega}^{(t)\mathrm{T}} - \boldsymbol{v}^{(t)\mathrm{T}}, \vartheta\boldsymbol{\eta}^{(t)\mathrm{T}} - \boldsymbol{u}^{(t)\mathrm{T}}\right)^{\mathrm{T}}\right).
\end{aligned}$$

It is noted that $\boldsymbol{J}^{\mathrm{T}}\boldsymbol{J} + \vartheta \boldsymbol{J}^{\mathrm{T}}\boldsymbol{H}^{\mathrm{T}}\boldsymbol{H}\boldsymbol{J}$ is a $K(q+p) \times K(q+p)$ matrix, calculation of its inverse matrix costs huge computational expenses in high-dimensional situation. By some linear algebra knowledge, we aim to find out the special structure of such defined matrix and obtain its inverse simply. By properties of Kronecker product, we have that

$$\begin{aligned}
&\boldsymbol{J} = \boldsymbol{L} \otimes \boldsymbol{I}_{q+p}, \quad \boldsymbol{J}^{\mathrm{T}}\boldsymbol{J} = \left(\boldsymbol{L}^{\mathrm{T}}\boldsymbol{L}\right) \otimes \boldsymbol{I}_{q+p}, \\
&\boldsymbol{H}\boldsymbol{J} = (\boldsymbol{D}\boldsymbol{L}) \otimes \boldsymbol{I}_{q+p}, \quad \boldsymbol{J}^{\mathrm{T}}\boldsymbol{H}^{\mathrm{T}}\boldsymbol{H}\boldsymbol{J} = \left(\boldsymbol{L}^{\mathrm{T}}\boldsymbol{D}^{\mathrm{T}}\boldsymbol{D}\boldsymbol{L}\right) \otimes \boldsymbol{I}_{q+p}, \\
&\boldsymbol{J}^{\mathrm{T}}\boldsymbol{J} + \vartheta \boldsymbol{J}^{\mathrm{T}}\boldsymbol{H}^{\mathrm{T}}\boldsymbol{H}\boldsymbol{J} = \left(\boldsymbol{L}^{\mathrm{T}}\boldsymbol{L} + \vartheta \boldsymbol{L}^{\mathrm{T}}\boldsymbol{D}^{\mathrm{T}}\boldsymbol{D}\boldsymbol{L}\right) \otimes \boldsymbol{I}_{q+p}.
\end{aligned}$$

By properties of Kronecker product with vectorization ($\mathrm{vec}\,(\boldsymbol{A}\boldsymbol{B}) = \left(\boldsymbol{B}^{\mathrm{T}} \otimes \boldsymbol{I}\right)\mathrm{vec}\,(\boldsymbol{A})$), the updates for $\left(\widetilde{\boldsymbol{\beta}}^{(t+1)}, \widetilde{\boldsymbol{\gamma}}^{(t+1)}\right)$ satisfy

$$\begin{aligned}
&\left(\widetilde{\boldsymbol{\beta}}^{(t+1)\mathrm{T}}, \widetilde{\boldsymbol{\gamma}}^{(t+1)\mathrm{T}}\right)^{\mathrm{T}} \left(\boldsymbol{L}^{\mathrm{T}}\boldsymbol{L} + \vartheta \boldsymbol{L}^{\mathrm{T}}\boldsymbol{D}^{\mathrm{T}}\boldsymbol{D}\boldsymbol{L}\right) \\
=&\left(\boldsymbol{X}^{\mathrm{T}}, \boldsymbol{Z}^{\mathrm{T}}\right)^{\mathrm{T}}\boldsymbol{L} + \left(\vartheta\boldsymbol{\omega}^{(t)\mathrm{T}} - \boldsymbol{v}^{(t)\mathrm{T}}, \vartheta\boldsymbol{\eta}^{(t)\mathrm{T}} - \boldsymbol{u}^{(t)\mathrm{T}}\right)^{\mathrm{T}} \boldsymbol{D}\boldsymbol{L}.
\end{aligned}$$

Since $\boldsymbol{L}^{\mathrm{T}}\boldsymbol{L} + \vartheta \boldsymbol{L}^{\mathrm{T}}\boldsymbol{D}^{\mathrm{T}}\boldsymbol{D}\boldsymbol{L}$ is a $K \times K$ matrix, and K is much smaller than $K(q+p)$ to some extent, hence its inverse matrix is efficient to calculate. The updates for $\left(\widetilde{\boldsymbol{\beta}}^{(t+1)}, \widetilde{\boldsymbol{\gamma}}^{(t+1)}\right)$ are

$$\begin{aligned}
&\left(\widetilde{\boldsymbol{\beta}}^{(t+1)\mathrm{T}}, \widetilde{\boldsymbol{\gamma}}^{(t+1)\mathrm{T}}\right)^{\mathrm{T}} \\
=&\left\{\left(\boldsymbol{X}^{\mathrm{T}}, \boldsymbol{Z}^{\mathrm{T}}\right)^{\mathrm{T}} + \left(\vartheta\boldsymbol{\omega}^{(t)\mathrm{T}} - \boldsymbol{v}^{(t)\mathrm{T}}, \vartheta\boldsymbol{\eta}^{(t)\mathrm{T}} - \boldsymbol{u}^{(t)\mathrm{T}}\right)^{\mathrm{T}} \boldsymbol{D}\right\} \boldsymbol{L}\left(\boldsymbol{L}^{\mathrm{T}}\boldsymbol{L} + \vartheta \boldsymbol{L}^{\mathrm{T}}\boldsymbol{D}^{\mathrm{T}}\boldsymbol{D}\boldsymbol{L}\right)^{-1}.
\end{aligned}$$

Then, the updates for $\left(\boldsymbol{\beta}^{(t+1)}, \boldsymbol{\gamma}^{(t+1)}\right)$ are

$$\operatorname{vec}\left(\left(\boldsymbol{\beta}^{(t+1)\mathrm{T}}, \boldsymbol{\gamma}^{(t+1)\mathrm{T}}\right)^{\mathrm{T}}\right) = \boldsymbol{J}\operatorname{vec}\left(\left(\widetilde{\boldsymbol{\beta}}^{(t+1)\mathrm{T}}, \widetilde{\boldsymbol{\gamma}}^{(t+1)\mathrm{T}}\right)^{\mathrm{T}}\right),$$

When there is no prior information, that is, $\mathcal{A}^{\mathrm{P}} = \emptyset$. Without any information, the matrix $\boldsymbol{L} = \boldsymbol{I}_n$ and $K = n$. By the definition of matrix $\boldsymbol{D}$, it holds that $\boldsymbol{I}_n + \vartheta \boldsymbol{D}^{\mathrm{T}}\boldsymbol{D} = (1 + n\vartheta)\,\boldsymbol{I}_n - \vartheta \mathbf{1}_n \mathbf{1}_n^{\mathrm{T}}$, where $\mathbf{1}_n$ is a n-dimensional vector whose all elements are 1. By Sherman-Morrison formula, it holds that $\left((1 + n\vartheta)\,\boldsymbol{I}_n - \vartheta \mathbf{1}_n \mathbf{1}_n^{\mathrm{T}}\right)^{-1} = (1 + n\vartheta)^{-1}\left(\boldsymbol{I}_n + \vartheta \mathbf{1}_n \mathbf{1}_n^{\mathrm{T}}\right)$. Hence,

$$\begin{aligned}&\left(\boldsymbol{\beta}^{(t+1)\mathrm{T}}, \boldsymbol{\gamma}^{(t+1)\mathrm{T}}\right)^{\mathrm{T}} \\ =&\frac{1}{1+n\vartheta}\left\{\left(\boldsymbol{X}^{\mathrm{T}}, \boldsymbol{Z}^{\mathrm{T}}\right)^{\mathrm{T}} + \left(\vartheta \boldsymbol{\omega}^{(t)\mathrm{T}} - \boldsymbol{v}^{(t)\mathrm{T}}, \vartheta \boldsymbol{\eta}^{(t)\mathrm{T}} - \boldsymbol{u}^{(t)\mathrm{T}}\right)^{\mathrm{T}} \boldsymbol{D}\right\}\left(\boldsymbol{I}_n + \vartheta \mathbf{1}_n \mathbf{1}_n^{\mathrm{T}}\right).\end{aligned}$$

Let $\boldsymbol{\omega}_{jm}^{*(t)} = \boldsymbol{\beta}_j^{(t+1)} - \boldsymbol{\beta}_m^{(t+1)} + \vartheta^{-1}\boldsymbol{v}_{jm}^{(t)}$ and $\boldsymbol{\eta}_{jm}^{*(t)} = \boldsymbol{\gamma}_j^{(t+1)} - \boldsymbol{\gamma}_m^{(t+1)} + \vartheta^{-1}\boldsymbol{u}_{jm}^{(t)}$. By discarding the terms independent of $(\boldsymbol{\omega}, \boldsymbol{\eta})$, the optimization problem (4.2) is equivalent to minimizing

$$\frac{\vartheta}{2}\left\|\left(\boldsymbol{\omega}_{jm}^{\mathrm{T}}, \boldsymbol{\eta}_{jm}^{\mathrm{T}}\right)^{\mathrm{T}} - \left(\boldsymbol{\omega}_{jm}^{*(t)\mathrm{T}}, \boldsymbol{\eta}_{jm}^{*(t)\mathrm{T}}\right)^{\mathrm{T}}\right\|_2^2 + p\left(\left(\|\boldsymbol{\omega}_{jm}\|_2^2 + \|\boldsymbol{\eta}_{jm}\|_2^2\right)^{\frac{1}{2}}; \lambda_1\right) + p\left(\|\boldsymbol{\omega}_{jm}\|_2; \lambda_2\right),$$

with respect to $\left(\boldsymbol{\omega}_{jm}^{\mathrm{T}}, \boldsymbol{\eta}_{jm}^{\mathrm{T}}\right)^{\mathrm{T}}$. The penalty $p(\cdot;\lambda)$ is MCP with a fixed parameter $a > \vartheta^{-1}$. Denote $\boldsymbol{\zeta}_{jm} = \left(\boldsymbol{\omega}_{jm}^{\mathrm{T}}, \boldsymbol{\eta}_{jm}^{\mathrm{T}}\right)^{\mathrm{T}}$ and $(s)_+ = s\mathbb{I}(s > 0)$. The solution of (4.2) is $S\left(\boldsymbol{\omega}_{jm}^{*(t)}, \boldsymbol{\eta}_{jm}^{*(t)}\right)$, which can be expressed by the following discussion.

Case 1. If $\left\|\boldsymbol{\zeta}_{jm}^{*(t)}\right\|_2 > a\lambda_1$ and $\left\|\boldsymbol{\omega}_{jm}^{*(t)}\right\|_2 > a\lambda_2$, then $\boldsymbol{\omega}_{jm}^{(t+1)} = \boldsymbol{\omega}_{jm}^{*(t)}$ and $\boldsymbol{\eta}_{jm}^{(t+1)} = \boldsymbol{\eta}_{jm}^{*(t)}$.

Case 2. If $\left\|\boldsymbol{\zeta}_{jm}^{*(t)}\right\|_2 \leqslant a\lambda_1$ and $\frac{\left(1-\lambda_1\left(\vartheta\left\|\boldsymbol{\zeta}_{jm}^{*(t)}\right\|_2\right)^{-1}\right)_+}{1-(a\vartheta)^{-1}}\left\|\boldsymbol{\omega}_{jm}^{*(t)}\right\|_2 > a\lambda_2$, then,

$$\boldsymbol{\omega}_{jm}^{(t+1)} = \frac{\left(1-\lambda_1\left(\vartheta\left\|\boldsymbol{\zeta}_{jm}^{*(t)}\right\|_2\right)^{-1}\right)_+}{1-(a\vartheta)^{-1}}\boldsymbol{\omega}_{jm}^{*(t)}, \quad \boldsymbol{\eta}_{jm}^{(t+1)} = \frac{\left(1-\lambda_1\left(\vartheta\left\|\boldsymbol{\zeta}_{jm}^{*(t)}\right\|_2\right)^{-1}\right)_+}{1-(a\vartheta)^{-1}}\boldsymbol{\eta}_{jm}^{*(t)}.$$

Case 3. If $\left\|\boldsymbol{\eta}_{jm}^{*(t)}\right\|_2^2 + \left(\frac{\left(1-\lambda_2\left(\vartheta\left\|\boldsymbol{\omega}_{jm}^{*(t)}\right\|_2\right)^{-1}\right)_+}{1-(a\vartheta)^{-1}}\right)^2 \left\|\boldsymbol{\omega}_{jm}^{*(t)}\right\|_2^2 > (a\lambda_1)^2$ and $\left\|\boldsymbol{\omega}_{jm}^{*(t)}\right\|_2 \leqslant a\lambda_2$, then,

$$\boldsymbol{\omega}_{jm}^{(t+1)} = \frac{\left(1-\lambda_2\left(\vartheta\left\|\boldsymbol{\omega}_{jm}^{*(t)}\right\|_2\right)^{-1}\right)_+}{1-(a\vartheta)^{-1}}\boldsymbol{\omega}_{jm}^{*(t)}, \quad \boldsymbol{\eta}_{jm}^{(t+1)} = \boldsymbol{\eta}_{jm}^{*(t)}.$$

Other cases. Using the local quadratic approximation technique to obtain an explicit solution (Here, to avoid 0 appearing on the denominator, we can make a very small disturbance on $\left\|\boldsymbol{\zeta}_{jm}^{(t)}\right\|_2$ and $\left\|\boldsymbol{\omega}_{jm}^{(t)}\right\|_2$ in computation, such as 10^{-7}), then,

$$\boldsymbol{\omega}_{jm}^{(t+1)} = \left(1 + \frac{p'\left(\left\|\boldsymbol{\zeta}_{jm}^{(t)}\right\|_2; \lambda_1\right)}{\vartheta\left\|\boldsymbol{\zeta}_{jm}^{(t)}\right\|_2} + \frac{p'\left(\left\|\boldsymbol{\omega}_{jm}^{(t)}\right\|_2; \lambda_2\right)}{\vartheta\left\|\boldsymbol{\omega}_{jm}^{(t)}\right\|_2}\right)^{-1}\boldsymbol{\omega}_{jm}^{*(t)},$$

$$\boldsymbol{\eta}_{jm}^{(t+1)} = \left(1 + \frac{p'\left(\left\|\boldsymbol{\zeta}_{jm}^{(t)}\right\|_2; \lambda_1\right)}{\vartheta\left\|\boldsymbol{\zeta}_{jm}^{(t)}\right\|_2}\right)^{-1}\boldsymbol{\eta}_{jm}^{*(t)}.$$

C Additional numerical results

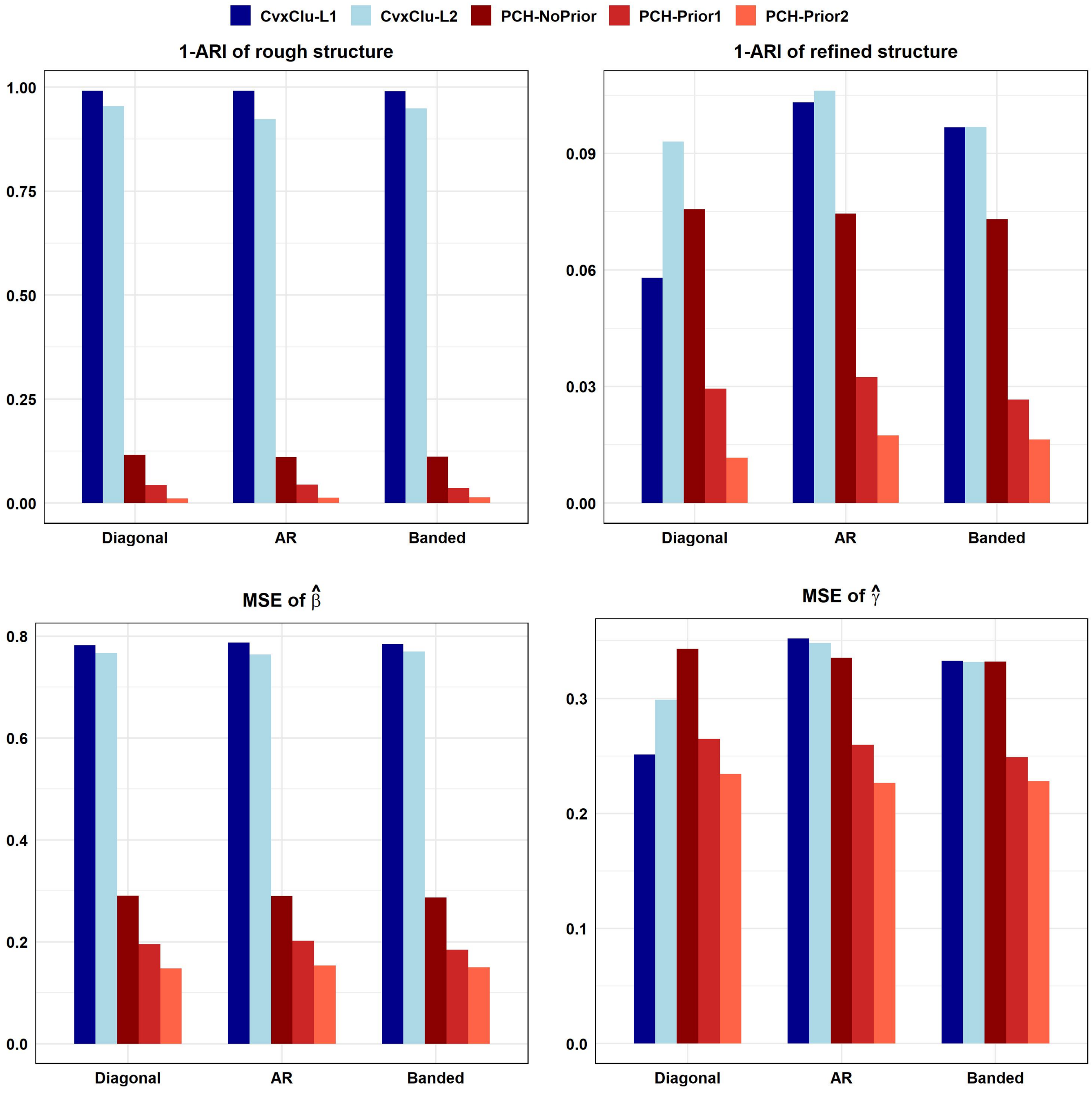

Figure S1: Simulation results with Simulation 1 and $\mu_1 = 1.2$. In each subfigure, horizontal axis displays our proposed methods and alternatives with three different covariance matrices, and longitudinal axis displays the mean of corresponding measurement values under 100 simulated replicates. The top-left subfigure displays $1 - \text{ARI}$ of rough clustering structure, the top-right subfigure displays $1 - \text{ARI}$ of refined clustering structure, the bottom-left subfigure displays MSE of $\hat{\boldsymbol{\beta}}$, and the bottom-right subfigure displays MSE of $\hat{\boldsymbol{\gamma}}$.

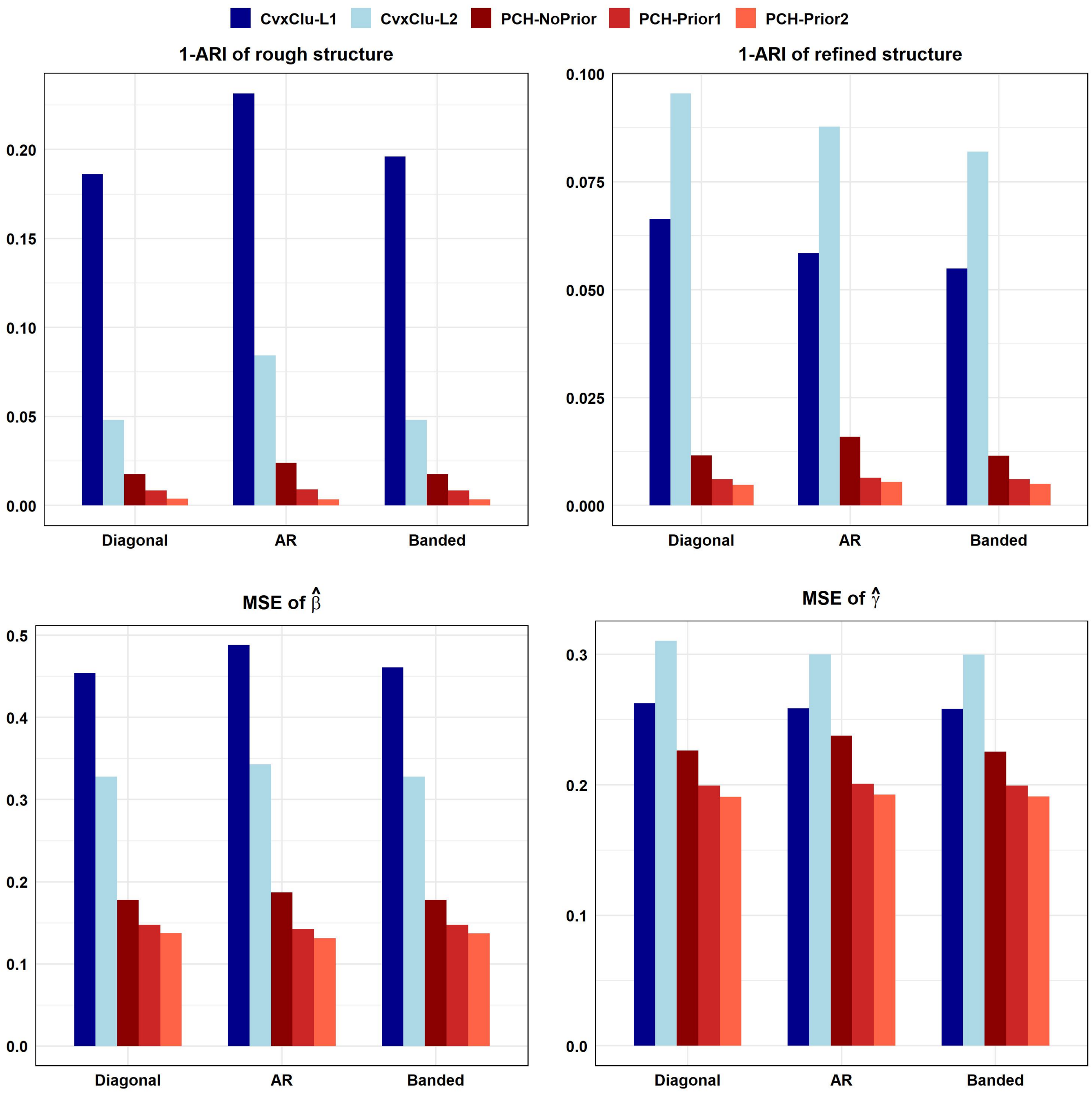

Figure S2: Simulation results with Simulation 1 and $\mu_2 = 1.6$. In each subfigure, horizontal axis displays our proposed methods and alternatives with three different covariance matrices, and longitudinal axis displays the mean of corresponding measurement values under 100 simulated replicates. The top-left subfigure displays $1 - \text{ARI}$ of rough clustering structure, the top-right subfigure displays $1 - \text{ARI}$ of refined clustering structure, the bottom-left subfigure displays MSE of $\hat{\boldsymbol{\beta}}$, and the bottom-right subfigure displays MSE of $\hat{\boldsymbol{\gamma}}$.

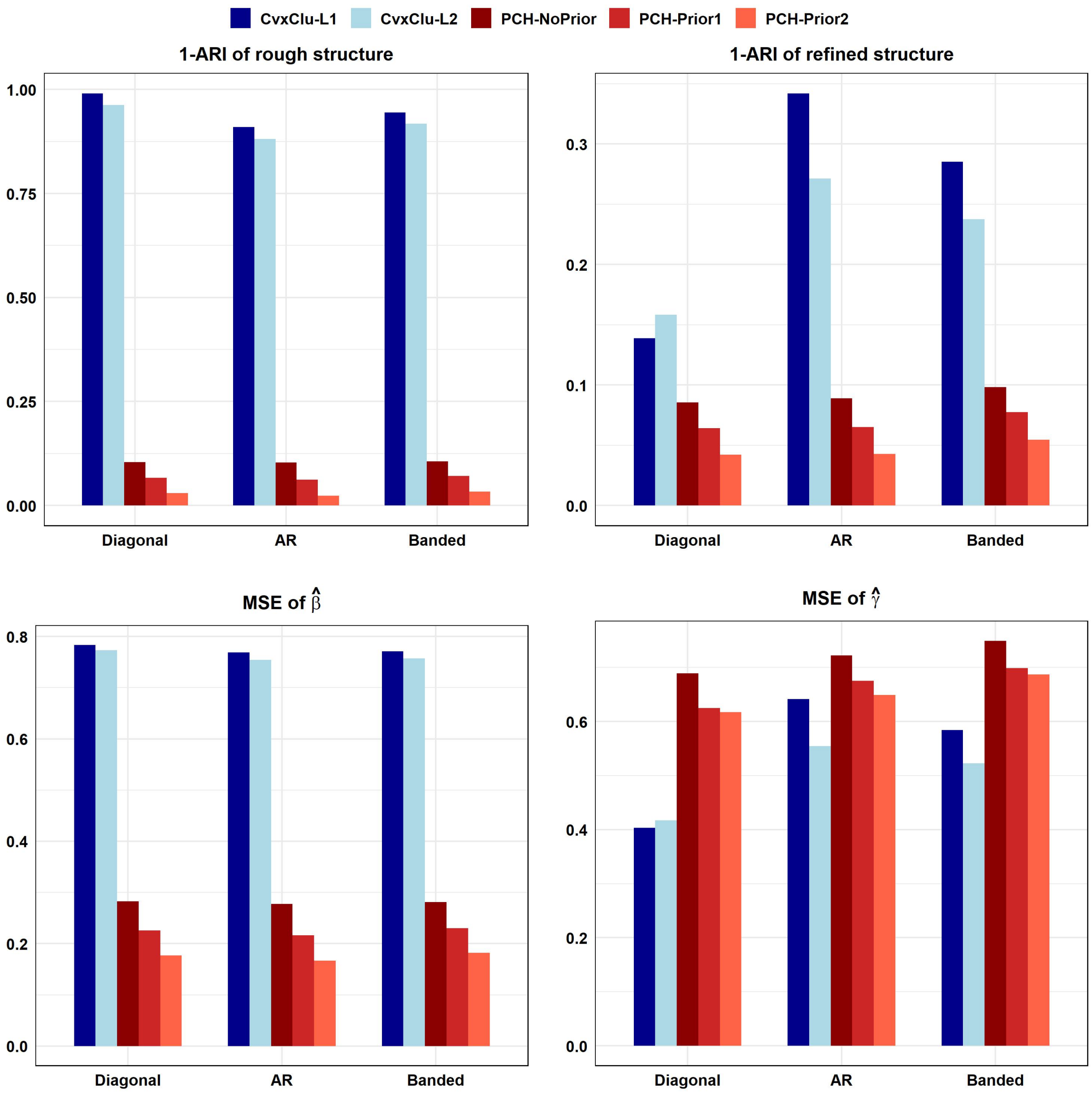

Figure S3: Simulation results with Simulation 2 and $\mu_1 = 1.2$. In each subfigure, horizontal axis displays our proposed methods and alternatives with three different covariance matrices, and longitudinal axis displays the mean of corresponding measurement values under 100 simulated replicates. The top-left subfigure displays $1 - \text{ARI}$ of rough clustering structure, the top-right subfigure displays $1 - \text{ARI}$ of refined clustering structure, the bottom-left subfigure displays MSE of $\hat{\boldsymbol{\beta}}$, and the bottom-right subfigure displays MSE of $\hat{\boldsymbol{\gamma}}$.

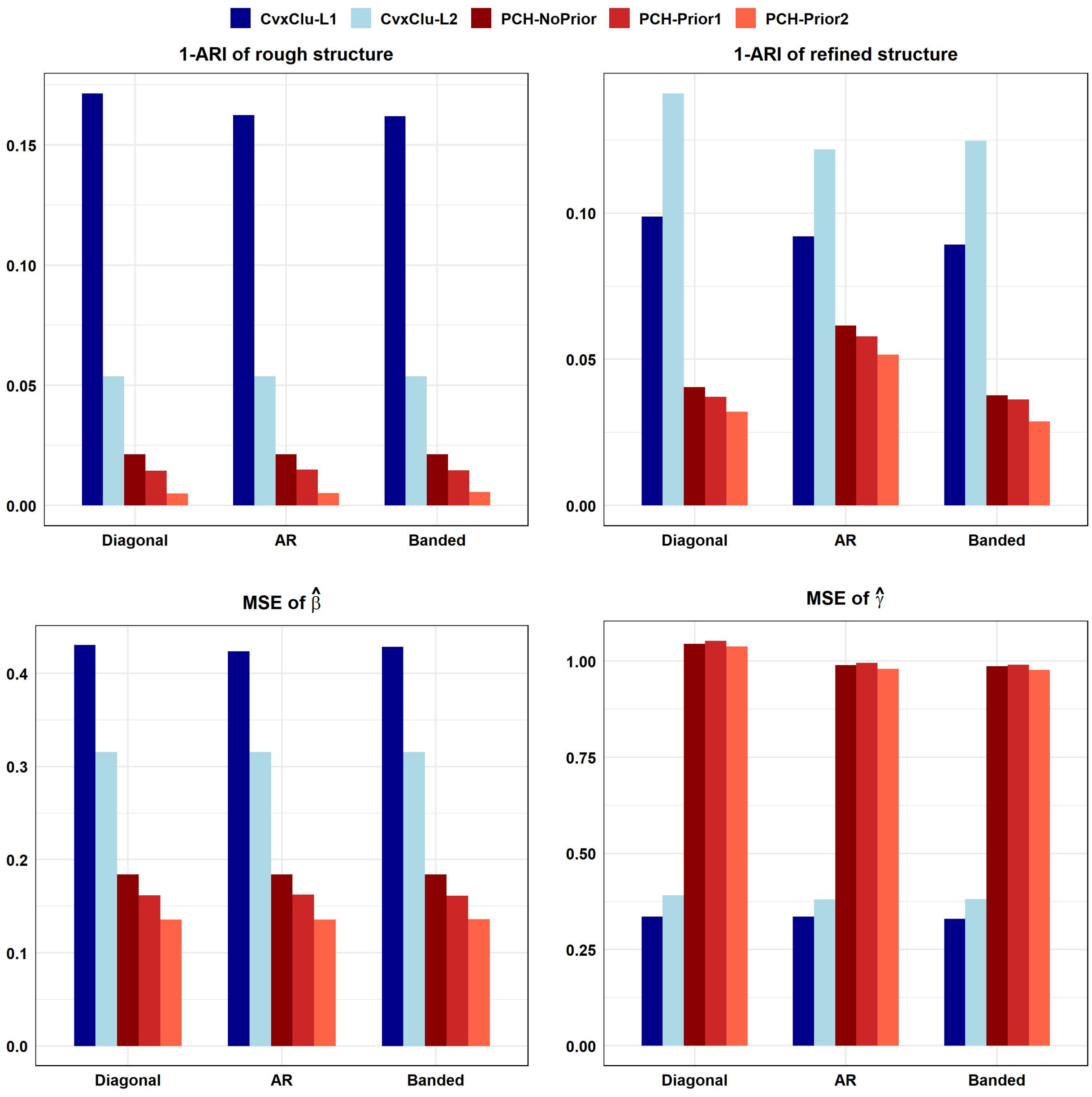

Figure S4: Simulation results with Simulation 2 and $\mu_2 = 1.6$. In each subfigure, horizontal axis displays our proposed methods and alternatives with three different covariance matrices, and longitudinal axis displays the mean of corresponding measurement values under 100 simulated replicates. The top-left subfigure displays $1 - \text{ARI}$ of rough clustering structure, the top-right subfigure displays $1 - \text{ARI}$ of refined clustering structure, the bottom-left subfigure displays MSE of $\hat{\boldsymbol{\beta}}$, and the bottom-right subfigure displays MSE of $\hat{\boldsymbol{\gamma}}$.

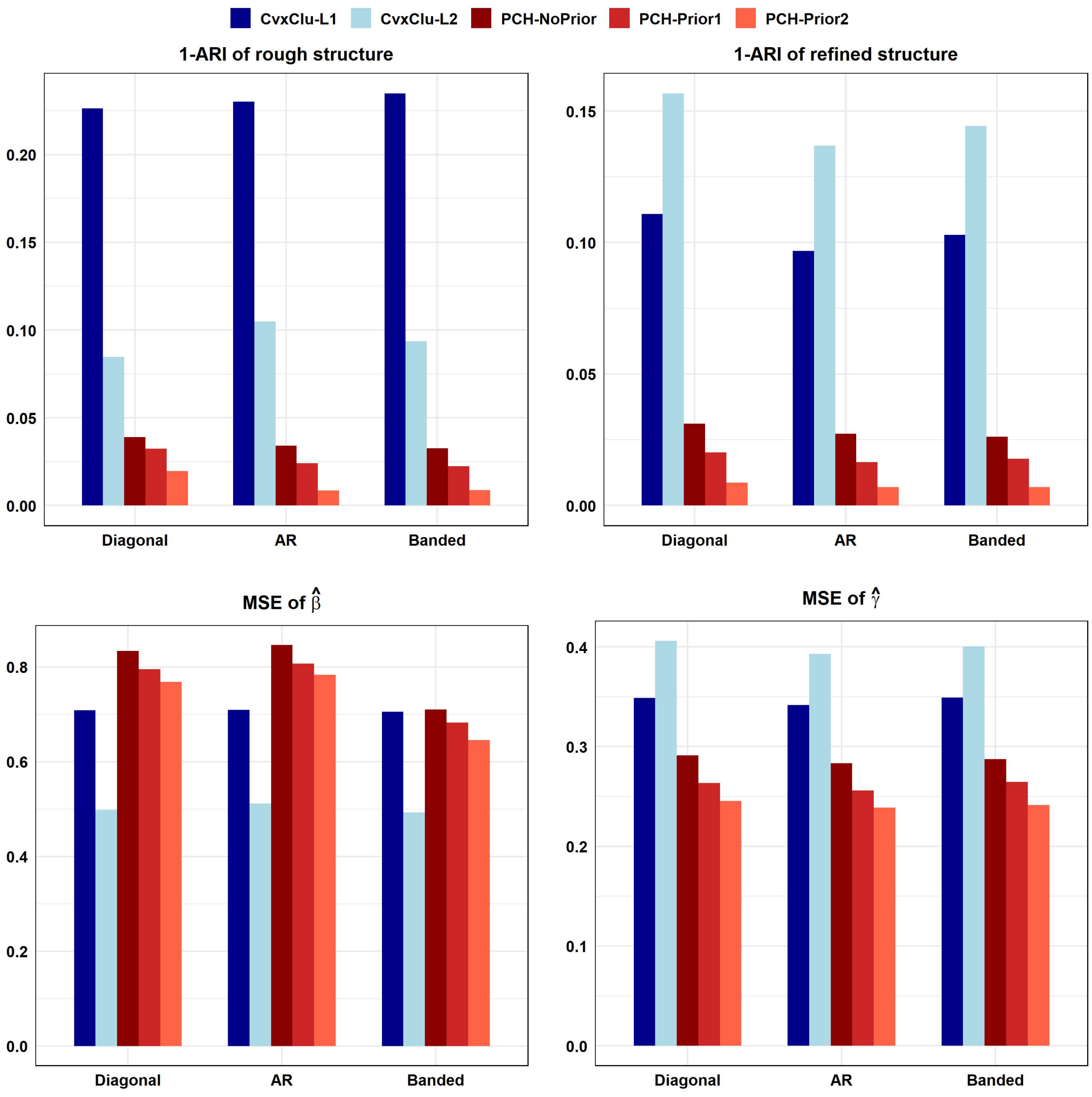

Figure S5: Simulation results with Simulation 3 and $\mu_2 = 1.6$. In each subfigure, horizontal axis displays our proposed methods and alternatives with three different covariance matrices, and longitudinal axis displays the mean of corresponding measurement values under 100 simulated replicates. The top-left subfigure displays $1 - \text{ARI}$ of rough clustering structure, the top-right subfigure displays $1 - \text{ARI}$ of refined clustering structure, the bottom-left subfigure displays MSE of $\hat{\boldsymbol{\beta}}$, and the bottom-right subfigure displays MSE of $\hat{\boldsymbol{\gamma}}$.

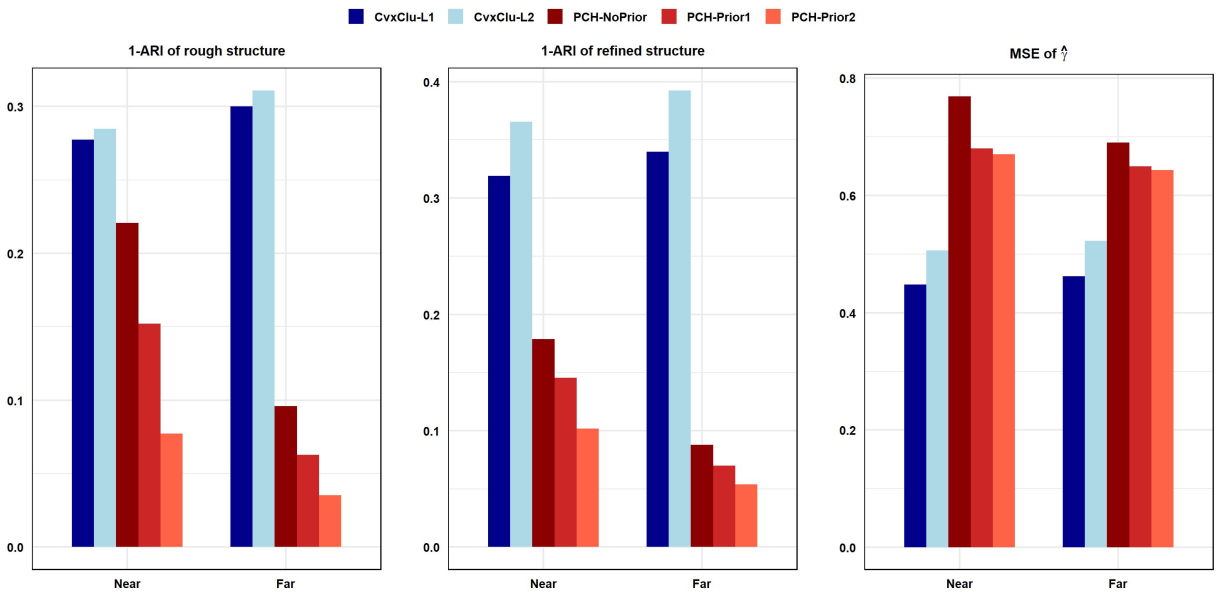

Figure S6: Simulation results with Simulation 5 in two half-moon clusters case. In each subfigure, horizontal axis displays our proposed methods and alternatives with near and far centers, and longitudinal axis displays the mean of corresponding measurement values under 100 simulated replicates. The left subfigure displays $1-\text{ARI}$ of rough clustering structure, the middle subfigure displays $1-\text{ARI}$ of refined clustering structure, and the right subfigure displays MSE of $\hat{\boldsymbol{\gamma}}$.

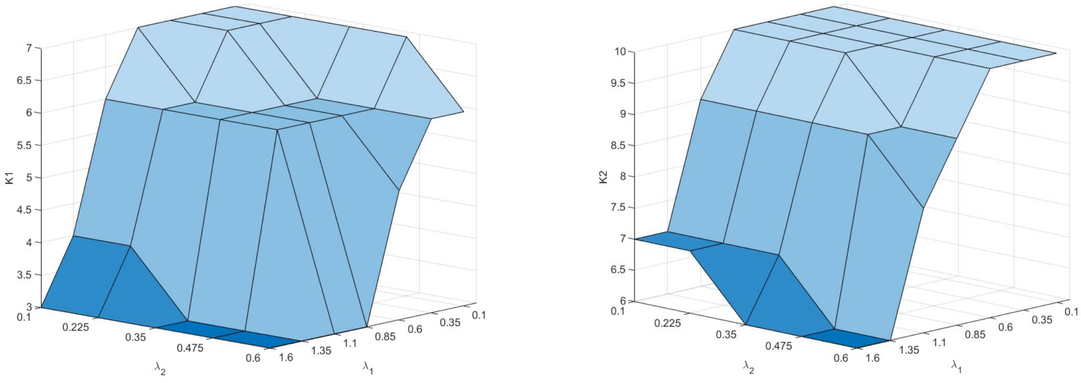

Figure S7: Analysis of one simulated data. The true numbers of rough and refined clusters are 3 and 6. The left subfigure displays the path of $\widehat{K}_1(\lambda_1, \lambda_2)$ in terms of λ_1 and λ_2. The right subfigure displays the path of $\widehat{K}_2(\lambda_1, \lambda_2)$ in terms of λ_1 and λ_2.

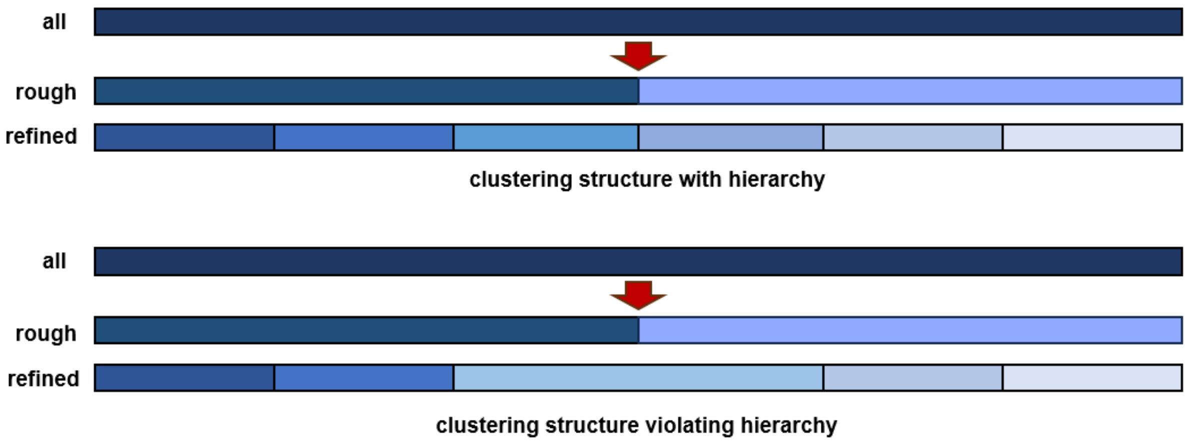

Figure S8: Additional exploration when the clustering hierarchy is violated. The top subfigure displays the clustering scheme in Simulation 2, where each rough cluster further groups into three refined clusters and holds hierarchy. The bottom subfigure displays the clustering scheme with $(K_1, K_2) = (2, 5)$ violating hierarchy, where some subjects from different rough clusters are grouped into the same refined cluster.

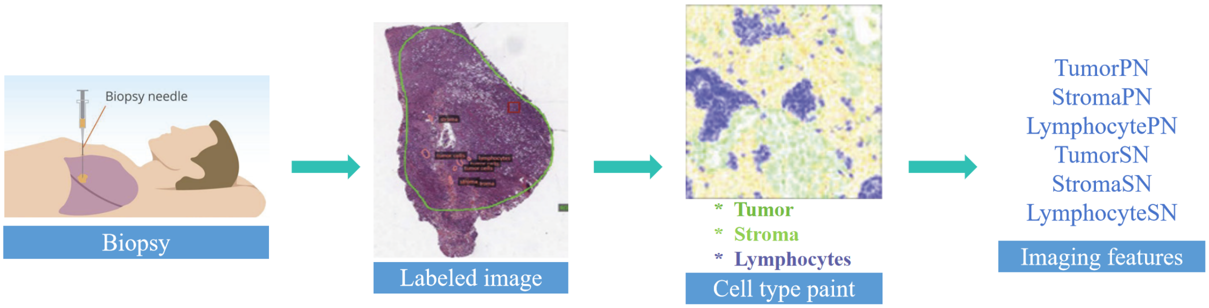

Figure S9: Analysis of LUAD data. Pipelines for extracting six clinical imaging features. LymphocytesPN: Perimeter of lymphocyte cell region/square root of image size. StromaPN: Perimeter of stromal cell region/square root of image size. TumorPN: Perimeter of tumor cell region/square root of image size. LymphocytesSN: Size of lymphocyte cell region/image size. StromaSN: Size of stromal cell region/image size. TumorSN: Size of tumor cell region/image size.

Table S1: Simulation results with $(K_1, K_2) = (2, 4)$ in Gaussian clusters case. Simulation results include the mean and standard deviation (SD) of ARI of rough and refined clustering structure, MSE of $\hat{\boldsymbol{\beta}}$, and MSE of $\hat{\boldsymbol{\gamma}}$ under 100 simulated replicates with three covariance structures and two distances between cluster centers.

			**Rough Structure**				**Refined Structure**			
			**ARI**		**MSE of $\hat{\boldsymbol{\beta}}$**		**ARI**		**MSE of $\hat{\boldsymbol{\gamma}}$**	
		Methods	Mean	SD	Mean	SD	Mean	SD	Mean	SD
**Diagonal**	$\mu_1 = 1.2$	CvxClu-L_1	0.0093	0.0936	0.7824	0.0445	0.9421	0.0288	0.2511	0.0415
		CvxClu-L_2	0.0461	0.2005	0.7669	0.0901	0.9070	0.0371	0.2988	0.0452
		PCH-NoPrior	0.8846	0.0596	0.2909	0.0696	0.9244	0.0401	0.3430	0.1380
		PCH-Prior1	0.9567	0.0397	0.1954	0.0589	0.9706	0.0251	0.2648	0.1287
		PCH-Prior2	0.9893	0.0194	0.1476	0.0368	0.9884	0.0171	0.2342	0.1298
	$\mu_2 = 1.6$	CvxClu-L_1	0.8138	0.3576	0.4544	0.3023	0.9336	0.0292	0.2626	0.0381
		CvxClu-L_2	0.9519	0.1009	0.3281	0.1815	0.9045	0.0350	0.3104	0.0440
		PCH-NoPrior	0.9824	0.0218	0.1782	0.0674	0.9884	0.0147	0.2263	0.0558
		PCH-Prior1	0.9917	0.0158	0.1475	0.0486	0.9939	0.0116	0.1995	0.0365
		PCH-Prior2	0.9962	0.0119	0.1374	0.0414	0.9952	0.0103	0.1910	0.0282
**AR**	$\mu_1 = 1.2$	CvxClu-L_1	0.0090	0.0898	0.7871	0.0084	0.8969	0.1166	0.3521	0.1702
		CvxClu-L_2	0.0776	0.2489	0.7641	0.0805	0.8939	0.0959	0.3481	0.1305
		PCH-NoPrior	0.8895	0.0556	0.2901	0.0669	0.9255	0.0375	0.3352	0.1101
		PCH-Prior1	0.9556	0.0408	0.2020	0.0567	0.9676	0.0282	0.2595	0.1000
		PCH-Prior2	0.9871	0.0219	0.1536	0.0449	0.9826	0.0239	0.2266	0.0972
	$\mu_2 = 1.6$	CvxClu-L_1	0.7685	0.3877	0.4881	0.3221	0.9415	0.0259	0.2585	0.0390
		CvxClu-L_2	0.9158	0.1919	0.3430	0.2068	0.9122	0.0322	0.3000	0.0434
		PCH-NoPrior	0.9762	0.0293	0.1870	0.0791	0.9841	0.0192	0.2379	0.0683
		PCH-Prior1	0.9909	0.0177	0.1426	0.0482	0.9936	0.0125	0.2009	0.0399
		PCH-Prior2	0.9967	0.0109	0.1311	0.0401	0.9946	0.0120	0.1925	0.0308
**Banded**	$\mu_1 = 1.2$	CvxClu-L_1	0.0097	0.0967	0.7841	0.0393	0.9033	0.1140	0.3325	0.1640
		CvxClu-L_2	0.0512	0.2034	0.7700	0.0757	0.9032	0.0830	0.3318	0.1139
		PCH-NoPrior	0.8888	0.0525	0.2871	0.0611	0.9269	0.0356	0.3319	0.1109
		PCH-Prior1	0.9639	0.0318	0.1842	0.0529	0.9734	0.0214	0.2488	0.0922
		PCH-Prior2	0.9861	0.0206	0.1497	0.0457	0.9836	0.0205	0.2281	0.0980
	$\mu_2 = 1.6$	CvxClu-L_1	0.8039	0.3663	0.4610	0.3079	0.9451	0.0253	0.2584	0.0381
		CvxClu-L_2	0.9519	0.1009	0.3281	0.1815	0.9180	0.0314	0.2999	0.0417
		PCH-NoPrior	0.9824	0.0218	0.1782	0.0674	0.9885	0.0146	0.2255	0.0554
		PCH-Prior1	0.9917	0.0158	0.1475	0.0486	0.9939	0.0116	0.1995	0.0376
		PCH-Prior2	0.9965	0.0115	0.1369	0.0410	0.9950	0.0105	0.1912	0.0280

Table S2: Simulation results with $(K_1, K_2) = (2, 6)$ in Gaussian clusters case. Simulation results include the mean and standard deviation (SD) of ARI of rough and refined clustering structure, MSE of $\hat{\boldsymbol{\beta}}$, and MSE of $\hat{\boldsymbol{\gamma}}$ under 100 simulated replicates with three covariance structures and two distances between cluster centers.

			**Rough Structure**				**Refined Structure**			
			**ARI**		**MSE of $\hat{\boldsymbol{\beta}}$**		**ARI**		**MSE of $\hat{\boldsymbol{\gamma}}$**	
		Methods	Mean	SD	Mean	SD	Mean	SD	Mean	SD
**Diagonal**	$\mu_1 = 1.2$	CvxClu-L_1	0.0095	0.0934	0.7830	0.0374	0.8611	0.0864	0.4034	0.1029
		CvxClu-L_2	0.0370	0.1800	0.7735	0.0681	0.8416	0.0752	0.4168	0.0804
		PCH-NoPrior	0.8954	0.0524	0.2825	0.0610	0.9147	0.0600	0.6890	0.5049
		PCH-Prior1	0.9337	0.0459	0.2258	0.0644	0.9359	0.0603	0.6250	0.4537
		PCH-Prior2	0.9704	0.0347	0.1768	0.0578	0.9579	0.0585	0.6176	0.4754
	$\mu_2 = 1.6$	CvxClu-L_1	0.8285	0.3373	0.4303	0.2964	0.9011	0.0386	0.3362	0.0476
		CvxClu-L_2	0.9463	0.1017	0.3156	0.1680	0.8590	0.0454	0.3915	0.0517
		PCH-NoPrior	0.9788	0.0263	0.1840	0.0720	0.9596	0.0656	1.0453	0.7595
		PCH-Prior1	0.9855	0.0209	0.1617	0.0579	0.9628	0.0655	1.0528	0.7854
		PCH-Prior2	0.9950	0.0128	0.1358	0.0392	0.9679	0.0649	1.0387	0.7758
**AR**	$\mu_1 = 1.2$	CvxClu-L_1	0.0904	0.2730	0.7690	0.0761	0.6582	0.1832	0.6413	0.2023
		CvxClu-L_2	0.1185	0.3079	0.7540	0.1057	0.7287	0.1478	0.5548	0.1394
		PCH-NoPrior	0.8968	0.0549	0.2777	0.0628	0.9110	0.0681	0.7220	0.5300
		PCH-Prior1	0.9380	0.0473	0.2161	0.0651	0.9349	0.0649	0.6756	0.5135
		PCH-Prior2	0.9762	0.0291	0.1667	0.0489	0.9572	0.0637	0.6494	0.5024
	$\mu_2 = 1.6$	CvxClu-L_1	0.8375	0.3268	0.4237	0.2901	0.9079	0.0427	0.3357	0.0611
		CvxClu-L_2	0.9463	0.1017	0.3156	0.1680	0.8782	0.0428	0.3800	0.0492
		PCH-NoPrior	0.9788	0.0263	0.1840	0.0720	0.9385	0.0754	0.9900	0.7252
		PCH-Prior1	0.9851	0.0220	0.1625	0.0599	0.9422	0.0744	0.9951	0.7426
		PCH-Prior2	0.9949	0.0132	0.1359	0.0392	0.9484	0.0765	0.9804	0.7448
**Banded**	$\mu_1 = 1.2$	CvxClu-L_1	0.0550	0.2192	0.7708	0.0792	0.7148	0.1861	0.5842	0.1845
		CvxClu-L_2	0.0820	0.2627	0.7570	0.1113	0.7626	0.1259	0.5228	0.1305
		PCH-NoPrior	0.8940	0.0649	0.2809	0.0737	0.9018	0.0742	0.7492	0.4854
		PCH-Prior1	0.9288	0.0631	0.2301	0.0725	0.9227	0.0716	0.6989	0.4554
		PCH-Prior2	0.9668	0.0378	0.1822	0.0584	0.9455	0.0670	0.6868	0.4687
	$\mu_2 = 1.6$	CvxClu-L_1	0.8380	0.3269	0.4284	0.2930	0.9107	0.0381	0.3303	0.0449
		CvxClu-L_2	0.9463	0.1017	0.3156	0.1680	0.8752	0.0429	0.3817	0.0491
		PCH-NoPrior	0.9788	0.0263	0.1840	0.0720	0.9623	0.0601	0.9871	0.7812
		PCH-Prior1	0.9854	0.0218	0.1614	0.0586	0.9638	0.0609	0.9907	0.8018
		PCH-Prior2	0.9945	0.0138	0.1362	0.0393	0.9712	0.0610	0.9769	0.7951

Table S3: Simulation results with $(K_1, K_2) = (3, 6)$ in Gaussian clusters case. Simulation results include the mean and standard deviation (SD) of ARI of rough and refined clustering structure, MSE of $\hat{\boldsymbol{\beta}}$, and MSE of $\hat{\boldsymbol{\gamma}}$ under 100 simulated replicates with two covariance structures and two distances between cluster centers.

			**Rough Structure**				**Refined Structure**			
			**ARI**		**MSE of $\hat{\boldsymbol{\beta}}$**		**ARI**		**MSE of $\hat{\boldsymbol{\gamma}}$**	
		Methods	Mean	SD	Mean	SD	Mean	SD	Mean	SD
**Diagonal**	$\mu_1 = 1.2$	CvxClu-L_1	0.0109	0.0747	0.9058	0.0151	0.8408	0.1101	0.4208	0.1228
		CvxClu-L_2	0.0278	0.1246	0.8992	0.0459	0.8226	0.0833	0.4351	0.0898
		PCH-NoPrior	0.8467	0.0678	0.7463	0.5241	0.8780	0.0518	0.3754	0.0572
		PCH-Prior1	0.9067	0.0722	0.6208	0.4629	0.9282	0.0530	0.3218	0.0628
		PCH-Prior2	0.9581	0.0591	0.5722	0.5060	0.9665	0.0437	0.2762	0.0548
	$\mu_2 = 1.6$	CvxClu-L_1	0.7737	0.2745	0.7085	0.3164	0.8891	0.0402	0.3487	0.0478
		CvxClu-L_2	0.9154	0.1026	0.4981	0.2194	0.8433	0.0455	0.4061	0.0520
		PCH-NoPrior	0.9610	0.0522	0.8339	0.8184	0.9689	0.0406	0.2912	0.0720
		PCH-Prior1	0.9676	0.0734	0.7949	0.8371	0.9798	0.0325	0.2634	0.0618
		PCH-Prior2	0.9804	0.0730	0.7681	0.8297	0.9914	0.0260	0.2454	0.0561
**Banded**	$\mu_1 = 1.2$	CvxClu-L_1	0.0217	0.1039	0.9035	0.0266	0.7209	0.1612	0.5849	0.1636
		CvxClu-L_2	0.0243	0.1233	0.9002	0.0431	0.7439	0.1350	0.5335	0.1282
		PCH-NoPrior	0.8388	0.0756	0.6875	0.4896	0.8726	0.0556	0.3929	0.1173
		PCH-Prior1	0.8984	0.0774	0.5711	0.4405	0.9230	0.0506	0.3386	0.1106
		PCH-Prior2	0.9538	0.0659	0.5132	0.4692	0.9657	0.0419	0.2915	0.1118
	$\mu_2 = 1.6$	CvxClu-L_1	0.7651	0.2992	0.7056	0.3250	0.8972	0.0472	0.3493	0.0530
		CvxClu-L_2	0.9064	0.1160	0.4925	0.2108	0.8557	0.0621	0.4005	0.0602
		PCH-NoPrior	0.9674	0.0281	0.7099	0.7607	0.9738	0.0229	0.2876	0.0537
		PCH-Prior1	0.9777	0.0228	0.6825	0.7767	0.9822	0.0190	0.2645	0.0430
		PCH-Prior2	0.9913	0.0149	0.6453	0.7944	0.9930	0.0114	0.2416	0.0314

Table S4: Simulation results with $(K_1, K_2) = (2, 6)$ in two half-moon clusters case. Simulation results include the mean and standard deviation (SD) of ARI of rough and refined clustering structure, and MSE of $\hat{\boldsymbol{\gamma}}$ under 100 simulated replicates with near and far centers.

		**Rough Structure**		**Refined Structure**			
		**Rand Index**		**Rand Index**		**MSE of $\hat{\boldsymbol{\gamma}}$**	
	Methods	Mean	SD	Mean	SD	Mean	SD
Near centers	CvxClu-L_1	0.7227	0.1620	0.6809	0.1536	0.4482	0.0793
	CvxClu-L_2	0.7152	0.1649	0.6346	0.1604	0.5066	0.0832
	PCH-NoPrior	0.7794	0.1443	0.8211	0.1235	0.7692	0.3951
	PCH-Prior1	0.8480	0.1993	0.8544	0.1420	0.6807	0.3384
	PCH-Prior2	0.9227	0.1844	0.8981	0.1406	0.6704	0.3502
Far centers	CvxClu-L_1	0.6998	0.1596	0.6603	0.1535	0.4626	0.0771
	CvxClu-L_2	0.6891	0.1601	0.6076	0.1601	0.5228	0.0834
	PCH-NoPrior	0.9041	0.0915	0.9120	0.0876	0.6901	0.4390
	PCH-Prior1	0.9372	0.0639	0.9302	0.0846	0.6501	0.4175
	PCH-Prior2	0.9648	0.0464	0.9460	0.0818	0.6432	0.4158

Table S5: Recovery of numbers of rough and refined clusters. In each cell, the mean of number of estimated clusters under 100 simulated replicates.

			**Rough Structure**			**Refined Structure**		
(K_1, K_2)	**Distance**	**Structure**	**No Prior**	**Prior 1**	**Prior2**	**No Prior**	**Prior 1**	**Prior2**
$(2, 4)$	$\mu_1 = 1.2$	**Diagonal**	2.00	2.08	2.03	4.04	4.25	4.25
		**AR**	2.00	2.06	2.04	4.02	4.23	4.45
		**Banded**	2.00	2.11	2.02	4.06	4.33	4.33
	$\mu_2 = 1.6$	**Diagonal**	2.00	2.08	2.03	4.00	4.10	4.15
		**AR**	2.00	2.13	2.01	4.00	4.15	4.16
		**Banded**	2.00	2.08	2.02	4.01	4.10	4.16
$(2, 6)$	$\mu_1 = 1.2$	**Diagonal**	2.00	2.21	2.05	5.84	6.26	6.04
		**AR**	2.00	2.23	2.06	5.82	6.24	5.95
		**Banded**	2.00	2.18	2.06	5.75	6.05	5.90
	$\mu_2 = 1.6$	**Diagonal**	2.00	2.12	2.06	5.81	5.97	5.89
		**AR**	2.00	2.11	2.06	5.67	5.81	5.73
		**Banded**	2.00	2.12	2.07	5.84	6.04	5.93
$(3, 6)$	$\mu_1 = 1.2$	**Diagonal**	2.98	3.14	3.08	5.96	6.21	6.16
		**AR**	2.99	3.15	3.02	5.98	6.25	6.04
		**Banded**	2.99	3.11	3.03	5.99	6.21	6.10
	$\mu_2 = 1.6$	**Diagonal**	3.01	3.26	3.03	6.02	6.33	6.05
		**AR**	3.02	3.21	3.13	6.04	6.26	6.15
		**Banded**	3.00	3.24	3.12	6.00	6.25	6.14
$(2, 4)$	**Near**	**Diagonal**	2.36	2.17	2.10	4.77	4.32	4.24
	**Far**	**Diagonal**	2.08	2.21	2.11	4.17	4.26	4.24
$(2, 6)$	**Near**	**Diagonal**	2.30	2.49	2.29	6.57	6.46	6.05
	**Far**	**Diagonal**	2.04	2.39	2.23	5.89	6.26	6.02

Table S6: Additional exploration when the clustering hierarchy is violated. Simulation results with $(K_1, K_2) = (2, 5)$ in Gaussian clusters case. Simulation results include the mean and standard deviation (SD) of ARI of rough and refined clustering structure, MSE of $\hat{\boldsymbol{\beta}}$, and MSE of $\hat{\boldsymbol{\gamma}}$ under 100 simulated replicates with three covariance structures and two distances between cluster centers.

			**Rough Structure**				**Refined Structure**			
			**ARI**		**MSE of $\hat{\boldsymbol{\beta}}$**		**ARI**		**MSE of $\hat{\boldsymbol{\gamma}}$**	
		Methods	Mean	SD	Mean	SD	Mean	SD	Mean	SD
**Diagonal**	$\mu_1 = 1.2$	CvxClu-L_1	0.0093	0.0919	0.7831	0.0407	0.5326	0.2281	0.6697	0.1561
		CvxClu-L_2	0.0629	0.2290	0.7646	0.0918	0.6185	0.1965	0.5615	0.1324
		PCH-NoPrior	0.7439	0.3364	0.3671	0.1968	0.6510	0.1095	0.5605	0.2416
		PCH-Prior1	0.6270	0.4463	0.4108	0.2718	0.6672	0.1163	0.5363	0.2148
		PCH-Prior2	0.6356	0.4616	0.3894	0.2908	0.6789	0.1270	0.5299	0.2196
	$\mu_2 = 1.6$	CvxClu-L_1	0.7385	0.4171	0.4737	0.3525	0.7709	0.1019	0.3319	0.0628
		CvxClu-L_2	0.9526	0.1021	0.3029	0.1745	0.6895	0.0632	0.3890	0.0520
		PCH-NoPrior	0.9791	0.0260	0.1832	0.0686	0.7499	0.0782	0.7849	0.4797
		PCH-Prior1	0.9870	0.0190	0.1574	0.0519	0.7516	0.0775	0.7852	0.4868
		PCH-Prior2	0.9941	0.0143	0.1405	0.0438	0.7526	0.0800	0.7791	0.4842
**AR**	$\mu_1 = 1.2$	CvxClu-L_1	0.0276	0.1583	0.7753	0.0732	0.1141	0.1541	0.9096	0.1027
		CvxClu-L_2	0.1001	0.2867	0.7526	0.1124	0.2073	0.1854	0.8375	0.1407
		PCH-NoPrior	0.7454	0.3392	0.3622	0.1996	0.6308	0.1107	0.5432	0.2288
		PCH-Prior1	0.5513	0.4666	0.4513	0.2868	0.6555	0.1457	0.5168	0.2099
		PCH-Prior2	0.5407	0.4842	0.4455	0.3076	0.6799	0.1520	0.5016	0.2107
	$\mu_2 = 1.6$	CvxClu-L_1	0.8010	0.3779	0.4626	0.3326	0.7179	0.1078	0.4342	0.1352
		CvxClu-L_2	0.9189	0.2144	0.3208	0.2157	0.6914	0.0844	0.4143	0.0932
		PCH-NoPrior	0.9815	0.0284	0.1774	0.0728	0.7345	0.0774	0.6202	0.4110
		PCH-Prior1	0.9881	0.0232	0.1599	0.0610	0.7385	0.0781	0.6172	0.4212
		PCH-Prior2	0.9922	0.0322	0.1402	0.0441	0.7393	0.0766	0.6091	0.4130
**Banded**	$\mu_1 = 1.2$	CvxClu-L_1	0.0097	0.0950	0.7859	0.0116	0.1255	0.1564	0.9104	0.0959
		CvxClu-L_2	0.0807	0.2567	0.7637	0.0805	0.2771	0.2009	0.8017	0.1355
		PCH-NoPrior	0.6780	0.3841	0.4017	0.2189	0.6196	0.1271	0.6004	0.2347
		PCH-Prior1	0.5539	0.4653	0.4535	0.2812	0.6307	0.1380	0.5701	0.2047
		PCH-Prior2	0.5911	0.4776	0.4128	0.3037	0.6451	0.1378	0.5626	0.2158
	$\mu_2 = 1.6$	CvxClu-L_1	0.8151	0.3585	0.4565	0.3162	0.7337	0.0987	0.3983	0.1223
		CvxClu-L_2	0.9648	0.0346	0.3142	0.1696	0.6938	0.0645	0.4015	0.0830
		PCH-NoPrior	0.9804	0.0266	0.1815	0.0713	0.7397	0.0789	0.7413	0.4568
		PCH-Prior1	0.9856	0.0231	0.1641	0.0599	0.7407	0.0794	0.7414	0.4645
		PCH-Prior2	0.9930	0.0314	0.1380	0.0407	0.7445	0.0781	0.7381	0.4705

Table S7: Additional exploration when the prior information is mis-specified. Simulation results with $(K_1, K_2) = (3, 6)$ in Gaussian clusters case. Simulation results include the mean and standard deviation (SD) of ARI of rough and refined clustering structure, MSE of $\hat{\boldsymbol{\beta}}$, and MSE of $\hat{\boldsymbol{\gamma}}$ under 100 simulated replicates with three covariance structures and two distances between cluster centers.

			**Rough Structure**				**Refined Structure**			
			**ARI**		**MSE of $\hat{\beta}$**		**ARI**		**MSE of $\hat{\gamma}$**	
		Methods	Mean	SD	Mean	SD	Mean	SD	Mean	SD
**Diagonal**	$\mu_1 = 1.2$	CvxClu-L_1	0.0109	0.0747	0.9058	0.0151	0.8408	0.1101	0.4208	0.1228
		CvxClu-L_2	0.0278	0.1246	0.8992	0.0459	0.8226	0.0833	0.4351	0.0898
		PCH-NoPrior	0.8467	0.0678	0.7463	0.5241	0.8780	0.0518	0.3754	0.0572
		PCH-Prior1	0.9067	0.0722	0.6208	0.4629	0.9282	0.0530	0.3218	0.0628
		PCH-Prior2	0.9581	0.0591	0.5722	0.5060	0.9665	0.0437	0.2762	0.0548
		PCH-misPrior1	0.6803	0.2053	0.6689	0.2837	0.7709	0.0567	0.5606	0.0582
		PCH-misPrior2	0.7711	0.1696	0.6505	0.3354	0.8200	0.0534	0.5330	0.0537
	$\mu_2 = 1.6$	CvxClu-L_1	0.7737	0.2745	0.7085	0.3164	0.8891	0.0402	0.3487	0.0478
		CvxClu-L_2	0.9154	0.1026	0.4981	0.2194	0.8433	0.0455	0.4061	0.0520
		PCH-NoPrior	0.9610	0.0522	0.8339	0.8184	0.9689	0.0406	0.2912	0.0720
		PCH-Prior1	0.9676	0.0734	0.7949	0.8371	0.9798	0.0325	0.2634	0.0618
		PCH-Prior2	0.9804	0.0730	0.7681	0.8297	0.9914	0.0260	0.2454	0.0561
		PCH-misPrior1	0.8172	0.0655	0.8370	0.4618	0.8207	0.0358	0.6736	0.0619
		PCH-misPrior2	0.8371	0.0683	0.8330	0.4838	0.8432	0.0270	0.6610	0.0584
**AR**	$\mu_1 = 1.2$	CvxClu-L_1	0.0167	0.0945	0.9052	0.0147	0.6312	0.1845	0.6794	0.1828
		CvxClu-L_2	0.0223	0.1089	0.9014	0.0337	0.6986	0.1455	0.5923	0.1350
		PCH-NoPrior	0.8390	0.0650	0.7736	0.5258	0.8716	0.0505	0.3854	0.0547
		PCH-Prior1	0.8998	0.0587	0.6436	0.4541	0.9209	0.0439	0.3321	0.0518
		PCH-Prior2	0.9549	0.0697	0.5963	0.5105	0.9691	0.0358	0.2803	0.0517
		PCH-misPrior1	0.6665	0.2088	0.6887	0.2842	0.7607	0.0639	0.5661	0.0582
		PCH-misPrior2	0.7416	0.2176	0.6542	0.3255	0.8110	0.0622	0.5430	0.0551
	$\mu_2 = 1.6$	CvxClu-L_1	0.7697	0.2923	0.7092	0.3111	0.9032	0.0360	0.3416	0.0446
		CvxClu-L_2	0.8952	0.1742	0.5116	0.2348	0.8632	0.0455	0.3929	0.0498
		PCH-NoPrior	0.9660	0.0357	0.8460	0.8235	0.9728	0.0292	0.2833	0.0534
		PCH-Prior1	0.9760	0.0437	0.8073	0.8379	0.9835	0.0198	0.2562	0.0413
		PCH-Prior2	0.9915	0.0144	0.7837	0.8417	0.9930	0.0114	0.2386	0.0289
		PCH-misPrior1	0.8193	0.0541	0.8491	0.4521	0.8233	0.0252	0.6717	0.0577
		PCH-misPrior2	0.8338	0.0483	0.8358	0.4713	0.8400	0.0238	0.6604	0.0511
**Banded**	$\mu_1 = 1.2$	CvxClu-L_1	0.0217	0.1039	0.9035	0.0266	0.7209	0.1612	0.5849	0.1636
		CvxClu-L_2	0.0243	0.1233	0.9002	0.0431	0.7439	0.1350	0.5335	0.1282
		PCH-NoPrior	0.8388	0.0756	0.6875	0.4896	0.8726	0.0556	0.3929	0.1173
		PCH-Prior1	0.8984	0.0774	0.5711	0.4405	0.9230	0.0506	0.3386	0.1106
		PCH-Prior2	0.9538	0.0659	0.5132	0.4692	0.9657	0.0419	0.2915	0.1118
		PCH-misPrior1	0.7079	0.1707	0.6313	0.2778	0.7669	0.0625	0.5735	0.0773
		PCH-misPrior2	0.7889	0.1448	0.5924	0.3106	0.8227	0.0419	0.5440	0.0848
	$\mu_2 = 1.6$	CvxClu-L_1	0.7651	0.2992	0.7056	0.3250	0.8972	0.0472	0.3493	0.0530
		CvxClu-L_2	0.9064	0.1160	0.4925	0.2108	0.8557	0.0621	0.4005	0.0602
		PCH-NoPrior	0.9674	0.0281	0.7099	0.7607	0.9738	0.0229	0.2876	0.0537
		PCH-Prior1	0.9777	0.0228	0.6825	0.7767	0.9822	0.0190	0.2645	0.0430
		PCH-Prior2	0.9913	0.0149	0.6453	0.7944	0.9930	0.0114	0.2416	0.0314
		PCH-misPrior1	0.8196	0.0374	0.7742	0.4215	0.8245	0.0303	0.6600	0.0675
		PCH-misPrior2	0.8378	0.0362	0.7665	0.4502	0.8424	0.0249	0.6544	0.0542

Table S8: Additional exploration under high dimensional scenarios. Simulation results with $p = 120$ and $\mu_1 = 1.2$ in Gaussian clusters case. Simulation results include the mean and standard deviation (SD) of ARI of rough and refined clustering structure, MSE of $\hat{\boldsymbol{\beta}}$, and MSE of $\hat{\boldsymbol{\gamma}}$ under 100 simulated replicates with three hierarchical heterogeneous structures and three covariance structures.

			**Rough Structure**				**Refined Structure**			
			**ARI**		**MSE of $\hat{\boldsymbol{\beta}}$**		**ARI**		**MSE of $\hat{\boldsymbol{\gamma}}$**	
		Methods	Mean	SD	Mean	SD	Mean	SD	Mean	SD
**Simulation 1**	**Diagonal**	CvxClu-L_1	0.0934	0.2874	0.7526	0.1211	0.9788	0.0142	0.2153	0.0217
		CvxClu-L_2	0.0930	0.2848	0.7315	0.1666	0.9711	0.0173	0.2359	0.0275
		PCH-NoPrior	0.8899	0.0595	0.2874	0.0702	0.9265	0.0409	0.3675	0.2059
		PCH-Prior1	0.9425	0.0457	0.2048	0.0404	0.9425	0.0403	0.2990	0.1902
		PCH-Prior2	0.9950	0.0122	0.1345	0.0309	0.9728	0.0271	0.2566	0.1964
	**AR**	CvxClu-L_1	0.0011	0.0032	0.7875	0.0071	0.9774	0.0159	0.2187	0.0310
		CvxClu-L_2	0.0892	0.2726	0.7496	0.1154	0.9662	0.0255	0.2438	0.0414
		PCH-NoPrior	0.8965	0.0671	0.2798	0.0772	0.9355	0.0428	0.3036	0.0703
		PCH-Prior1	0.9601	0.0422	0.1882	0.0671	0.9602	0.0347	0.2449	0.0505
		PCH-Prior2	0.9873	0.0161	0.1538	0.0411	0.9603	0.0319	0.2155	0.0303
	**Banded**	CvxClu-L_1	0.0011	0.0044	0.7870	0.0054	0.9825	0.0140	0.2103	0.0271
		CvxClu-L_2	0.0445	0.1944	0.7799	0.0318	0.9726	0.0120	0.2271	0.0254
		PCH-NoPrior	0.8880	0.0490	0.2900	0.0619	0.9246	0.0308	0.3281	0.0559
		PCH-Prior1	0.9550	0.0347	0.1876	0.0433	0.9552	0.0282	0.2514	0.0377
		PCH-Prior2	0.9844	0.0257	0.1483	0.0523	0.9645	0.0263	0.2217	0.0341
**Simulation 2**	**Diagonal**	CvxClu-L_1	0.0000	0.0009	0.7874	0.0060	0.9624	0.0145	0.2748	0.0253
		CvxClu-L_2	0.0001	0.0010	0.7875	0.0061	0.9453	0.0217	0.3018	0.0282
		PCH-NoPrior	0.8664	0.0608	0.3122	0.0603	0.8870	0.0707	0.7778	0.5203
		PCH-Prior1	0.9101	0.0529	0.2459	0.0632	0.9002	0.0612	0.7494	0.5237
		PCH-Prior2	0.9561	0.0302	0.1927	0.0519	0.9221	0.0657	0.7097	0.5033
	**AR**	CvxClu-L_1	0.0010	0.0030	0.7869	0.0059	0.9696	0.0193	0.2695	0.0310
		CvxClu-L_2	0.0840	0.2554	0.7466	0.1317	0.9510	0.0304	0.2997	0.0402
		PCH-NoPrior	0.8930	0.0576	0.2816	0.0620	0.9017	0.0704	0.5869	0.4054
		PCH-Prior1	0.9329	0.0537	0.2147	0.0623	0.9116	0.0648	0.5524	0.3999
		PCH-Prior2	0.9751	0.0242	0.1642	0.0374	0.9371	0.0662	0.5330	0.4039
	**Banded**	CvxClu-L_1	0.0004	0.0024	0.7877	0.0075	0.9644	0.0235	0.2777	0.0368
		CvxClu-L_2	0.0923	0.2830	0.7603	0.0859	0.9489	0.0372	0.3011	0.0428
		PCH-NoPrior	0.8885	0.0630	0.2900	0.0625	0.9033	0.0610	0.7262	0.5295
		PCH-Prior1	0.9278	0.0395	0.2322	0.0491	0.8996	0.0675	0.6941	0.5132
		PCH-Prior2	0.9599	0.0256	0.1865	0.0498	0.9266	0.0651	0.6653	0.5033
**Simulation 3**	**Diagonal**	CvxClu-L_1	0.0009	0.0017	0.9089	0.0085	0.9603	0.0191	0.2847	0.0303
		CvxClu-L_2	0.0232	0.0993	0.9088	0.0088	0.9466	0.0195	0.3114	0.0326
		PCH-NoPrior	0.8504	0.0587	0.7173	0.5373	0.8809	0.0479	0.3735	0.0486
		PCH-Prior1	0.8933	0.0520	0.6237	0.5098	0.8994	0.0470	0.3259	0.0381
		PCH-Prior2	0.9521	0.0343	0.5753	0.5325	0.9513	0.0264	0.2783	0.0292
	**AR**	CvxClu-L_1	0.0001	0.0009	0.9054	0.0047	0.9680	0.0153	0.2728	0.0242
		CvxClu-L_2	0.0007	0.0020	0.9057	0.0050	0.9513	0.0238	0.3019	0.0314
		PCH-NoPrior	0.8283	0.0653	0.7019	0.4981	0.8616	0.0536	0.3885	0.0530
		PCH-Prior1	0.8898	0.0710	0.6157	0.5170	0.9009	0.0428	0.3278	0.0447
		PCH-Prior2	0.9470	0.0445	0.5459	0.5393	0.9471	0.0315	0.2880	0.0255
	**Banded**	CvxClu-L_1	0.0008	0.0015	0.9075	0.0071	0.9668	0.0205	0.2634	0.0321
		CvxClu-L_2	0.0008	0.0023	0.9078	0.0075	0.9530	0.0288	0.2920	0.0412
		PCH-NoPrior	0.8649	0.0603	0.6482	0.5231	0.8930	0.0488	0.3581	0.0518
		PCH-Prior1	0.9260	0.0420	0.5406	0.4844	0.9219	0.0401	0.3044	0.0346
		PCH-Prior2	0.9632	0.0314	0.5130	0.5297	0.9632	0.0305	0.2701	0.0267

Table S9: Computational comparison. In each cell, the mean seconds of computational time of different methods and dimension under 100 simulated replicates with $(K_1, K_2) = (3, 6)$ and diagonal covariance structure and $\mu_1 = 1.2$.

	**CvxClu-L_1**	**CvxClu-L_2**	**PCH-NoPrior**	**PCH-Prior1**	**PCH-Prior2**
$p = 30$	145.44	122.36	286.66	298.78	303.31
$p = 120$	1798.61	1292.62	480.87	488.09	573.08

Table S10: Analysis of LUAD data. The clustering results by the proposed method. There are identified two rough clusters with sizes 203 and 152, respectively. There are four identified refined clusters nested in rough clusters, with sizes 158, 45, 118, and 34, respectively. The estimated centers of distinct rough and refined clusters are displayed with respect to imaging features and principal components.

	**Centers of Rough Clusters**	
**Imaging Features**	**1 (203)**	**2 (152)**
LymphocytesPN	-0.1120	0.1496
StromaPN	0.6094	-0.8139
TumorPN	0.3466	-0.4629
LymphocytesSN	-0.0679	0.0907
StromaSN	0.5772	-0.7709
TumorSN	-0.5648	0.7542

	**Centers of Refined Clusters**			
**Principal Components**	**1 (158)**	**2 (45)**	**3 (118)**	**4 (34)**
PC1	0.0750	0.0059	-0.0340	-0.2383
PC2	-0.0581	0.1493	-0.0107	0.1095
PC3	0.0153	-0.2853	-0.0674	0.5402
PC4	-0.2604	1.2218	-0.0022	-0.3990
PC5	-0.0097	-0.0149	0.0300	-0.0394
PC6	-0.1900	0.3570	0.1758	-0.1998
PC7	-0.0561	-0.0829	-0.0209	0.4432
PC8	0.0456	-0.2065	0.0262	-0.0298
PC9	0.1149	-0.1638	0.0430	-0.4660
PC10	0.0185	-0.4697	0.0596	0.3290
PC11	-0.1984	0.6676	-0.0349	0.1595
PC12	0.0091	0.0778	0.1021	-0.4996
PC13	-0.3119	0.6533	-0.0222	0.6618
PC14	0.0318	0.0174	-0.0956	0.1610
PC15	0.2588	0.1175	-0.2509	-0.4873
PC16	-0.0459	0.2430	-0.1693	0.4793
PC17	-0.2216	0.0919	0.2842	-0.0785
PC18	0.0018	0.3206	-0.2260	0.3518
PC19	0.0905	-0.1138	-0.1858	0.3748
PC20	-0.1526	0.1862	0.3404	-0.7188
